# Benchmarking HEp-2 cell segmentation methods in indirect immunofluorescence images - standard models to deep learning

**DOI:** 10.1016/j.compbiomed.2025.110150

**Published:** 2025-04-26

**Authors:** Balaji Iyer, Smruti Deoghare, Krish Ranjan, Bruce J. Aronow, V.B. Surya Prasath

**Affiliations:** a Division of Biomedical Informatics, Cincinnati Children’s Hospital Medical Center, Cincinnati, OH 45229, USA; b Department of Pediatrics, University of Cincinnati, USA; c Department of Biomedical Informatics, College of Medicine, University of Cincinnati, OH 45267, USA; d Department of Electrical Engineering and Computer Science, University of Cincinnati, OH 45221, USA

**Keywords:** HEp-2 cells, Cell segmentation, Deep learning, Generative adversarial network, Hybrid architecture, Transfer learning, Data augmentation, Statistical significance, Benchmarking

## Abstract

Indirect Immunofluorescence (IIF) stained Human Epithelial (HEp-2) cells are considered the gold standard for detecting autoimmune diseases. Accurate cell segmentation, though often viewed as an intermediary step to downstream tasks like classification, significantly enhances overall performance when executed with precision. In this study, we conduct a systematic literature review of HEp-2 cell segmentation techniques, identifying 28 key papers utilizing traditional image processing, machine learning classifiers, deep convolutional neural networks (CNNs), and generative adversarial network (GAN) frameworks. Building on these insights, we benchmark 17 CNN models without pretraining and 8 CNN models pretrained on ImageNet using both Frozen Encoder and Tunable Encoder strategies on the I3A dataset. Cross-validation (CV) and Benjamini–Hochberg (BH) significance correction were employed to ensure statistical rigor in model comparisons. Domain-Specific Pretraining (DSPT) experiments demonstrated performance improvements, particularly for underrepresented classes, while Data Augmentation strategies (DA-1 and DA-2) revealed distinct impacts across model categories. GAN-based segmentation experiments using the top-performing CNN architectures as generators within a Pix2Pix framework revealed performance degradation due to data limitations and adversarial training instabilities. Nonetheless, GANs displayed class-specific improvements in visual alignment of segmentation masks. Results were evaluated comprehensively across eight performance metrics, including Dice, IOU, Accuracy, Precision, Sensitivity, Specificity, AU-ROC and AU-PR. This work offers a robust benchmarking of state-of-the-art CNN, GAN, and Transformer-based models for HEp-2 cell segmentation, providing valuable insights for future research directions, including ensemble approaches, dynamic patch sampling, and diffusion models.

## 1. Introduction

Human epithelial type-2 (HEp-2) cell specimens obtained by indirect immunofluorescence (IIF) protocol is considered as the gold standard for detecting antinuclear autoantibodies (ANA) in the healthcare domain [1]. They are primarily used for serological diagnosis screening and management of autoimmune diseases such as rheumatoid arthritis, multiple sclerosis, diabetes mellitus type 1, Sjorgren’s syndrome, systemic lupus erythematosus, etc. HEp-2 cell types fall in seven cell type based classes — Homogeneous, Speckled, Nucleolar, Centromere, Golgi, Mitotic Spindle and Nuclear Membrane, and hence must be visually evaluated for specimen diagnosis. The clinical pathologists assess the cell type by the cell staining patterns and florescence intensity. However, manually identifying the stained cell patterns is a laborious and time consuming task, which is also subject to differences in expert opinion, high staining variability, errors caused by observer subjectivity and misinterpretation, poor standardization, and physician exhaustion, to list a few. Hence, there has been an incumbent need for a reliable automatic computer aided diagnosis (CAD) system that can conduct high accuracy pattern recognition for image analysis tasks such as segmentation and classification [2-5]. This would reduce the manual burden on the clinicians and augment the objective decision making capabilities of the pathologists.

Though the most popular task for HEp-2 image analysis is classification [6-18], to be able to categorize the given image into one of the seven classes for the purpose of diagnosis, segmenting out the stained cell regions of interest within the image is equally crucial to create a sophisticated high-accuracy classification model. HEp-2 cell segmentation task is generally treated as an intermediary step towards the final goal of multi-class classification. Hence, it has fewer studies focusing exclusively on foreground identification. Courtesy of many international conferences in the recent years,^2^ a lot of progress has been made in terms of novelty in the domain of automatic image analysis using traditional image segmentation models, machine learning (ML) and deep learning (DL) based approaches for HEp-2 cell segmentation from IIF images. Initial approaches involved hand-crafted features that achieved low performance due to its limited ability to extract representative features. Since the turn of the last decade, deep learning (DL) has been intensely scrutinized and extensively applied on a wide gamut of tasks across multiple data modalities [19]. The performance of these models has revolutionized several areas including computer vision and natural language processing. In the sub-domain of biomedical imaging, CNNs have outperformed many of the existing methods [20-24]. Generative adversarial networks (GANs) introduced by Goodfellow et al. [25] have also been applied for biomedical image segmentation task [26]. Recently, diffusion models [27,28] have been proposed as an alternative to GANs for generative modeling of images. In contrast to the general medical image segmentation [29,30], microscopy imaging modality represent several challenges. In immunofluorescence imaging procedure, in-homogenous diffusion of cells, blurred boundaries occur frequently. Moreover, since the imaging data represents real 3D cells via projections on a 2D plane, the varying morphology of cells represent challenges to existing segmentation methods.

In this work, our contributions are as follows.

1. Systematic review: In this work, we provide a systematic in-depth literature survey of the implemented techniques for HEp-2 segmentation. Apart from reviewing classical image processing techniques such as thresholding, active contours, fuzzy clustering we also detail the learning-based models (machine/deep learning) applied to HEp-2 cell segmentation. Our systematic literature survey across four large databases, IEEE Xplore, PubMed, Scopus, and ScienceDirect, in accordance with the PRISMA guidelines yielded a total of 28 research articles between 2008 and 2022 that had conducted HEp-2 segmentation study using either image processing technique, machine learning algorithms, deep learning models, or a combination of them. About 20 of these studies implement some variation and combination of image processing techniques like Otsu’s thresholding, watershed transformation, clustering, active contouring, fuzzy segmenting algorithm, etc. for identifying the stained cells in the images. From 2016 onwards machine learning and deep learning techniques began being implemented, and displayed promising outcomes, but were restricted by three factors — number of images in the dataset, availability of accurate ground truth masks, and ease of access to public datasets. In response to this issue, since 2013, various international conferences have released a few well curated HEp-2 datasets for classification and segmentation purposes, such as the I3A and MIVIA datasets. Hence, we observe a gradual growth in HEp-2 segmentation studies that use supervised and unsupervised approaches. Our search gave us only one paper that uses a standalone machine learning technique of random forest [31]. However, we did find a few studies exploring deep learning models for this domain [32-36].
2. Review of CNN models and GAN architectures: We examine the encoder–decoder paradigm which is fundamental to the segmentation domain and follow their evolution from FCNs in 2015 to HRNet [37] in 2019. We review not only foundational CNN architectures but also critical convolutional operations underpinning these networks along with their merits and drawbacks. Utilization of GAN has made it possible to create synthetic images and offer a more robust dataset for high accuracy training and eventually high segmentation accuracy [38]. We explore important ideas that enable GAN models to be deployed for segmentation and investigate their utility for HEp-2 cell segmentation.
3. Extensive benchmarking of CNN models: We conducted extensive experimentation to benchmark CNN models. In the first phase of our experiments we trained 17 models from scratch with parameter sweep to establish a baseline performance for the publicly available HEp-2 image dataset called I3A. We probe the efficacy of transfer learning for 8 of these models (for some of the models the concept of transfer learning is not applicable eg.: HRNet). We scrutinized the segmentation performance of these models across each type. We also investigate the utility of off-the-shelf CellPose [39] model for this dataset. For the set of 25 models obtained from the previous experiments we evaluated their performance on eight different evaluation metrics and visually examined the segmentation maps for every cell type. We observed that the ground truth mask is unreliable for many images, especially for the ones from Golgi and Mitotic Spindle classes. For the top three performing models of the 25 models (in terms of dice scores), we devised a fine-tuning procedure using RandAugment that not only boosted their performance in terms of Dice but also improved the visual coherence of the generated masks.
4. Benchmarking GAN frameworks: We examined the prospect of GAN for segmentation in this domain by deploying the top three performing CNN models as generator in a pix2pix framework [40]. To the best of our knowledge, we are the first to implement a GAN-based segmentation approach for IIF HEp-2 data and incorporate HRNet, Mobilenet-UNet, and ResNet50-UNet into the pix2pix framework and test them for HEp-2 cell segmentation.
5. Training deep learning models requires copious amounts of data is a prevailing notion in the deep learning community. While this is true in several domains and sub-fields of deep learning, we found that for the task of cell segmentation on the I3A dataset a relatively small amount of training data is sufficient to generate high quality segmentation masks. Cell segmentation though relatively easy compared to fundus or optic cup, brain tumor, multi-organ segmentation type problems, performance fluctuations can affect the clinical practices. In particular, we retained only 555 of the 1008 images in the original dataset for training (55%) which was boosted to 3885 images by random tiling and even then we were able to obtain Dice scores in the 88 to 90% range for our top models.
6. Code, Website, Models: We release the codes for the project via GitHub.^3^ Project website^4^ provides more results, corresponding segmentation masks and quantitative evaluations, rankings etc.

We organized the rest of the paper as follows. Section 2 introduces the traditional image processing techniques that have been utilized for the task of segmentation. This is followed by detailed description of various machine learning approaches and current state-of-the-art deep learning models of CNN as well as GAN for HEp-2 cell segmentation in Section 3. Section 4 reports the 25 models used for benchmarking the I3A, a HEp-2 dataset. Finally, Section 5 concludes the paper with discussions and perspectives towards pushing the envelope in obtaining highly accurate HEp-2 cell segmentation.

## 2. Available HEp-2 cell segmentation methods

We carried out a (preferred reporting items for systematic reviews and meta-analyses) PRISMA [41] style systematic literature review for the various segmentation methods that have been implemented to HEp-2 IIF images. These include standard image processing, handtuned features driven machine learning methods, and deep learning models based on convolutional neural networks (CNNs), and generative adversarial networks (GANs). We describe the segmentation methods in terms of techniques used, and experimental results.

### 2.1. Systematic review

**Fig. 1.**
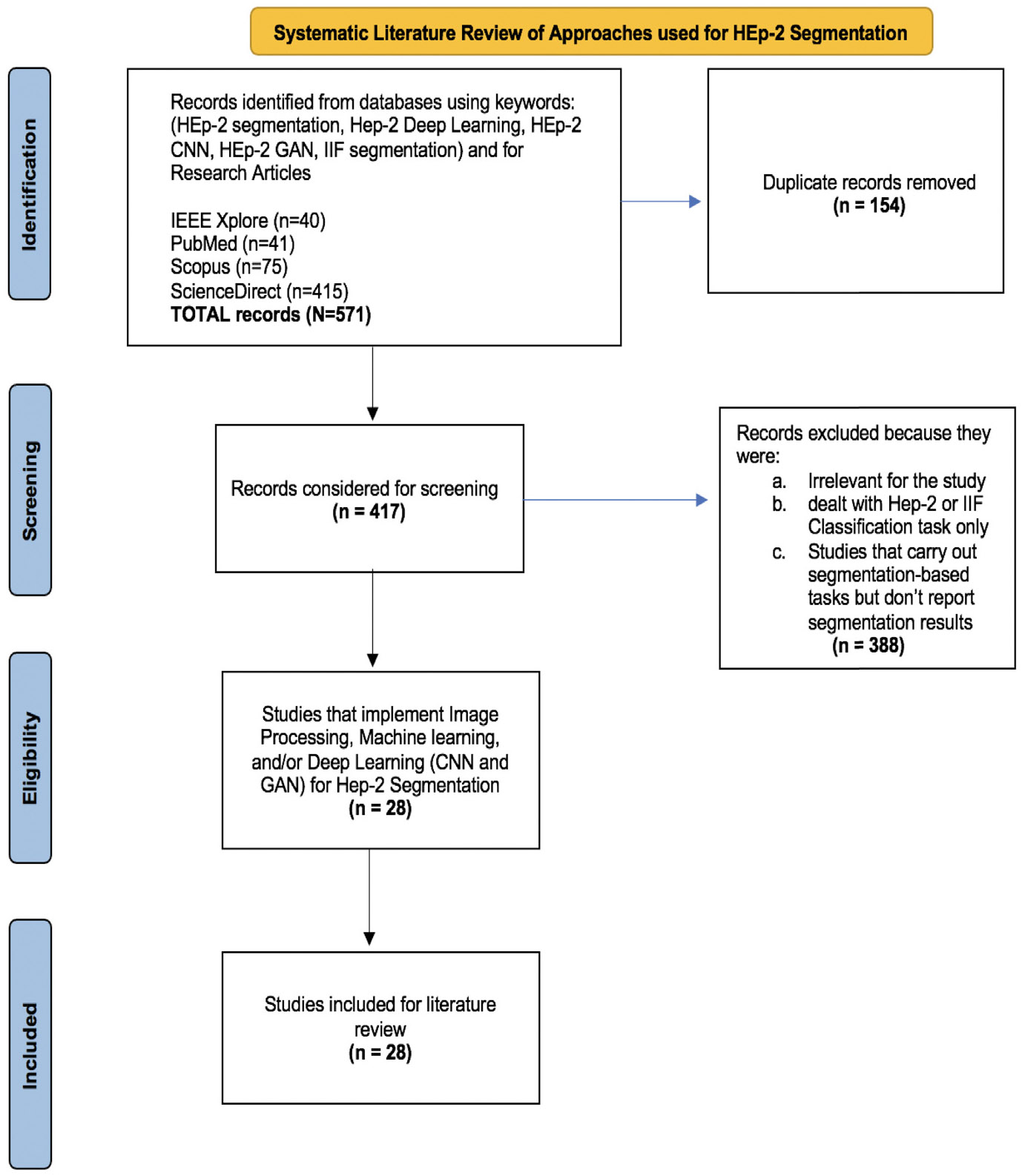
PRISMA [41] outline for studies selected for a systematic literature review.

**Fig. 2.**
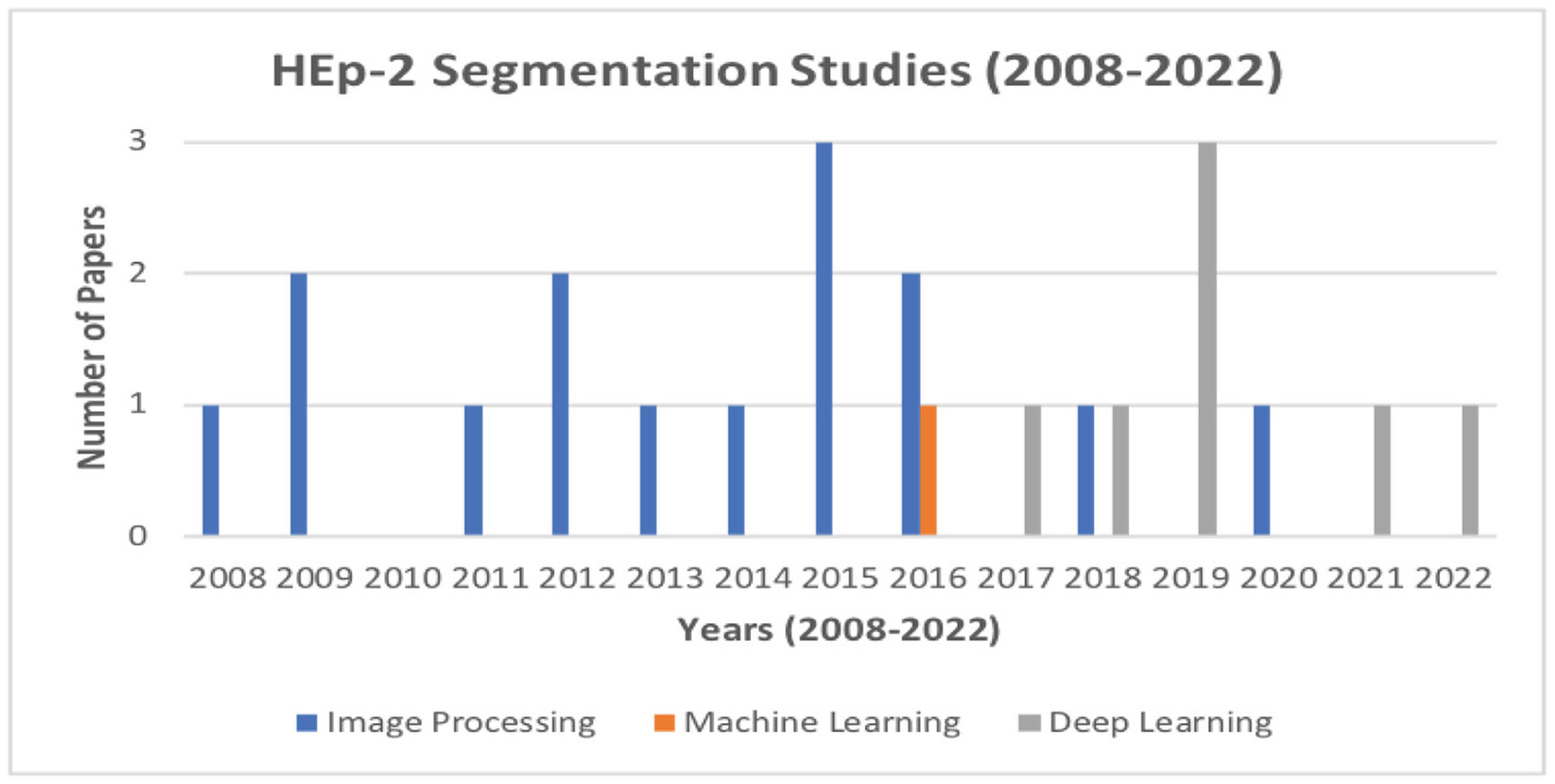
HEp-2 Segmentation studies conducted between 2008 and 2022 categorized by the techniques used — Image Processing, Machine Learning, and Deep Learning (CNNs and GANs).

Fig. 1 depicts the literature search conducted in accordance with the PRISMA guidelines. We observed that our preliminary literature search yielded no articles before 1 January 2008. All research articles between 1 January 2008 and 1 March 2022 were searched using combination of words and their acronyms to create key terms such as ‘HEp-2 segmentation, human epithelial type-2 cells, HEp-2 Deep Learning, human epithelial cells CNN, HEp-2 GAN, confocal IIF segmentation, etc’. These results were extracted from four popular databases — IEEE Xplore, PubMed, Scopus, and ScienceDirect, totaling to 571 records. These databases have overlapping search outcomes and hence the duplicates were eliminated. Articles that are irrelevant to the study and studies that dealt with HEp-2 or IIF for classification task only, or studies that do segmentation but do not report values were excluded. The articles included in the final survey were either Image Processing, Machine Learning, Deep learning CNN models, or Generative Adversarial Networks, or a combination of them, for the task of segmentation only or for a combination of classification and segmentation tasks. We finally included 28 primary research articles in the literature survey, and Table 1 provides a summary of these articles. For a quick comparison between HEp-2 segmentation approaches, we layout these papers by the year of publication in Fig. 2 to observe how the trend has changed from dominantly using image processing techniques between 2008 to 2016 to gradually moving to DL techniques 2016 onwards. However, it is surprising that we find only one study that implements a machine learning approach of random forest, in 2016 by [31].

### 2.2. Image processing and machine learning (ML) methods

Most traditional methods relied on extracting image or domain specific features using various methods for image denoising, edge detection, morphological operators etc. Although these techniques did not require a training dataset, the success of these methods relied mainly on carefully selecting the parameters for the various operations. Initial efforts in HEp-2 cell segmentation relied on classical image processing techniques and recently the deep learning (DL) models have shown better performance. Next, we review the articles in HEp-2 cell segmentation in terms of their techniques utilized, and their obtained experimental results.

#### 2.2.1. Thresholding, clustering, watershed, fuzzy, histogram, active contours

Huang et al. [42] proposed an adaptive HEp-2 cell segmentation method by combining the classical Otsu thresholding [63] and morphological techniques. This adaptive algorithm first classifies each image into one of two types and then chooses an appropriate set of parameters for each input image. Image type is determined by applying Otsu thresholding and counting the number of connected regions. A pre-defined count threshold T classifies the image as being Type 0 (sparse regions) or Type 1 (mass regions). The general scheme for both image types is to first convert the image into a suitable color space that maximizes the contrast for that image type, followed by an anisotropic diffusion filter that denoises the image while preserving the edges. After preprocessing the image, the algorithm applies canny edge detector for Type 0 images and Otsu thresholding for Type 1 images. The extracted cell outlines are then smoothed using morphological operations. Type 0 images are smoothed with a 3 × 3 diamond shaped element by alternately applying morphological erosion and dilation while Type 1 images are smoothed by applying morphological opening and closing operations with a 17 × 17 diamond shaped element. The adaptive scheme is tested using 45 IIF images containing 2573 ROIs. Typically, segmentation algorithms are evaluated using F-score. However, in this study, evaluation is done by comparing the number of cells that were delineated by their algorithm versus the number of cells manually sketched across different fluorescence patterns. Due to the small dataset and lack of a reported F-score it is difficult to compare this algorithm against other algorithms. In a follow-up study Huang et al. [43] proposed a two-stage scheme that utilized the classical watershed transformation [64] to segment HEp-2 IIF images. In the first stage, the green component of the RGB image was extracted and preprocessed. Preprocessing for the images is done by a median filter and contrast enhancement. Following preprocessing, Watershed segmentation is performed along with region merging and region elimination to prevent over segmentation. If the number of connected regions obtained was greater than a pre-defined threshold the segmentation was approved else a second stage of segmentation was performed. The second stage involved transforming the image to CMY space and applying a similar set of operations as stage 1 to the cyan component of the image to obtain reliable and stable cell outlines. An average sensitivity of 94.7% was reported across different cell types, although other metrics were not reported.

Otsu thresholding [63] performs optimally only if the image histogram displays a sharp bimodal distribution. As a consequence, the segmentation results are sensitive to object size, noise etc. To overcome these limitations, Elbischger et al. [44] proposed an adaptive and iterative variant of Otsu thresholding to segment the HEp-2 cells. In the proposed method, the image is first subdivided into blocks of size 250 × 250 and Otsu algorithm is applied to each block. A bounding box is then created around each segmented object. After this initial step, using an iterative procedure, the bounding box size is increased by 2 pixels iteratively and the Otsu algorithm is invoked at each iteration. The iterative procedure is stopped when the number of newly segmented pixels drops below 1% of previous segmentation result. The final segmentation result is obtained after applying morphological operators for smoothing. The algorithm is evaluated on a dataset of 38 images containing 982 ROIs across 5 different staining patterns. Evaluation is done by comparing the number of ground truth ROIs obtained by manual segmentation and the number of high precision (≥80%) ROIs obtained by the algorithm for each staining pattern. The average performance achieved was ≈85% across 5 fluorescence patterns.

Creemers et al. [45] proposed an unsupervised segmentation algorithm. They applied a disk-shaped morphological opening followed by Otsu thresholding, in an iterative fashion, to deal with bright cells in the image. The image histogram bimodality was restored by calculating the median of background pixels and applying it to the cells found. Every cell and cell clusters found were used as a region of interest (ROI) and iteratively treated with morphological opening and Otsu thresholding. To identify individual cells in a cluster Delaunay triangulation was applied. A total of 823 objects of size 1002 × 1004 pixels, across 7 stain patterns with 14-bit depth from a privately generated dataset were used to test this approach and achieved an average correct percentage of 89.57%.

In 2012 Divya et al. [46] attempted to solve this issue in IIF stained HEp-2 images using a combination of image open and close operations and threshold adjustments in binary images of different stain patterns. They used a small dataset of 12 images and were able to successfully use this approach to adjust the extent of segmentation. Unfortunately, this study did not use any quantitative evaluation metrics, and instead offers visual images for comparison.

Cheng et al. [48] suggested a complex pipeline for segmenting HEp-2 cells. Their method consisted of a pre-classification procedure using Otsu thresholding and checking for the number of foreground regions identified using a threshold parameter ($T_{h}$). For images with greater than ($T_{h}$) foreground regions, their method involved histogram equalization followed by morphological opening. The difference between the original image and smoothed image is then computed to obtain a marker image. Watershed segmentation is then performed on three images viz. the inverted version of the original image to obtain background segmentation, the smoothed image to obtain foreground segmentation and lastly on the marker image to obtain a marker controlled foreground segmentation. The segmented masks from three images are combined to get the final segmentation result. A similar procedure is followed for images that have less than ($T_{h}$) foreground regions. The main difference being that the marker image steps are excluded for such images since they tend to be less noisy. The algorithm was tested on 196 images that had 24 bit color depth and a resolution of 3136 × 2352. The images contained diffused, peripheral, nucleolar, coarse-speckled, fine-speckled and discrete-speckled staining patterns. They report the performance of their algorithm using Percent Volume Overlap (PVO) and Percent Volume Difference (PVD) metrics. This method achieved an average performance of ≈89% PVO and PVD of ≈22%.

Chan et al. [54] preprocessed images using color selection, run length enhancer and adaptive filtering followed by segmentation by gradient computation, contour extraction and cell splitting. The color selection algorithm essentially remaps a pixel’s RGB intensity values by binning the image histogram into six bins and then assigning a grayscale value to the pixel by projecting the original RGB value to the bin’s RGB value. This obtained grayscale image is then normalized so that the intensity values lie in the [0, 255] range. Color selection is followed by run length enhancement which further enhances the contrast in the image and reduces the halo surrounding some of the cells. The presence of halo increases the difficulty of cell segmentation. Adaptive filtering is then applied to the image to smooth the cell boundaries and fill holes within the cell body. Segmentation is done by computing the gradient for all pixels in the image and combining the gradient components at each pixel to generate a candidate segmentation image. The contours are then extracted from this candidate image by applying Otsu thresholding followed by a thinning operation to obtain sharp contours. They applied their algorithm on a dataset of 195 images and used 20 randomly selected images for evaluation. They report their findings using the following metrics, misclassification error (ME), relative area error (RAE), modified Hausdorff distance (MHD) and relative distance error (RDE). They compare the performance of their algorithm against other contemporary methods and report significant improvement in all metrics.

Tonti et al. [50] proposed a method where the green channel from the input images are pre-classified as having rough or smooth texture before image preprocessing and cell segmentation. This is motivated by the fact that during antinuclear antibody (ANA) staining, cells are typically stained with a green dye. Hence it is common to convert an IIF RGB image to grayscale by simply extracting the green channel component of the image. The pre-classification is done using Otsu thresholding followed by thresholding the average cell area in the detected ROIs. For smooth textured images, preprocessing involves histogram equalization followed by morphological opening. For rough textured images, preprocessing is done by white top-hat filtering followed by morphological reconstruction and histogram equalization. Marker extraction is then performed by applying an adaptive fuzzy c-means (FCM) clustering [65] followed by Randomized Hough Transform (RHT). FCM roughly separates the background and foreground regions. The objective of applying RHT is to divide candidate clusters into elliptical sub-regions. The most probable location of individual cells are the centers of the ellipses identified by the RHT decomposition. A marker-controlled watershed segmentation is utilized to obtain the final segmentation. They test this method on MIVIA dataset and achieved an F-score of 71.04%.

In most HEp-2 IIF images an irregular intensity distribution can be observed, hence segmenting with a global threshold produces poor results. Jiang et al. [49] proposed a method to overcome this challenge. They employed a hypotheses generation and verification paradigm in their proposed knowledge-guided adaptive thresholding scheme. Initially the input image is thresholded with a range of thresholds and verification procedure is applied to each region in the segmentation map produced by each threshold. The verification procedure is implemented by computing an area filter to ensure clusters are not too big or too small, shape filter to determine the ellipticity of the cluster and a position filter to determine overlapping cluster regions. The verification procedure is highly dependent on the known information about the objects of interest. The method was tested on MIVIA dataset and achieved an F-score of 72.5%.

To deal with irregular intensity distribution in the task of Hep-2 cell segmentation Banerjee et al. [55] proposed a new probability distribution called the stomped normal (SN) distribution. In intensity distribution, each class has a specific lower approximation and a probability boundary region. Using SN distribution and the finite mixture (FM) model, an SNFM segmentation approach was used on 16 of the 28 images in the MIVIA dataset and achieved an average F-score of 85.93% compared to finite Gaussian mixture (FGM) and non-rough counter part (NRS), and an average F-score of 75.06% compared to fuzzy c-means (FCM), robust rough-fuzzy c-means (rRFCM), and rough-fuzzy c-means (RFCM). Following the SNFM segmentation method, in 2016 Banerjee et al. [56] proposed the rough-probabilistic clustering and hidden Markov random field based segmentation method (RPrCM) in 2016 and successfully applied it on HEp-2 images and brain MR images dataset, for various bias fields and noise levels. RPrCM performed with an average F-score of 85.47% on 18 of the 28 MIVIA HEp-2 dataset, when compared with other clustering techniques such as hard c-means (HCM), FCM, FGM, RFCM, rRFCM, rough probabilistic clustering (RPrC), Gaussian distribution and HMRF model based segmentation algorithm (GHMRF), and NRS.

Zhou et al. [51] proposed an adaptive local thresholding technique. They used a 15×15 overlapping sub-images with a sliding window to create a threshold map by comparing the sub-image standard deviation with the enhanced cell images. Based on select distance parameters and the nearest non-one replacement, the binary threshold map is refined and then linearly interpolated to original image size. As part of the post-processing, the small size morphological noises were removed with open operation followed by watershed transformation to separate the connected cells. This cell segmentation framework resulted in a 66.95% segmentation accuracy on the publicly available ICPR2014 HEp-2 dataset.

Khamael et al. [53] proposed an automatic segmentation method which uses level set methods via geometric active contours [66]. Cell foreground regions are determined by using morphological opening and closing operations. Zero level set is initialized. A level set function defined as the signed distance between a given pixel position and the zero level set is initialized. The level set function is evolved using a geometric active contour (GAC) model using a force with normal direction. Using this method they were able to achieve a Dice score of 80% on a dataset 1001 images of size 1388 × 1040.

Merone et al. [52] utilized the active contour without edges method [67]. The input image is preprocessed by histogram equalization followed by Otsu thresholding to generate an over-segmented image. A bounding box is generated around each candidate cluster. The final segmentation map is generated using an active contour model by minimizing a modified energy functional. The active contour method which is applied on the pre-segmented mask, minimizes the energy functional to generate the contour around each foreground region. This method is able to achieve a Dice score of 84% on a subset of 18 images from MIVIA dataset.

Riccio et al. [60] proposed an unsupervised framework for identifying the foreground region from the background and then counting the cells, which they named as Cell Segmentation and Counting (CSC). They proposed two different scenarios of the framework, one where they tune all seven parameters, CSC-7, and other where four of the seven parameters are automatically computed, CSC-3. For the purpose of segmentation of the foreground from the background, gray level clustering is applied on thee overlapping square patches followed by adaptive thresholding to create a binary mask, which is merged with patches to create the segmentation task output images. Next task of cell counting focuses on detecting the center of the cells using the combination of distance transform and foreground boundary curvature analysis. This study utilized a combination of IIF datasets from two sources, MIVIA and four sets from Broad Bioimage Benchmark Collection (BBBC) of real and synthetic nature. They achieved a Dice score of 85.2% for CSC-7 and 85.1% for CSC-3, which is higher than the segmentation results of [31] of 84.2% and [53] of 80.2%.

Roy and Maji [57] modified the classical rough-fuzzy clustering by imposing a spatial constraint to segment HEp-2 cells in IIF images. The method first applied Otsu thresholding to an input image to generate candidate clusters. The class labels of the pixels surrounding the clusters are determined by a spatially constrained fuzzy membership function. This method performs better when compared to other fuzzy based methods and achieved a F-score of 86.8% on the MIVIA dataset. Following this approach, Roy and Maji [59] incorporated the much ignored neighborhood information with the rough-fuzzy clustering approach in the rough-fuzzy segmentation (RouFS) algorithm, where pixel class is dependent on the spatial and gray level constraints of the neighboring pixels. On the MIVIA dataset with 28 HEp-2 images, RouFS performed with 83.42% F-score. In 2020, Roy and Maji [33] expanded the applicability of segmentation technique to other medical images beside HEp-2 cell images. Like RouFS, the proposed spatially constrained rough-fuzzy c-means (sRFCM) for medical image segmentation uses the knowledge of local neighbor pixel labels to label the central pixel, thus normalizing as a consequence. This algorithm lowers the boundary region and probabilistic approximations to give cluster centroids. Furthermore, the work proposed a segmentation validity index called neighborhood Silhouette to identify the regularizer value and weight parameter. This framework achieved an average Dice score of 86.1% for 28 images of MIVIA public dataset.

Islam et al. [34] attempted automatic HEp-2 cell segmentation using a modified Sliding Band Filter (SBF) technique which could handle the varying intensity, low contrast and noise in the IIF images. After k-means clustering and morphological processing, the images were used as a mask. SBF was deployed to detect even the cells have high variance and low contrast, followed by a post-processing to identify the cells with high accuracy. When employed on MIVIA 28 image dataset with 1582 cell objects, it achieved an average Dice score of 74.62%.

For the interested reader, we have provided literature review for papers from 2022–2024 in the Supplementary.

#### 2.2.2. Machine learning

Most segmentation algorithms in this domain tend to perform poorly when cells exhibit large variance in intensity within the cell body. Percannella et al. [47] proposed a classification based approach to overcome this limitation. They observed that the image intensity histogram can be divided into 3 bands, with pixels with lowest and highest intensity values assigned to foreground and background respectively, with very less uncertainty. These pixels were considered as labeled data and were identified using image reconstruction algorithm. Contextual information of each image is exploited by training a classifier on the labeled portion of the image. Auto-learning method with two different kinds of classifiers, namely multilayer perceptrons (MLP) and nearest neighbor (NN), were tested. Finally, the classifier is used for predicting the class labels of the pixels in the third band. The authors report the performance of their algorithm on the MIVIA dataset which contains 28 annotated IIF images, with resolution 1388 × 1038 pixels and 24 bit color depth. They report the performance of the algorithm at the pixel level and cell level and compare its performance with other methods. They were able to achieve a average Dice score of 59.8% at pixel level and 56.8% at cell level. One downside of their algorithm is that for each new image a classifier has to be built which increases the computational complexity of this method.

Prasath et al. [31] expanded the multiscale feature bank [68] with a random forest (RF) classifier that was used successfully in epifluorescence microscopy vessel segmentation [69] to augment it with feature operators sensitive to sub-cellular patterns. They reported improved cell classification and cell segmentation results with the average of F-score of 84.26 on the ICPR 2016 dataset (1008 images).

### 2.3. Deep learning (DL) methods

#### 2.3.1. Convolutional neural network (CNN) models

Despite the success of deep learning (DL) models in the image classification tasks, image segmentation is still growing, especially in domains like HEp-2 cells where the images have several patterns. The fully convolutional network (FCN) based on end-to-end and pixel to pixel trained model performs better than traditional segmentation techniques in many domains. Li et al. [58] proposed a fully convolutional residual network (FCRN) that simultaneously addressed the classification and segmentation task of HEp-2 IIF images on the I3A-2014 dataset. The model consisted of 3 bottleneck modules followed by 13 residual-in-residual (RiR) modules. Each bottleneck module contained 3 convolution layers while each RiR module constituted 6 convolutional layers. This was a very deep network with a total 88 convolutional layers and 4 deconvolution layers. The authors experimented with 3 different data augmentation techniques involving different rotation angles and mirror effect and report the results for each case. The RiR modules proposed in this paper are inspired by the bottleneck modules of ResNet-50 architecture. Similar to the bottleneck module, the RiR modules incorporate skip connections and aim to alleviate the vanishing gradient problem in very deep neural networks. The model is trained and tested on the I3A dataset that contains 1008 images taken from 252 specimens. Each image in the dataset belongs to one of seven classes depending on the staining pattern visible in the image. During training the model makes dense prediction for each pixel in 8 categories i.e. the 7 cell classes and background region. During testing each image is divided into 4 × 4 sub-images, the FCRN model predicts the class segmentation mask for each sub-image. The predictions of the FCRN model across all sub-images are assembled and the class with the highest population is declared as the class label for that image. This model achieved an F-score of 89.03% on the segmentation task and mean class accuracy of 94.94% on the classification task. Although the very deep FCRN [58] can give outstanding results, the accuracy drops in case of transfer learning.

The encoder–decoder architecture has become very popular in the recent past. The convolution layers in the encoder progressively discover higher level feature descriptors useful for context summarization but will loose spatial information of the features while the decoder tries to recover the local information from the higher level features through the process of deconvolution. In other words, the encoder captures what is present in the image while loosing where they are located on the image whereas the decoder tries to locate the artifacts corresponding to the features discovered by the encoder. Therefore, there is a trade-off between localization and context understanding. The U-Net architecture [21] inspired from fully convolutional network (FCN) mitigates to a certain extent the trade-off between localization and context understanding. The key innovation in U-Net architecture was to use lower level features discovered by the encoder to guide the process of deconvolution. As with all deep neural networks, network depth is constrained by the vanishing gradient problem. Residual blocks introduced in He et al. [70] help to solve this problem. A U-Net like architecture with short and long residual connections was proposed in Quan et al. [71] for segmentation of microscopy images. As we will show in experiments (Section 4.2) U-Nets can be used flexibly with different encoder architectures and are able to segment HEp-2 IIF images with Dice score of >80%.

HEp-2 cell staining patterns are of seven types — homogeneous, speckled, nucleolar, centromere, golgi complex, nuclear membrane, and mitotic spindle. Mitotic spindle stain type is a rare occurring one. In 2019 Gupta et al. [61] proposed a framework for specifically identifying and then segmenting mitotic spindle type staining. This deep convolutional neural network segmentation based classification approach employed U-Net for a pixel-based segmentation on gamma transformed images from the I3A Task-2 dataset, pre-trained from scratch on the DAPI-channel based masks. The average Dice score achieved for three random cross-validations was 0.96. Following the high segmentation accuracy, two classifiers, Support Vector Machine (SVM) and CNN based baseline classifier performed with overall high Dice score of 96%.

Xie et al. [32] proposed an end-to-end Deeply Supervised Fully Convolutional Network (DSFCN) that uses dense deconvolutional layer (DDL) to fuse various layers of feature graphs to maintain high resolution images and hierarchical supervision structure (HS) to optimize the feature information captured from the shallow layers. This framework did not require any prior knowledge or post-processing. The 1008 grayscale specimen images from the I3A-2014 dataset were enhanced by mirroring, cropping, and rotating, to avoid overfitting issue, resulting in 241 920 images. This approach achieved the segmentation accuracy (SEG) of 90.10%, sensitivity (SE) of 89.96%, Jaccard index (JA) of 82.68%, and Accuracy (AC) of 96.56%. Jiang et al. [35] proposed a strategy that used a mask R-CNN with ResNet-101 as the backbone with pretraining on the COCO dataset and traditional augmentation like brightness contrast adjustments and flipping images horizontally and vertically, in order to segment HEp-2 stained cells. A region proposal network (RPN) creates a bounding box, which is followed by feature extraction in the bounding box region. Upon classification, the redundant bounding box is eliminated and a mask is generated. This mask is aligned with the corresponding RPN stage image and used in the segmentation modelThe study used an in-house ANA IIF HEp-2 dataset of 333 images with 1392 × 1040 resolution and 3 cell type categories of metaphase, interphase, and undetermined. These images were collected between January 2018 to December 2019 at the Taichung Veterans General Hospital, Taiwan. They report a segmentation accuracy of 89.08% and cell cycle classification accuracy of 95.07%.

#### 2.3.2. Generative adversarial network (GAN) models

Generative adversarial networks (GANs) introduced in [25] was a disruptive idea that further stimulated divergent thinking in the research of deep neural networks. Initially, the primary application of GANs was in data augmentation where the generator model of GAN would use noise as input and produce realistic training data. The introduction of conditioning variables to GAN expanded its applicability to other tasks like image segmentation, style transfer [72], image translation [73], etc. Li and Shen [26] combined AC-GAN [74] and pix2pix [40] GAN models for segmenting cells in HEp-2 images. In AC-GAN, in addition to predicting if a generated image is real or fake the discriminator also predicts the class label of the generated image. Thus, the AC-GAN is trained with a classification loss in addition to the adversarial loss, while a pix2pix model is trained using L1 loss and adversarial loss. Li et al. [26] proposed GAN based transfer learning framework called cC-GAN with RU-Net, a modified U-Net with residual blocks, as the generator and trained a GAN model with all three losses viz. adversarial loss, classification loss and L1 loss. To improve the stability of the network, they pre-trained the generator using just L1 loss and then fine-tuned the generator by the typical GAN training process. This model achieved a Dice score of 86.15% on the I3A dataset. An I3A pre-trained model was fine-tuned on the MIVIA dataset and was able to achieve 79.89% Dice score on the MIVIA dataset. Whereas, without fine-tuning the model achieved 75.27% Dice score on the same dataset.

Xie et al. [62] created a hybrid framework for the combined tasks of HEp-2 segmentation and classification. The segmentation section of the framework contains a generator consisting of DeepLabV3+ [75] and Xception [76], and discriminator composed of convolutional layers and batch normalizations. The original and corresponding synthetic image pairs are used as input for the ResNet-50 [70] based classification task. The proposed model achieves a Dice score of 96.40% on the ICPR 2016 HEp-2 images dataset for segmentation. Recently, Xie et al. [36] devised a hybrid network architecture for the segmentation and classification of HEp-2 cell images. A GAN architecture was deployed which segmented the cell objects from the images using ResNet-34, following a binary classification of real vs. not real by the discriminatory, and finally followed by the six class classification by augmented channel MobileNetv3 (ACM-Net) network. The ICPR2016 Task 1 dataset achieved an average Dice score of 97.04% for segmentation and 98.82% for classification.

## 3. Benchmarking DL models for HEp-2 cell segmentation

### 3.1. CNN models

The number of deep learning segmentation models is far too numerous to experiment on all of them. Almost all deep learning based image segmentation models utilize the encoder–decoder framework. The encoder compresses the spatial representation of an image into a dense latent representation with loss at the cost of localization information which the decoder attempts to recover using upsampling. In this study, we consider three main family of models viz. Fully Connected Networks (FCN) [20], U-Net [21] and High Resolution Networks (HRNet) [22]. In addition to the classical FCN networks, we also consider popular variants of the FCN architecture viz. PSPNet [23] and SegNet [24].

**Fig. 3.**
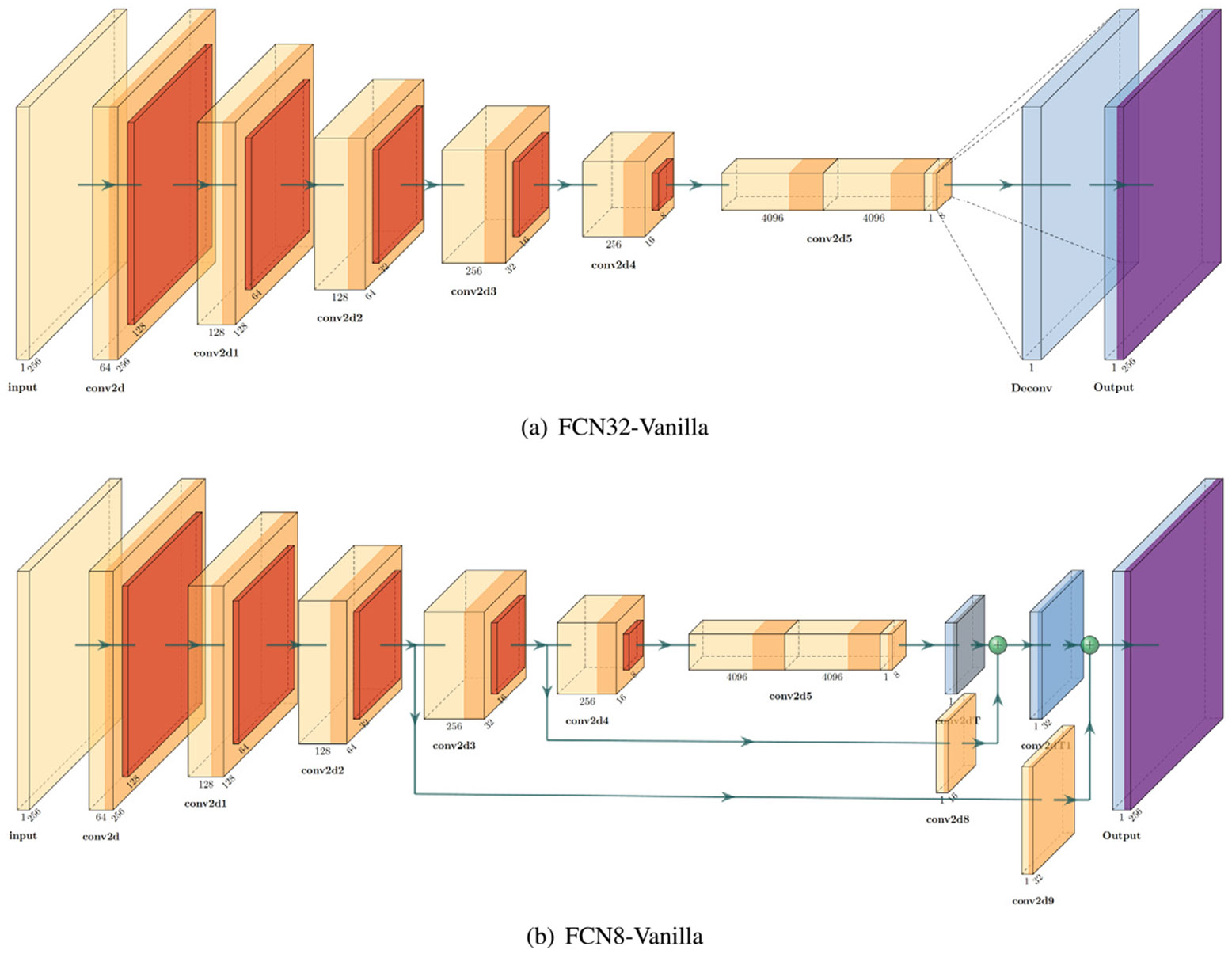
Canonical architectures of FCN32 and FCN8 models. FCN32 is the fundamental architecture for all encoder–decoder networks.

Early deep learning models for semantic segmentation performed semantic segmentation by identifying regions of interest within an image and classifying them as belonging to object of interest/foreground or background. The final segmentation map was obtained by stitching image patches to obtain object boundaries within the image. Shelhamer et al. [20] proposed a novel encoder–decoder architecture that enabled pixelwise prediction for semantic segmentation. The encoder in the FCN-32 model shown in Fig. 3(a) reduces the spatial dimension of the input from 256 × 256 to 8 × 8 while increasing the channel dimension from 1 to 4096. The latent representation is obtained by convolving the 8 × 8 × 4096 with a single convolution filter to obtain 8 × 8 × 1 feature map. One can view the input image as a map that contains spatially rich information but poor feature information. By contrast, the 8 × 8 × 1 representation obtained at the end of the encoder contains higher order feature information at the cost of spatial resolution. Each pixel in an image captures local information of the object independent of other pixels, while the feature map carries rich higher order information about the relationship between different pixels. Feature maps generated at each convolutional block from the input capture progressively higher order features. The challenge of semantic segmentation is to recover object localization information using higher order feature maps despite the loss of spatial resolution. Encoders typically use strided convolutions or max pooling for downsampling, while upsampling can be achieved using non-parametric methods like bilinear interpolation or by use of transpose convolutions. Transpose convolution is essentially scaling the kernel at every position of the input matrix and summing the overlapping values to produce an upsampled output. Transpose convolutions can sometime produce checkered board artifacts due to uneven overlap. Despite this limitation, the parametric nature of transpose convolutions tends to produce better results over other non-parametric upsampling techniques. In the FCN-32 model, the 8 × 8 latent representation is scaled to 256 × 256 spatial dimensions using transpose convolutions, and the final segmentation map can be obtained by performing a pixelwise softmax operation on this feature map. The FCN-32 model is named so for the fact that the latent map (8 × 8 in our case) used for producing final output is 32 times smaller than the input (256 × 256 in our case).

**Fig. 4.**
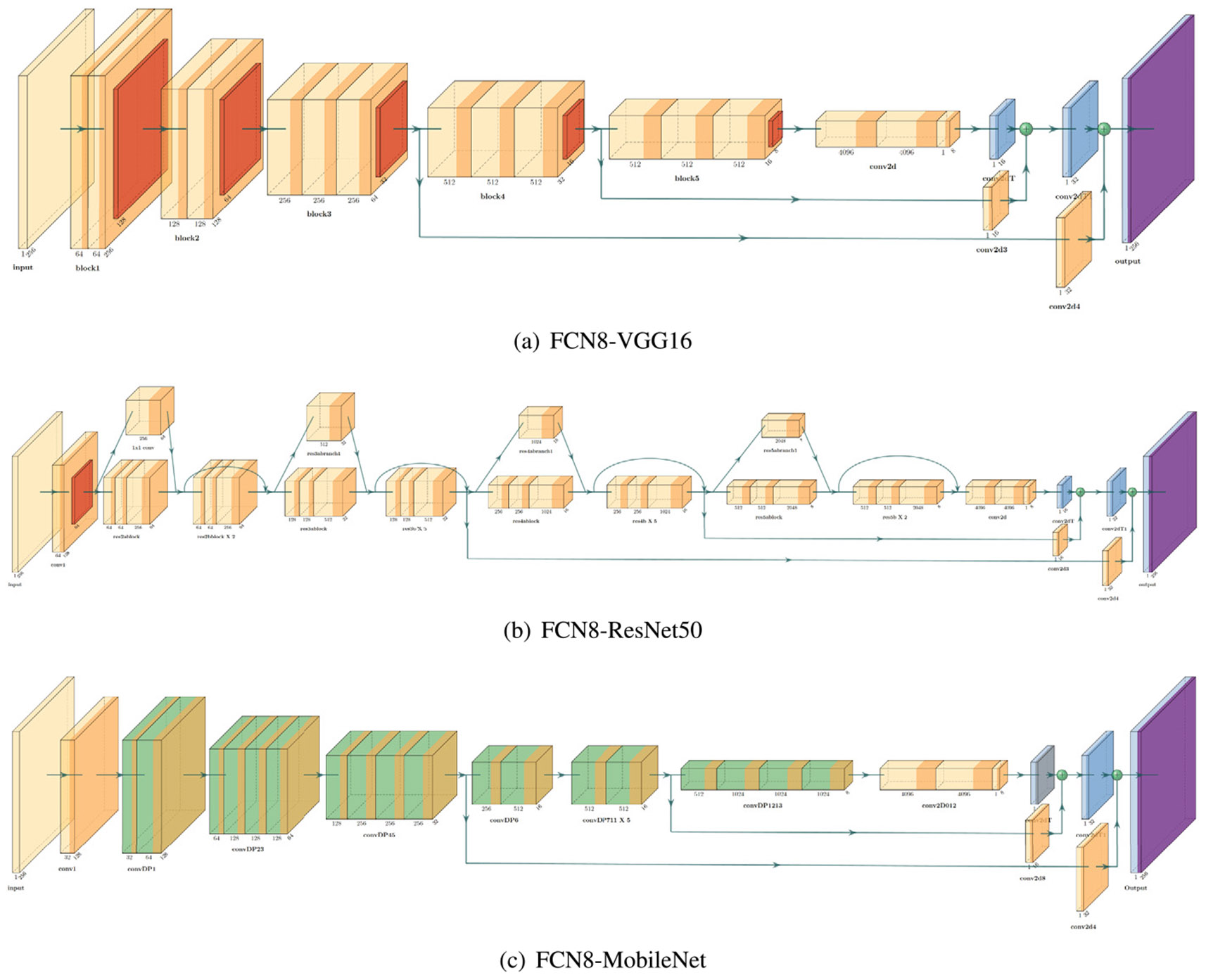
Variants of FCN8 model obtained by replacing the encoder section with other network architectures.

Fig. 3(b) depicts the FCN-8 model which was also introduced in the same paper [20] as FCN-32. Here, the feature maps from intermediate layers were added to the upsampling maps after applying suitable convolution operation for dimension matching. The flexible and modular nature of the encoder–decoder architecture is ideally suited for further research in this domain. Changes could be made to the encoder and decoder independent of each other. In the segmentation domain, pretrained encoders are popularly utilized in place of untrained encoders. Fig. 4 shows three popular encoders used with FCN models viz. ResNet50 [70], VGG16 [77], and MobileNet [78] respectively. VGG16 uses 3 × 3 kernels throughout the model and while the spatial dimension is halved at every layer, the number of filters are doubled. ResNet50 model is characterized by skip connections that allow gradients to backpropagate faithfully, alleviating the problem of vanishing and exploding gradients. MobileNet employs depthwise separable convolutions. The standard convolution is a single operation with a kernel whose channel dimension is equal to the channel dimension of the inbound layer. By contrast, depthwise separable convolutions is split into two operations. The first convolution applies independent kernels for each channel of the inbound layer to obtain feature maps with the correct spatial dimension. The second convolution known as pointwise convolution applies 1 × 1 convolution on the feature maps obtained from the previous step to produce the outbound feature maps for the layer. Depthwise convolution performs a filtering operation on a particular channel of the inbound layer whereas pointwise convolution combines features across channels to produce new features. Compared to standard convolutions, depthwise separable convolutions are far more efficient computationally since they involve fewer parameters and multiplications. However, a drawback of depthwise separable convolutions is the lack of expressive power compared to standard convolutions.

**Fig. 5.**
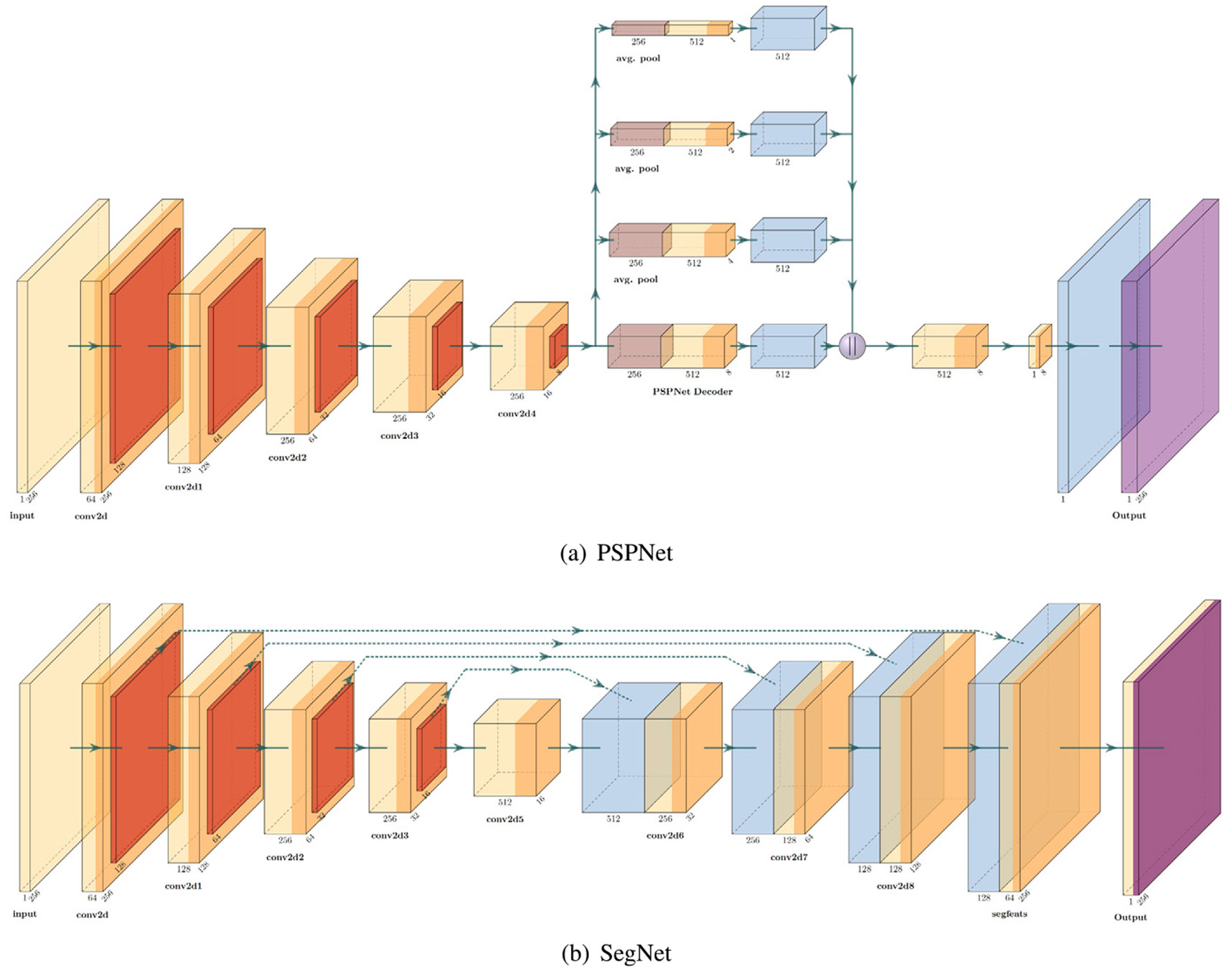
PSPNet and SegNet models are variants of FCN8 with upgrades in the decoder section.

Over the years, many variants of FCN were proposed that involved changes to the decoder. In our experiments, we consider two popular networks viz. PSPNet [23] and SegNet [24]. In PSPNet, refer Fig. 5(a) the latent map of the encoder is average pooled at multiple scales to produce dense feature maps of different granularities. This is followed by a convolution and upsampling using bilinear interpolation. The multi scale pooled features are then concatenated, followed by convolution and again upsampled to produce the final segmentation maps. The SegNet decoder shown in Fig. 5(b) utilizes an inverted VGG16 motif where the spatial dimension is doubled and channel dimension is halved at every layer. SegNet proposed a novel upsampling technique known popularly as Max Unpooling where the pooling indices from corresponding max pooling operations (dashed lines in Fig. 5(b)) are used to produce sparse upsampled feature maps. These sparse maps are then densified using standard convolution operations.

**Fig. 6.**
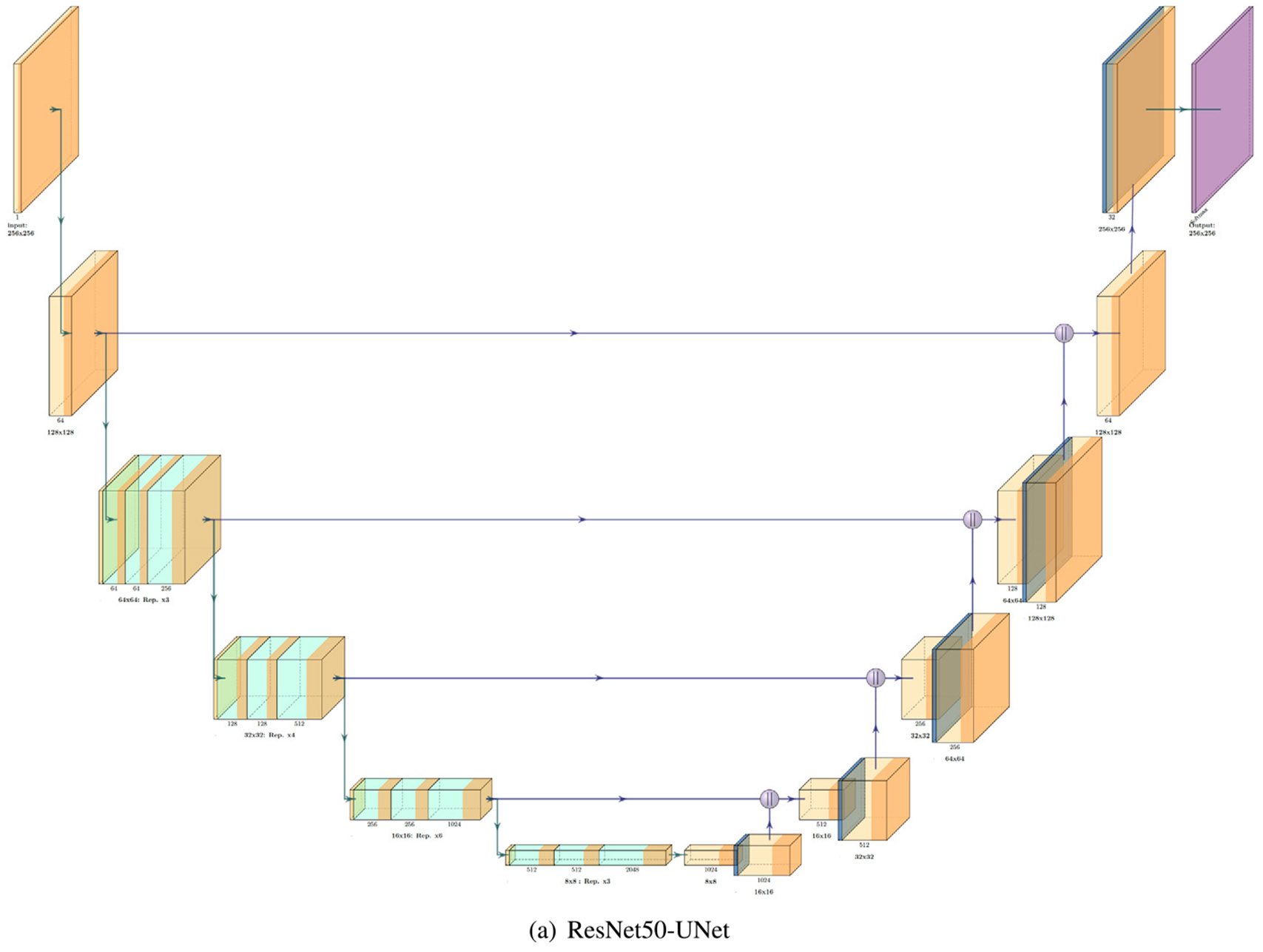
U-Net architecture with ResNet50 encoder. Note that the encoder (left half of the U) is replaced with other network architectures. For this study, we considered U-Net models with VGG16, ReseNet50 and MobileNet encoders.

In 2015 Ronneberger et al. [21] proposed U-Net, which is thematically similar to SegNet. The key idea in U-Net is the concatenation of feature maps across corresponding layers of the encoder and decoder arms. This sharing of information is key to mesh high resolution lower semantic features learned in the upper convolutional layers with the low resolution higher semantic features learned in the lower convolutional layers. Hundreds of variants of U-Net have been proposed since its introduction. In this study we consider three popular encoders to be used within the U-Net framework, namely VGG16, ResNet50 and MobileNet. Fig. 6 shows the ResNet50-UNet architecture. Within the biomedical imaging domain, U-Nets are considered as the gold standard for segmentation tasks due to the modular and reusable nature of the architecture coupled with the exceptional results they have produced on segmentation tasks across multiple imaging domains. We also consider CellPose [39] which proposed a modified U-Net, by using residual blocks instead of the standard building blocks, for predicting horizontal and vertical gradients, as well as whether a pixel belongs to a cell in the image. Then these gradients are combined to construct a dynamic system that computes segmentation masks.

**Fig. 7.**
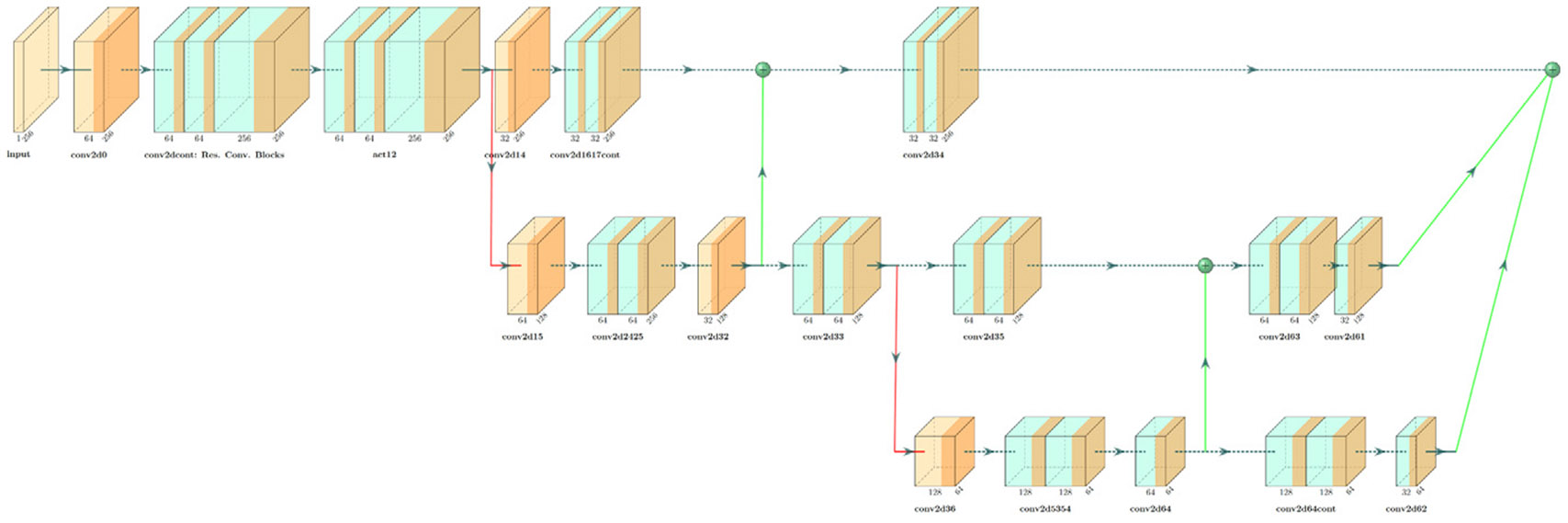
HRNet architecture, a radical departure from the encoder–decoder architecture.

Finally, we consider HRNet [37] which utilizes a typical multi resolution lattice structure, popular in the wavelet community. In contrast to FCNs and U-Net, HRNet strives to maintain high resolution representations across the network by repeated fusion of low and high resolution feature maps, as shown in Fig. 7. In each stage of the network, the number of features are doubled while the spatial dimension is halved. Thus, prior to fusion, a convolution and upsampling operation is needed to match the dimensions of the target stage. HRNet has been successful in multiple vision tasks like pose estimation, segmentation, classification, etc. However, one drawback of HRNet is that it cannot be adapted easily with other pretrained networks or architectures.

### 3.2. Transformer models

Since the introduction of Transformer in 2017 by Vaswani et al. [79], they have become the cornerstone of state-of-the-art Natural Language Processing (NLP) applications. It has catapulted the development of pre-trained language models like BERT [80], GPT (Generative Pre-trained Transformer) [81], and others. The self-attention mechanism, a pivotal element of transformer models, plays a crucial role in adaptively weighting each word’s significance within the context of a sentence. The self-attention mechanism empowers the model to effectively capture essential contextual dependencies, enabling a deeper understanding of semantic relationships within sentences. By stacking multiple transformer layers, these models gain the ability to efficiently capture document-wide interactions, thereby facilitating a more comprehensive analysis of longer texts. The interplay of self-attention and layer-wise stacking bestows flexibility to these models and enhances their effectiveness in a wide range of NLP tasks.

The remarkable success of the transformer architecture in NLP spurred extensive research in the domain of Computer Vision, with a focus on exploring and adapting the core principles to enhance performance in vision-related tasks. Dosovitskiy et al. pioneered the idea of Vision Transformers (ViT) [82] with the key concept of dividing an image into non-overlapping 2D patches which are flattened into a sequence of patches. This sequence of patches can then be fed into a transformer encoder. The principal assumptions of CNN’s and Transformers are starkly distinct. The main inductive biases of CNN’s are 1. Spatial Locality: Assumes semantic relationship between spatially local pixels. 2. Translational Equivariance: The translational shifting of an object in an image causes similar shift in the feature activation map but does not alter the activation itself. This property is crucial to learning spatially invariant features. 3. Hierarchical Structure: Image features have a natural hierarchical ordering i.e. semantically complex and abstract features are composed of simpler low-level features. Lower level CNN layers thus capture low-level features and deeper layers are capable of capturing complex semantic features. The underlying assumptions of Vision Transformers are 1. Self-attention: ViT’s learn the relationships between all patches in an image simultaneously using self-attention mechanism, catalyzing the capturing of contextual dependencies between the different patches an image. 2. Sequential processing: The loss of geometric structure due to treating images as sequences forces ViT’s to assume that the position and order of patches provide crucial information. 3. Loose Translation Equivariance: In contrast to CNN’s, translational equivariance is learned from the data by capturing relationships between patches. Given the distinct and complementary inductive biases of CNNs and ViTs, it becomes a compelling direction in research to explore their combination as a means to synergistically enhance the performance of these models. In particular for the task of semantic segmentation, it is imperative for the model to capture semantically complex features and relationships in the image without losing localization information of those features. In our study, we considered TransUnet [83] and SegFormer [84] two of the most popular transformer based segmentation models.

The TransUnet model embodies an innovative hybrid architecture that seamlessly blends the Transformer layers into the well-established Unet framework. The Convolutional Neural Network (CNN) component serves as a robust multi-scale feature extractor, efficiently capturing spatial information from the input data. At the highest level of abstraction, the feature maps are tokenized into patches, paving the way for 12 consecutive transformer layers to operate on these patch embeddings and effectively capture global context information. This novel combination of CNN and Transformer empowers the model to leverage both spatially local dependencies as well as global contextual dependencies, enhancing its ability to comprehend complex patterns and relationships within the data. The decoder path harmoniously integrates contextual embeddings of the Transformer layers by reshaping it to align with the input CNN layer’s shape, enabling seamless integration of high-level contextual information with high-level feature maps. The decoder proceeds with a pattern reminiscent of the original Unet architecture, featuring a series of concatenation and upsampling operations. This approach allows the model to gradually reconstruct the output, iteratively refining its predictions until the desired output resolution is attained.

In contrast to TransUnet, SegFormer offers a lightweight approach for semantic segmentation by leveraging transformer layers as the primary building blocks while incorporating convolutional operations in a minimal yet effective way. The model introduces several key architectural innovations in both its encoder and decoder components:

- In the encoder, SegFormer adopts a hierarchical structure to generate multi-scale feature embeddings, addressing the limitations of traditional ViT architectures that rely on single-scale low-resolution outputs.
- The decoder component of SegFormer is engineered for lightweight efficiency, utilizing MLPs (Multilayer Perceptrons) in conjunction with reshaping and upsampling operations.
- To handle the resolution discrepancies between training and inference images, SegFormer employs a position-encoding-free approach, avoiding explicit positional encodings that can lead to accuracy degradation.
- The Mix-FFN (Feed-Forward Network) module plays a crucial role in achieving implicit positional encoding. It integrates 3 × 3 convolution operations into the MLP block to effectively encode spatial information. This position-encoding free approach, aims to tackle the loss of accuracy arising from resolution discrepancies between training and inference images, commonly observed in prototypical ViT models.

The Mix-FFN module is mathematically formulated as:

(1) $$x_{out}=MLP(GELU(Conv_{3\times 3}(MLP(x_{in}))))+x_{in}$$

where:

- $x_{\text{in}}$: Input feature map to the Mix-FFN module.
- MLP: Multilayer Perceptron, responsible for feature transformation.
- GELU: Gaussian Error Linear Unit, an activation function that smooths the output of the convolution layer.
- Conv_3×3_: A 3 × 3 convolutional layer that captures local spatial patterns within the input feature map.
- $x_{\text{out}}$: Final output feature map of the Mix-FFN module after applying the transformations.

### 3.3. GAN models

Although GANs were initially introduced to boost the size of the training set with realistic samples, they have found utility in a wide variety of tasks, such as in image-to-image translation, image-to-text generation, image blending, image super resolution, etc [25,38]. Within the GAN paradigm, image segmentation falls under the category of image-to-image translation. The segmentation map (or mask) is viewed as a translation (or transformation) of the input image. In the latter half of the last decade, research groups from all around the globe have proposed a wide variety of models towards this end. We review some of the GAN models which are not only popular but also fundamental in this domain.

#### 3.3.1. AC-GAN

In traditional neural networks, training involves gradient computation and parameter updates until some convergence conditions are met. By contrast, GANs are trained not for convergence, instead they run until an equilibrium is achieved between the Generator and Discriminator. The mode collapse phenomenon observed in GANs leads to generation of globally incoherent samples, especially when there are a high number of output classes. AC-GAN proposed in [74] addresses this issue by forcing the discriminator to output the probability distribution over the classes, in addition to predicting the source of the image. This formulation requires a specialized cost function to account for the functional changes to the discriminator. The generator is conditioned by feeding in the class label in addition to noise. The log likelihood of the class distribution and source distribution are given by,

(2) $$L_{s}=E[log\;P(S=real|X_{real})]+E[log\;P(S=fake|X_{fake})]\;L_{c}=E[log\;P(C=c|X_{real})]+E[log\;P(C=c|X_{fake})].$$

where:

- $L_{s}$: Source loss, measuring how accurately the discriminator predicts whether an image is real or fake.
- $L_{c}$: Class loss, measuring how accurately the discriminator predicts the class label of an image.
- S: Source label, where S = real represents real images and S = fake represents generated images.
- C: Class label, representing the assigned class of an image.
- P: Probability function.
- $X_{\text{real}}$: Real image input to the discriminator.
- $X_{\text{fake}}$: Fake (generated) image input to the discriminator.
- E[⋅]: Expectation operator, averaging the values over a probability distribution.

The discriminator maximizes $L_{s}+L_{c}$, while the generator maximizes $L_{c}-L_{s}$. In AC-GAN, the discriminator has two tasks: to identify whether an image is real or fake (source prediction, $L_{s}$) and to predict the class label of the image (class prediction, $L_{c}$). Therefore, it must maximize the sum of these two losses. On the other hand, the generator aims to fool the discriminator into classifying fake images as real (minimizing $L_{s}$) while ensuring the generated images are accurately classified into the intended class (maximizing $L_{c}$). Hence, the loss is formulated as maximizing the difference of these losses. This adversarial setup pushes the generator to create images that are both visually realistic and class-consistent, balancing between fooling the discriminator and adhering to the target class characteristics.

#### 3.3.2. Pix2pix

**Fig. 8.**
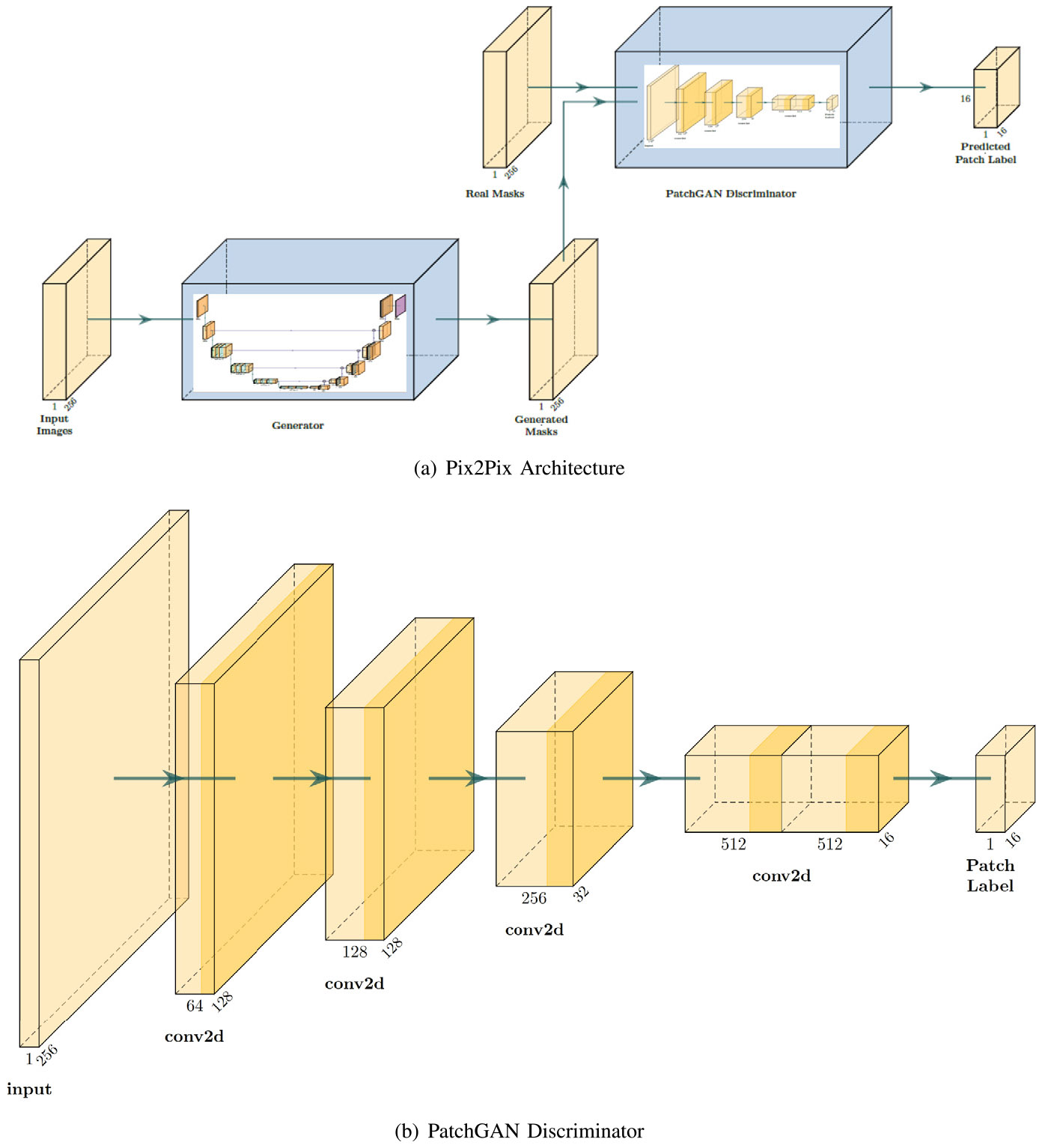
Pix2pix architecture and PatchGAN discriminator network.

Isola et al. [40] developed a generic image-to-image translation framework that learns mapping between two image domains, as shown in Fig. 8(a). The generator in this case is conditioned by the image from the input domain and is expected to translate it to the output domain. For example, for segmentation task the generator accepts the original image as input and outputs a segmentation mask. As before, the job of the discriminator is to output a probability distribution over the sources. The key to forcing the generator to produce realistic image translations is the addition of L1 loss to the objective function given by,

(3) $${ℒ}_{L1}(G)=E_{x,y,z}[{\left\|y-G(x,z)\right\|}_{1}],\;\;G^{*}=arg\;\min_{G}\;\max_{D}\;{ℒ}_{cGAN}(G,D)+\lambda {ℒ}_{L1}(G).$$

where:

- ${ℒ}_{L1}(G)$: L1 loss, measuring the pixel-wise difference between the generated image and the target image using the L1 norm.
- E[⋅]: Expectation operator, averaging the values over a probability distribution.
- x: Input image from the source domain.
- y: Target image from the destination domain.
- z: Random noise vector or latent code fed into the generator.
- G: Generator network, responsible for translating input x into output y.
- $G^{*}$: Optimal generator minimizing the objective function.
- D: Discriminator network, responsible for distinguishing real images from generated ones.
- ${ℒ}_{cGAN}(G,D)$: Conditional GAN loss, ensuring the generator produces outputs that are indistinguishable from real images when conditioned on input x.
- λ: Weighting factor that balances the contribution of the L1 loss relative to the adversarial loss.
- $\arg\;\min_{G}$: Represents the optimization of the generator parameters to minimize the loss.
- $\max_{D}$: Represents the optimization of the discriminator parameters to maximize the adversarial loss.

The addition of L1 or L2 loss to the objective function forces faithful reproduction of low frequency structure in the translated image. For correctly incorporating high frequency structure [40] proposed the PatchGAN discriminator. Rather than the discriminator producing a single verdict of real or fake label for the entire image the discriminator classifies each N×N patch in the image as real or fake. This ensures that the generated images have sufficient high frequency detail across the entire image. Fig. 8(b) shows the patchGAN architecture. The input masks undergo multiple strided convolutions so that the spatial dimension is reduced from 256 × 256 to 16 × 16. For the first time in the cell segmentation literature, we incorporate HRNet, Mobilenet-UNet, ResNet50-UNet as generators into the pix2pix framework and test it for HEp-2 cell segmentation. Recently, diffusion models [27,28] are advocated for generative modeling of radiological images and these models can be adapted to obtain cell segmentations.

## 4. Experiments and results

### 4.1. Datasets description

The MIVIA dataset^5^ was curated and utilized in 2010 study for HEp-2 mitotic (63 cell images) vs non-mitotic (63 cell images) cell classification [85] submitted to the 2010 IEEE 23rd International Symposium on Computer-Based Medical Systems (CBMS). The authors used five classifiers with the highest accuracy of 86.51% for k-nearest neighbor approach. Following this study, in 2012, the MIVIA dataset was publicly introduced in the International Conference on Pattern Recognition (ICPR) 2012 in Japan, for the HEp-2 Cell Classification contest — 1457 images (14 instances/723 cell images in training, 14 instances/734 cell images in testing) belonging to 6 cell classes — homogeneous, centromere, speckled, nucleolar, mitotic spindle, and Golgi. This contest received 28 benchmarking methods [2], varying across different machine learning techniques — kNN, SVM, logistic regression, etc. The I3A, a larger HEp-2 dataset included 13,596 cell images across 6 cell patterns, from 83 specimens, and was part of cell classification at the 20th IEEE International Conference on Image Processing (ICIP) 2013 in Australia [86]. They received 14 submissions from all across the world. ICPR 2014 (Sweden) continued with the same dataset for HEp-2 cell contest with additional tasks of segmentation and mitotic cell recognition. In ICPR 2016 (Mexico) contest, the dataset was supplemented with a Python-based software tool for computing the performance indices on training dataset and fine tuning algorithms.

### 4.1.1. I3 A dataset

This dataset was introduced in a contest in ICIP 2013 and later re-proposed in ICPR 2014 and ICPR 2016. This dataset is an indirect immunofluorescent image-based dataset. It contains 252 specimens which belong to seven different categories. The class distribution is as follows: 53 Homogeneous, 52 Speckled, 50 Nucleolar, 51 Centromere, 10 Golgi, 21 Nuclear Membrane, and 15 Mitotic Spindle. For each of the 252 specimens, 4 images were captured, each of size 1388 × 1040. Hence, the total number of images in the dataset is 1008. All the images are in grayscale and the dataset was split into 555/151/302 images for training, validation and testing purpose. The segmentation task on this dataset is a binary segmentation problem, to differentiate the foreground from the background, specially since each image only contains cells from one of the seven cell stain patterns. The ground truth masks are not manually annotated, but rather taken from the DAPI channel and hence the ground truth is considered silver-standard. We train the models by extracting random patches from the images. It must be noted that the images in this dataset contain only one staining pattern per image. This consistency of staining pattern across an image ensures that original class distributions are conserved even if the patch extraction process is random.

In order to train the models, K random patches of size 256 × 256 are extracted from each training image. To determine the optimal K we trained a UNet model by extracting [4, 7, 12, 20, 30] patches per image. The performance of the models on the test set are shown in Table 2. While there is a noticeable drop in performance when using 4 patches/image for the other cases of 7, 12, 20 and 30 the differences in performance across patch counts are not significant. This is primarily because the staining pattern within each image is the same. Hence, increasing the number of patches does not necessarily provide additional useful information for training the model.

For testing, ordered patches are extracted with a stride of 70 pixels and final segmentation map is obtained by averaging the result from overlapping patches. The primary evaluation metric for comparing model performance is the Dice coefficient, which effectively measures segmentation overlap. Additional metrics, including Accuracy, Precision, Sensitivity, Specificity, AU-PR, AU-ROC, and IoU (Jaccard Index), provide complementary insights into the model’s performance.

### 4.2. Experiments

#### 4.2.1. Parameter setup

To extract the best performance for every model on the I3A dataset we setup our experiment as follows. Every model is trained for 25 epochs with a batch size of 64. Since, our aim is to benchmark the performance of deep learning models we refrain from performing any pre or post processing operations to the input and outputs images of the model. We systematically swept the base learning rate from 10^−3^ to 10^−6^ by a factor of 0.1. We utilized the Adam Optimizer and retained the default values for the *β*1 (0.9) and *β*2 (0.999) decay parameters. Given the relatively small training set we took additional steps to prevent model overfitting by utilizing early stopping and reducing learning rate on plateau. We monitored the validation loss and implemented early stopping with a patience of 7 epochs with an epsilon of 10^−3^. We also monitored the validation accuracy and reduced the learning rate by a factor of 0.1 when a plateau is detected for 4 epochs.

#### 4.2.2. Without pretraining

In our first experiment, we trained each model from scratch, i.e., without using pre-trained encoders. For FCN32, we considered only the vanilla architecture without any pre-trained encoders. For FCN8, SegNet, and U-Net, we included all three encoder architectures (VGG16, ResNet50, MobileNet) in addition to the vanilla model. However, for PSPNet, we evaluated only the VGG16 and ResNet50 encoders, while for HRNet, the concept of pretrained encoders is not applicable. In addition to the architectures described in Section 3.1, we benchmarked Cellpose [39] on the I3A dataset test set (302 images). Table 3 presents the performance of the models evaluated on the test images (302 images). The FCN8 vanilla model outperformed FCN32 by 0.07 points in the Dice metric, underscoring the importance of higher spatial resolution feature descriptors in generating accurate segmentation maps. Within the FCN8 family, the VGG, ResNet50, and MobileNet encoders consistently outperformed the vanilla encoder by an average of 0.02 points. Interestingly, PSPNet and its variants exhibited lower performance compared to FCN8 variants, suggesting potential limitations in their architectural design for this dataset. Unexpectedly, while the Dice scores of SegNet, VGG-SegNet, and MobileNet-SegNet were significantly lower than FCN32, the ResNet50-SegNet model achieved an impressive Dice score of 0.860. The performance of U-Net variants (VGG-UNet, ResNet50-UNet, MobileNet-UNet) was comparable to FCN8 variants. Among all models, HRNet recorded the best performance, achieving a Dice score of 0.901. Transformer-based models such as TransUnet, DeepLabV3+, and SegFormer demonstrated competitive performance, despite their higher parameter counts (100M for TransUnet, 47M for SegFormer) and smaller datasets. Specifically, DeepLabV3+, despite having only 11M parameters, achieved a higher Dice score (0.84). Cellpose managed to achieve a Dice score of 0.693 on the test set.

#### 4.2.3. Pretrained models

We wanted to study the impact of model pretraining on I3A dataset. To this end, we initialized the models with ImageNet pretrained weights for the encoders and fine-tuned them in two different modes. In the first mode, we froze the encoder weights and allowing only the decoder weights to be modified and in the second case we allowed both the encoder and decoder weights to be learned during training. The performance of the models on the test set is documented in Table 3. The TransUnet model, equipped with a pretrained encoder, displayed a remarkable performance, achieving an impressive dice score of 0.921.

#### 4.2.4. Cross validation and statistical significance of model performance

To evaluate the statistical significance of the observed model performances, we conducted a 5-fold cross-validation for each model. Table 4 presents the performance metrics across the five folds, along with the mean and standard deviation. To determine whether the differences between model performances are statistically significant, we performed paired t-tests across the folds for all pairwise model comparisons. The resulting p-values were adjusted using the Benjamini–Hochberg (BH) correction to control the False Discovery Rate (FDR), ensuring that the proportion of false positives among significant results remains bounded. Comparisons with corrected p-values below the significance threshold were considered to have meaningful performance differences, ensuring robust statistical conclusions. Table 5 displays the BH-corrected p-values, highlighting model pairs where performance differences are statistically meaningful. We identified 81 statistically significant model pairs (*P* < 0.005) based on the BH correction. The results indicate that model architectural choices and pretraining strategies play a significant role in determining segmentation performance.

#### 4.2.5. Analyzing classwise performance of models

Since the images of the I3A dataset belong to one of seven distinct staining cell patterns, we evaluated the performance of all deep CNN models across these classes. Table 6 presents the class-wise performance of the models. The columns in the table are roughly arranged in a least-to-most performant fashion—traversing a row from left to right reveals a noticeable decline in performance across most models. We observed not only a decline in performance across all models but also a sharp increase in variance among models within each class. This variance suggests the relative difficulty of learning the characteristic features of specific classes. The Mitotic Spindle and Golgi classes exhibited the poorest performance across all models. Upon closer examination, we identified three key contributing factors for this observation:

1. Limited Sample Size: The Golgi class contains only 10 samples and the Mitotic Spindle class contains 15 samples, which limits the model’s ability to generalize effectively.
2. Image Quality: Images in these two classes are often qualitatively poor, with low contrast, and exhibit complex features that are challenging to learn.
3. Ground Truth Quality: Some ground truth masks for these classes are suboptimal, which adds noise to the training process (as further demonstrated in Section 4.3).

The results documented in Table 3 are deemed our baseline, owing to their broad representation of models from diverse families. For subsequent experiments, we performed augmentation and domain specific pretraining experiments on the Top-4, Mid-3 and Bottom-3 models, with the goal of analyzing the effect of these on the model performance. The Top-4 models were selected based on their Dice scores from the baseline evaluation in the previous section, representing the best-performing architectures. Conversely, the Bottom-3 models were the models that achieved the least dice scores in the baseline evaluation. The Mid-3 models were chosen as 3 almost equally spaced models between the Top-4 and Bottom-3 cases to ensure a fair representation in our analysis. For the GAN pix2pix framework we considered only the Top-4 models only, primarily because it is well known that GAN models tend to be harder to train and suffer from mode collapse.

#### 4.2.6. Effect of domain specific PreTraining

The transfer learning paradigm in deep learning involves initializing weights learned from the ImageNet dataset and fine-tuning across different domains. This approach works reasonably well in practice as generating faithful encodings of the natural world using millions of varied images, imparts the model with a rich and diverse feature set. Within the deep learning community, it is widely recognized that domain-specific pretraining consistently exhibits superior performance compared to non-domain pretraining approaches, rendering it a compelling and highly promising avenue for further comprehensive investigation and research. The availability of the I3A Task-1 dataset presents a unique opportunity to delve deeper into domain-specific pretraining methods and its potential benefits. Specifically, the I3A Task-1 dataset contains 13596 single cell images extracted from 83 specimens belonging to six different cell staining patterns viz. Homogeneous (2494), Centromere (2741), Speckled(2831), Nuclear Membrane (2208), Nucleoloar (2598) and Golgi (724). The Mitotic Spindle class is not featured in this dataset.

Despite being a cell type classification task, the I3A Task-1 dataset includes silhouette masks for cell segmentation. In this experimental study, we carried out a transfer learning as follows. We trained a segmentation model, initialized with random weights, for the auxillary task of segmenting single cell images using I3A Task-1 data. Subsequently, while training models for Task-2 we utilize the Task-1 pretrained weights for initialization and fine-tuned them on the Task-2 dataset for 25 epochs with the same parameters mentioned in Section 4.2.1. Unlike the I3A Task-2 dataset, the image sizes of this dataset are not fixed and vary from 54 × 67 to 111 × 108 pixels with a mean of 68.70 and standard deviation of 6.32. We homogenize the size of all images by resizing the whole dataset to 64 × 64. The dataset was split into Train/Val/Test with the ratio 60/10/30. We trained the Bottom-3, Mid-3 and Top-4 models with this dataset for 20 epochs, with batch size 64 and a learning rate of 0.001 using the Adam optimizer. The performance of the models on this dataset is shown in Table 7. All the models exhibit comparable performance on this dataset and obtained dice scores in the 0.90 to 0.96 range with HRNet showing the best performance. The consistency of their performance underscores the relative simplicity of this task and the effectiveness of these models on this task. Using these as the pretrained weights, we fine-tuned them on the challenging Task-2 dataset using the same hyperparameters as before. For the Top-4 models, domain-specific pretraining (DSPT) enhances the performance of HRNet and ResNet50-UNet on Task-2 as demonstrated in Table 9. In contrast, MobileNet-UNet* and TransUNet* exhibit a notable decline in performance which can be attributed to overfitting. Upon closer examination of HRNet, it becomes apparent that while there is a marginal improvement in overall performance, this enhancement is accompanied by a decline in the performance of the Golgi class, refer Table 12. For the Bottom-3 models, the DSPT pretraining proved beneficial for all 3 models. For the Mid-3 models, VGG-UNet* saw a drastic decline in performance while the other 2 models saw marginal improvements. The classwise results for the Bottom-3 and Mid-3 models are shown in Tables 10 and 11 respectively.

#### 4.2.7. Effect of augmentation

As stated in Section 4.2.3, due to the limited representation of the Golgi and Mitotic Spindle classes in the I3A Task-2 dataset, we adopt data augmentation techniques to address the deficiency in data quantity and quality. Two distinct data augmentation strategies are pursued, and their respective performances are compared. In both cases, we employ RandAugment [87] with the augmentations specified in Table 8. Specifically, we randomly select three out of the eight possible augmentation techniques and apply them sequentially to each image in the dataset.

##### 4.2.7.1. Augmentation strategy - 1.

This is a minority boosting strategy in which we apply data augmentation only to the Golgi and Mitotic Spindle class and apply a balanced sampling between original and augmented datasets to boost the representation of these two classes. For each image in the Golgi and Mitotic Spindle class, five augmented images are generated. Each augmentation is generated by randomly selecting 3 of the 8 possible augmentation techniques and applying them sequentially on the image. Using this approach we trained the Bottom-3, Mid-3 and Top-4 models. In this training scheme, half the samples from each batch are taken from the original dataset and half are obtained from the augmented data. This procedure ensures that a higher proportion of samples from Golgi and Mitotic Spindle class are presented to the model during training. We reduced the base learning rate to 0.0001 for this task while keeping all other hyperparameters constant. An inspection of the results shown in Table 9 (column DA-1) reveals that the Dice coefficients of HRNet and ResNet50-UNet did not show any significant improvement while that of MobileNet-UNet with pretrained encoder had reduced drastically. The performance of TransUNet model also saw a notable decrease compared to its baseline performance. Among the Bottom-3 models, MobileNet-SegNet saw a notable improvement in dice while the performance of VGG-PSPNet* was degraded compared to the baseline. For the Mid-3 models, FCN8-MobileNet* saw a decline in performance as did the performance of FCN8.

##### 4.2.7.2. Augmentation strategy - 2.

In this approach, data augmentation was applied uniformly across all training images in the I3A Task-2 dataset. For each class, 500 augmented images were generated, resulting in a balanced dataset with 3500 images evenly distributed across the seven classes. This process was repeated five times, creating five distinct augmented datasets. Each dataset was used to train the Bottom-3, Mid-3, and Top-4 models, which were then evaluated on the I3A Task-2 test set.

Despite the inherent randomness of the augmentation process, model performance exhibited minimal variance across the five datasets. As shown in Table 9, ResNet50-UNet and MobileNet-UNet experienced a slight decline in Dice scores compared to their baseline performance. In contrast, TransUNet* suffered a significant drop of approximately 10 points in Dice, particularly affecting the Mitotic Spindle and Golgi classes. This augmentation strategy resulted in improved performance for all models in Bottom-3 category while for the Mid-3, FCN8 suffered a decrease in performance while the other two models saw significant improvement in the test dice scores.

HRNet demonstrated consistent performance, showing robustness across different augmentation strategies. This trend was similarly reflected in class-wise evaluations, where HRNet maintained stability while MobileNet-UNet and ResNet50-UNet exhibited slight performance drops across all classes, as shown in Table 12. TransUNet* displayed a broader decline across all classes, with the Mitotic Spindle and Golgi classes being the most affected. Class-wise performances of the Bottom-3 and Mid-3 models are detailed in Tables 10 and 11, respectively. These results highlight the varying sensitivity of different model architectures to global augmentation strategies.

#### 4.2.8. GAN

The principal motivation for considering GANs in this study was to explore the efficacy of adversarial training frameworks, such as Pix2pix, for a binary segmentation problem with limited annotated samples. GANs have been successfully employed in medical image analysis to improve segmentation outcomes by leveraging their ability to generate realistic image mappings. In particular, the Pix2pix framework has been shown to enhance performance in tasks where paired image-to-image translation can assist in refining segmentation boundaries. This motivated us to investigate whether adversarial training could provide meaningful improvements on the HEp-2 cell segmentation task, especially for challenging classes like Golgi and Mitotic Spindle, which suffer from severe data scarcity and poor ground-truth quality.

In this experiment, we employed the top four performing models (HRNet, ResNet50-UNet, MobileNet-UNet*, and TransUNet*) as generators within the Pix2pix framework. A convolutional PatchGAN discriminator was implemented to classify image patches as real or fake. The discriminator consisted of a six-layer convolutional network, where the spatial dimensions of the inbound layer were halved and the channel dimensions doubled at every layer. The final output was a 16 × 16 map with one channel, generated by a 2D convolutional layer with a 4 × 4 kernel. The models were trained for 25 epochs with a base learning rate of 10^−4^, and all other hyperparameters were kept consistent with previous experiments.

While increasing the number of patches per image might seem like a viable solution to address data scarcity, our initial patch analysis experiment (Section 4.1.1) revealed diminishing returns beyond a certain threshold. We evaluated multiple patch extraction strategies, including extracting 4, 7, 12, 20, and 30 patches per image. Although extracting only 4 patches resulted in a notable drop in segmentation performance, increasing the patch count beyond 7 did not yield significant improvements in the Dice or IoU metrics (as shown in Table 2). This outcome suggests that the uniform staining patterns within each image limit the diversity of useful features captured by additional patches. While data augmentation strategies (as explored in Augmentation Strategies 1 and 2) could have been incorporated alongside the GAN training framework, our objective was to assess the raw performance of adversarial training without introducing confounding effects from augmented datasets. This approach ensures that the observed results are a direct reflection of the impact of the GAN-based training methodology, rather than being influenced by augmentation artifacts.

As expected, the GAN-based models demonstrated a decline in performance compared to their standalone segmentation counterparts, primarily due to the limited training samples and the intrinsic challenges associated with adversarial training stability. Table 9 presents a comparative overview of the Dice and IoU scores achieved under the GAN framework versus other conditions. Class-wise analysis (Table 12) revealed interesting patterns. MobileNet-UNet: Performance declined significantly for the Mitotic Spindle and Golgi classes but outperformed augmentation-based strategies for the other five classes. HRNet and ResNet50-UNet, similar patterns emerged, with slight degradations in challenging classes but competitive performance otherwise. TransUNet* exhibited the sharpest decline across all classes, particularly in the Golgi class.

### 4.3. Segmentation results

**Fig. 9.**
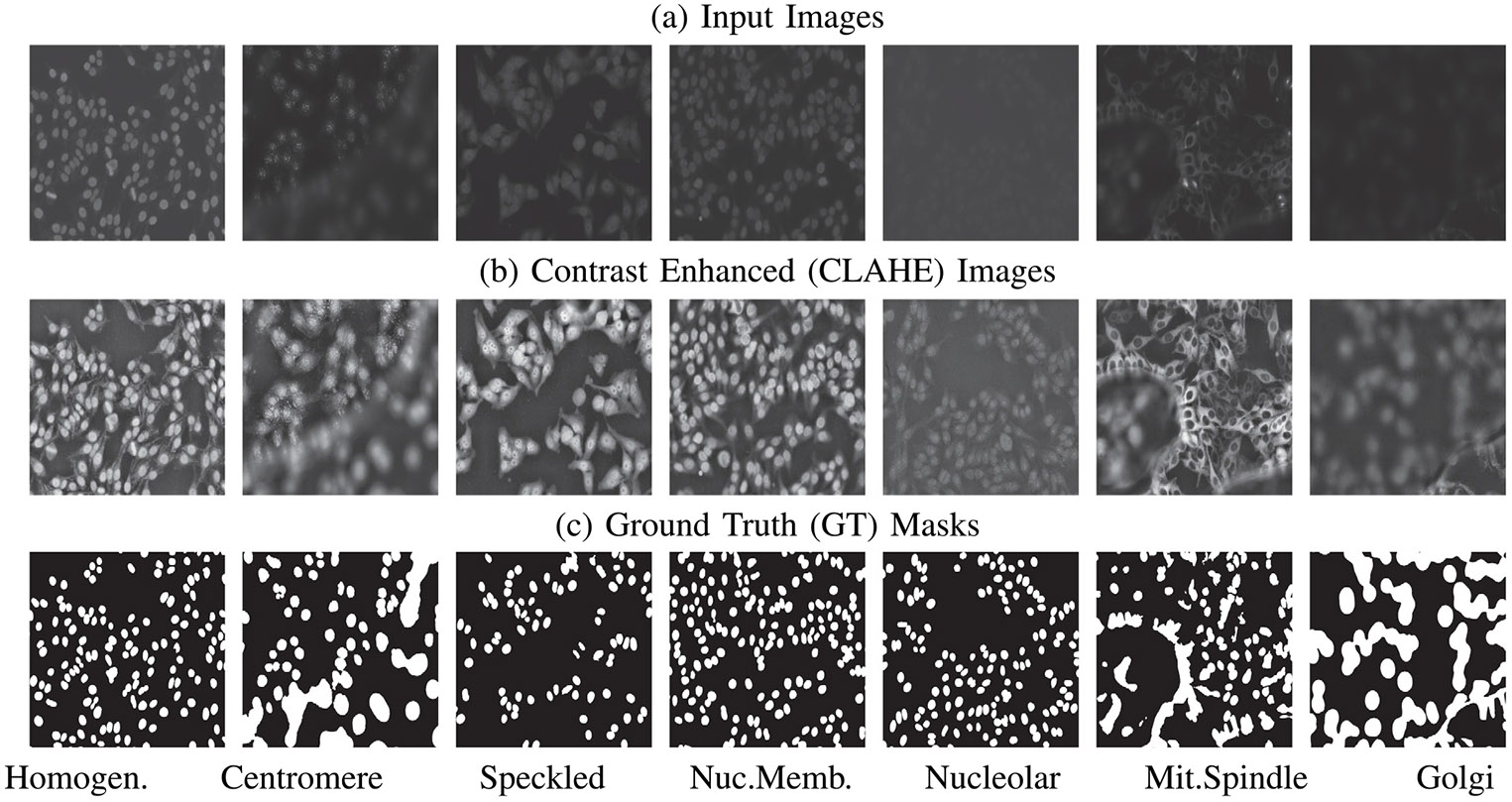
Visualization of selected images and ground truth masks from I3A dataset.

A visual analysis of the input images, ground truth and the predicted masks of different models is particularly helpful for semantic segmentation tasks. A visual exploration highlights the generalizability of deep learning models and sheds light on the challenges in the data. The images in this dataset suffer from a lack of contrast and this is immediately apparent in Fig. 9. Among the seven staining patterns depicted in this dataset, the Homogeneous class stands out for its consistently superior contrast compared to other classes. A closer inspection of Fig. 9 indicates that, even for the Homogeneous class, the raw image lacks perceptible texture in both the cell and its surroundings compared to its contrast enhanced version. On the other hand, the Nucleolar, Mitotic Spindle and Golgi stains pose significant challenges, as even contrast-enhanced versions of their images remain visually indiscernible. The segmentation masks for the images in the I3A dataset the ground truth were obtained directly from the DAPI channel rather than manual annotation which has resulted in poor ground truth for many images particularly in the Golgi and Mitotic Spindle class. This unfaithful nature of the ground truth masks is evident in Fig. 9. For the interested reader, we have provided visualization of additional samples along with their Dice scores in the Supplementary.

**Fig. 10.**
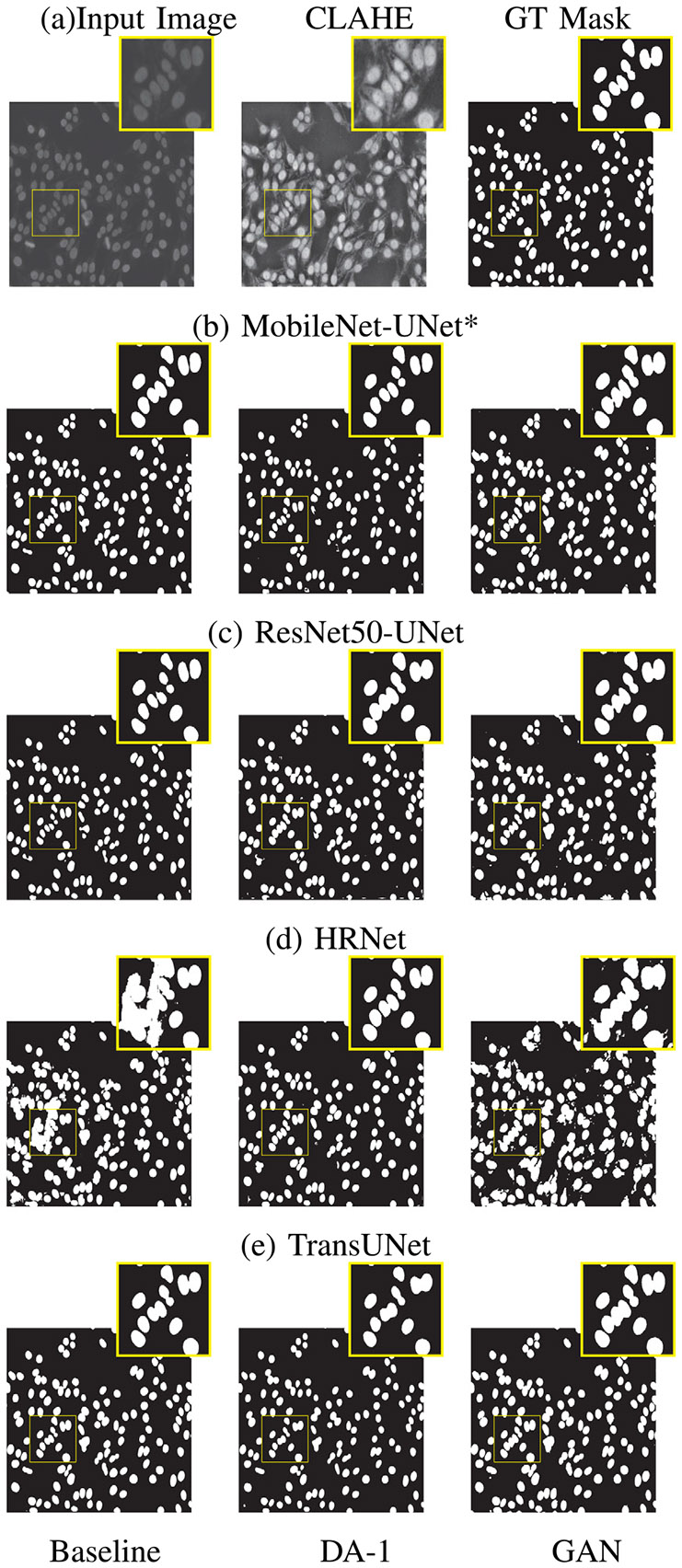
Visulalization of select segmentation maps for Homogeneous class obtained from the 4 models under different conditions.

**Fig. 11.**
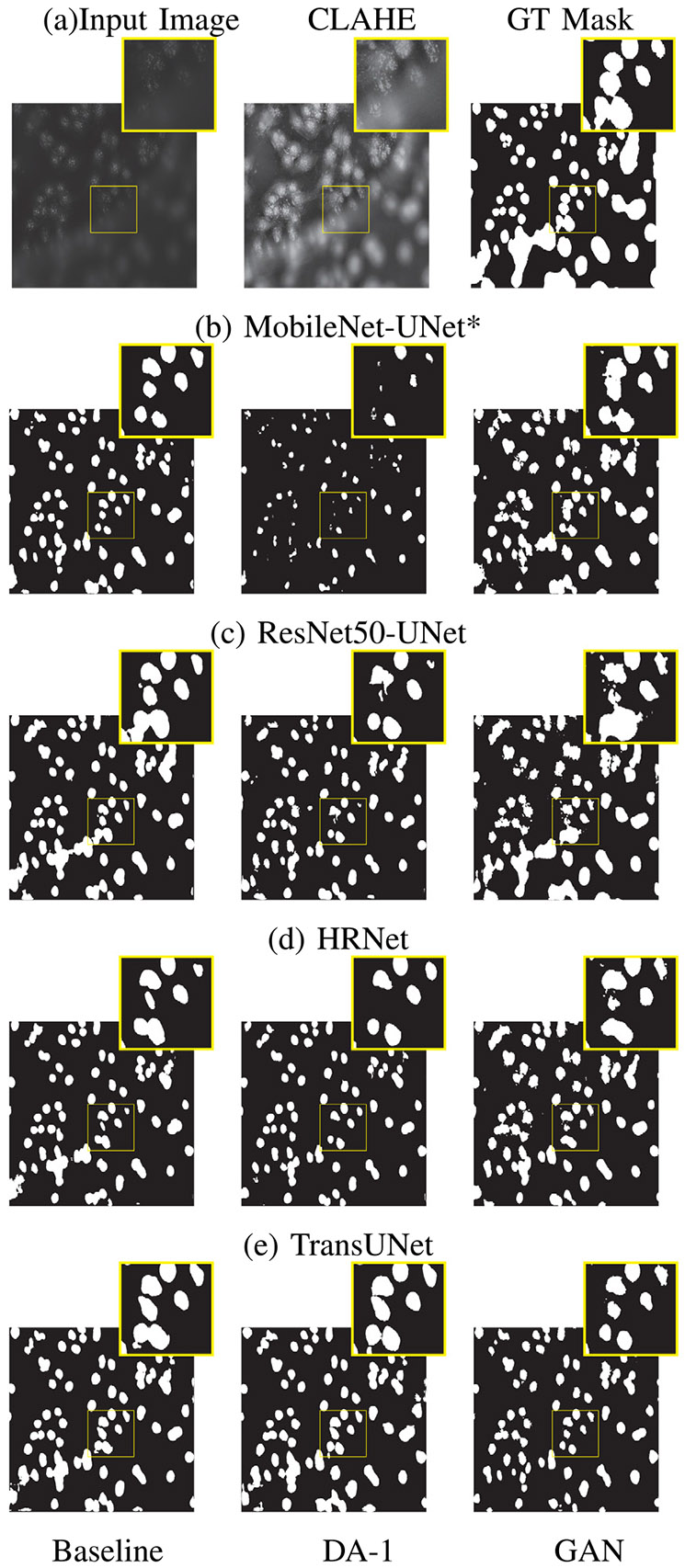
Visulalization of select segmentation maps for Centromere class obtained from the 4 models under different conditions.

**Fig. 12.**
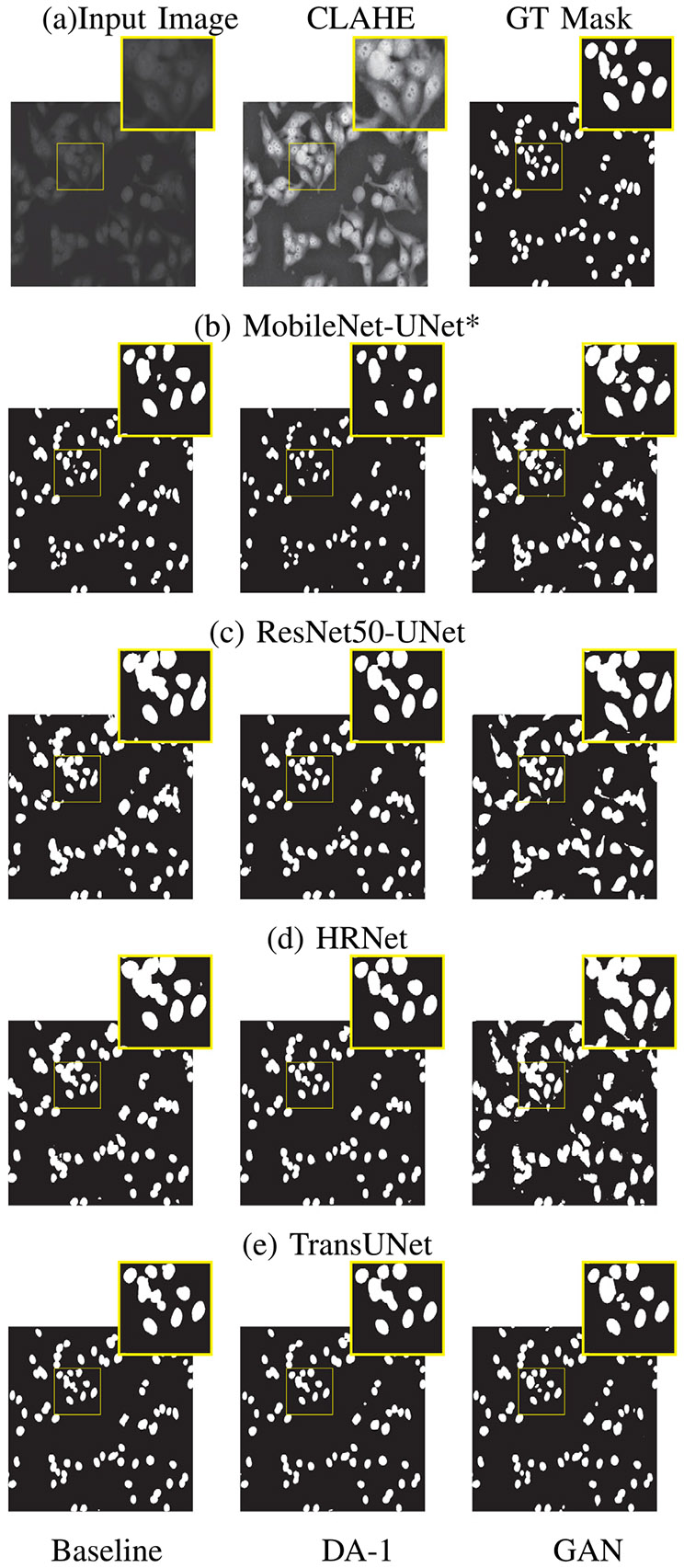
Visulalization of select segmentation maps for Speckled class obtained from the 4 models under different conditions.

**Fig. 13.**
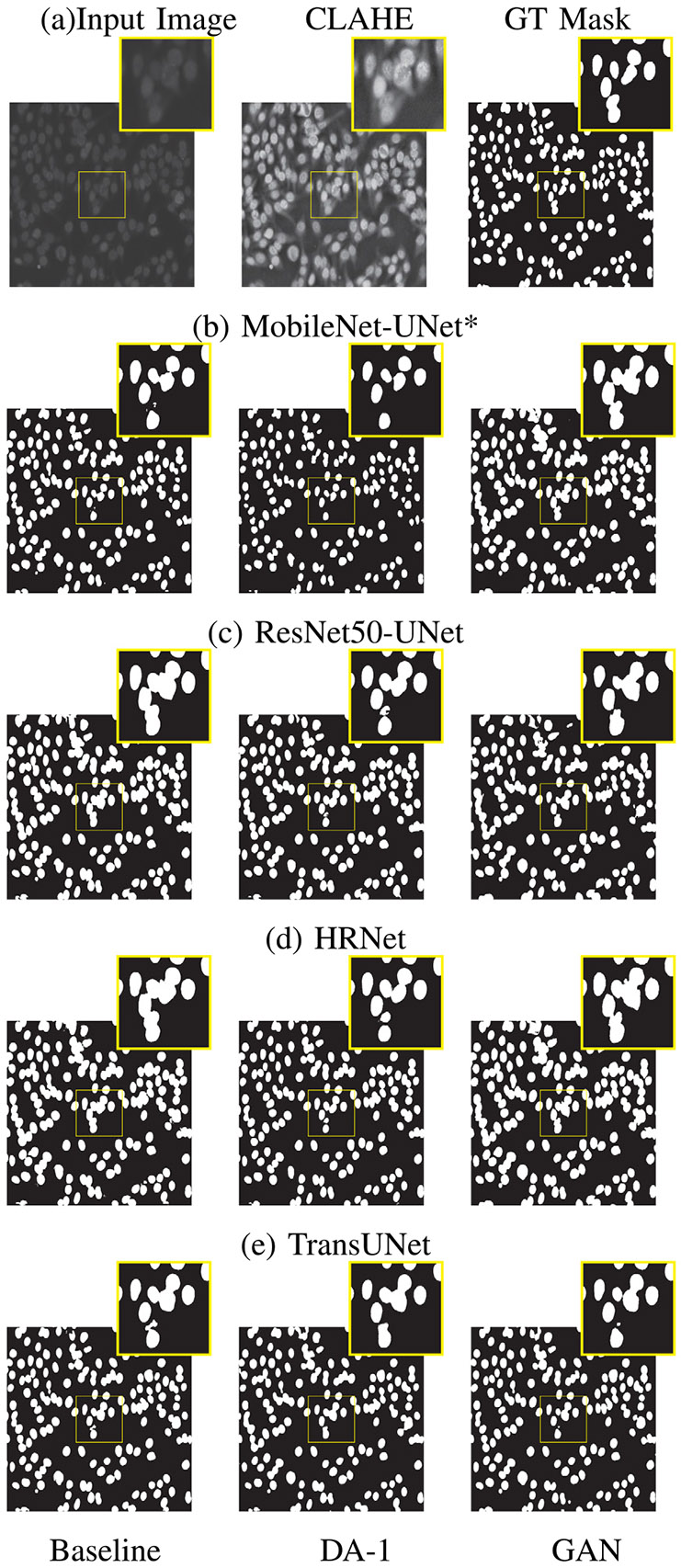
Visulalization of select segmentation maps for Nuclear Membrane class obtained from the 4 models under different conditions.

**Fig. 14.**
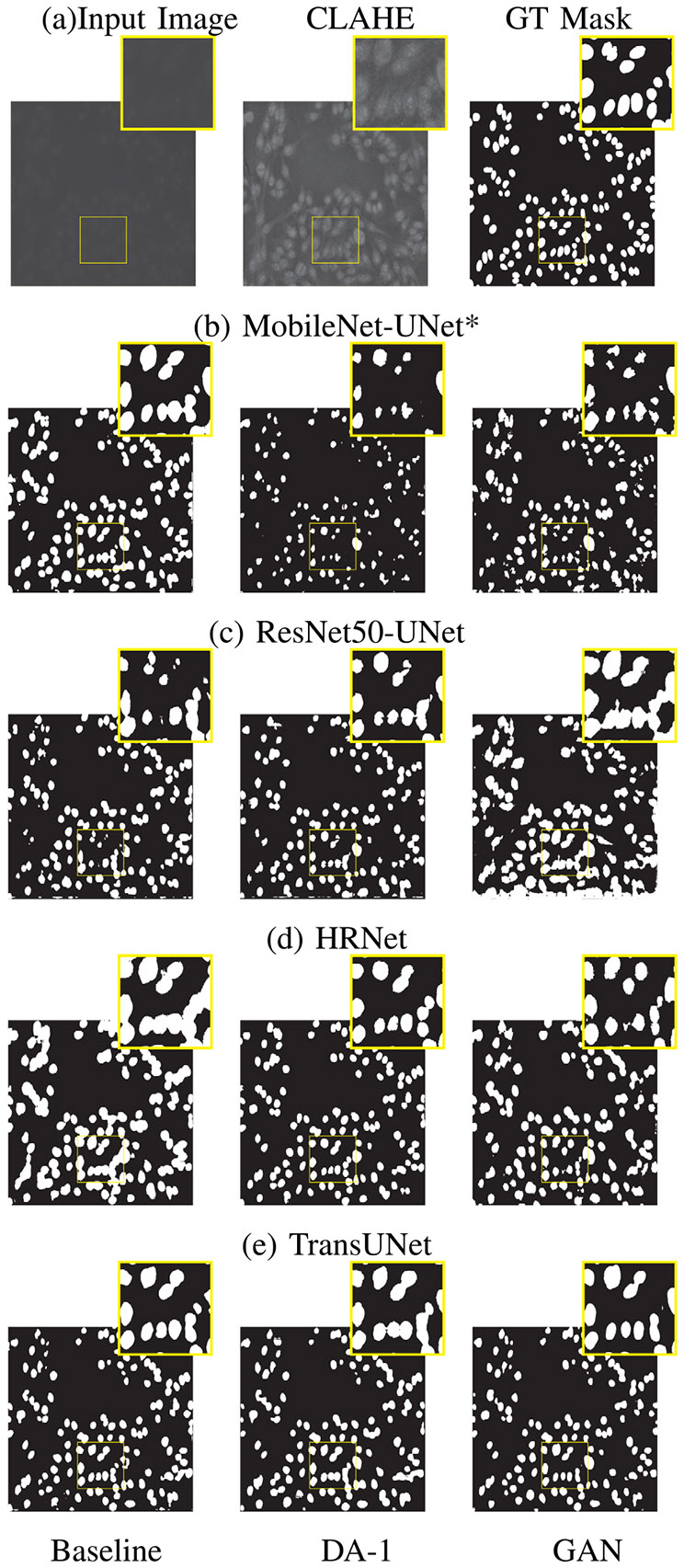
Visulalization of select segmentation maps for Nucleolar class obtained from the 4 models under different conditions.

**Fig. 15.**
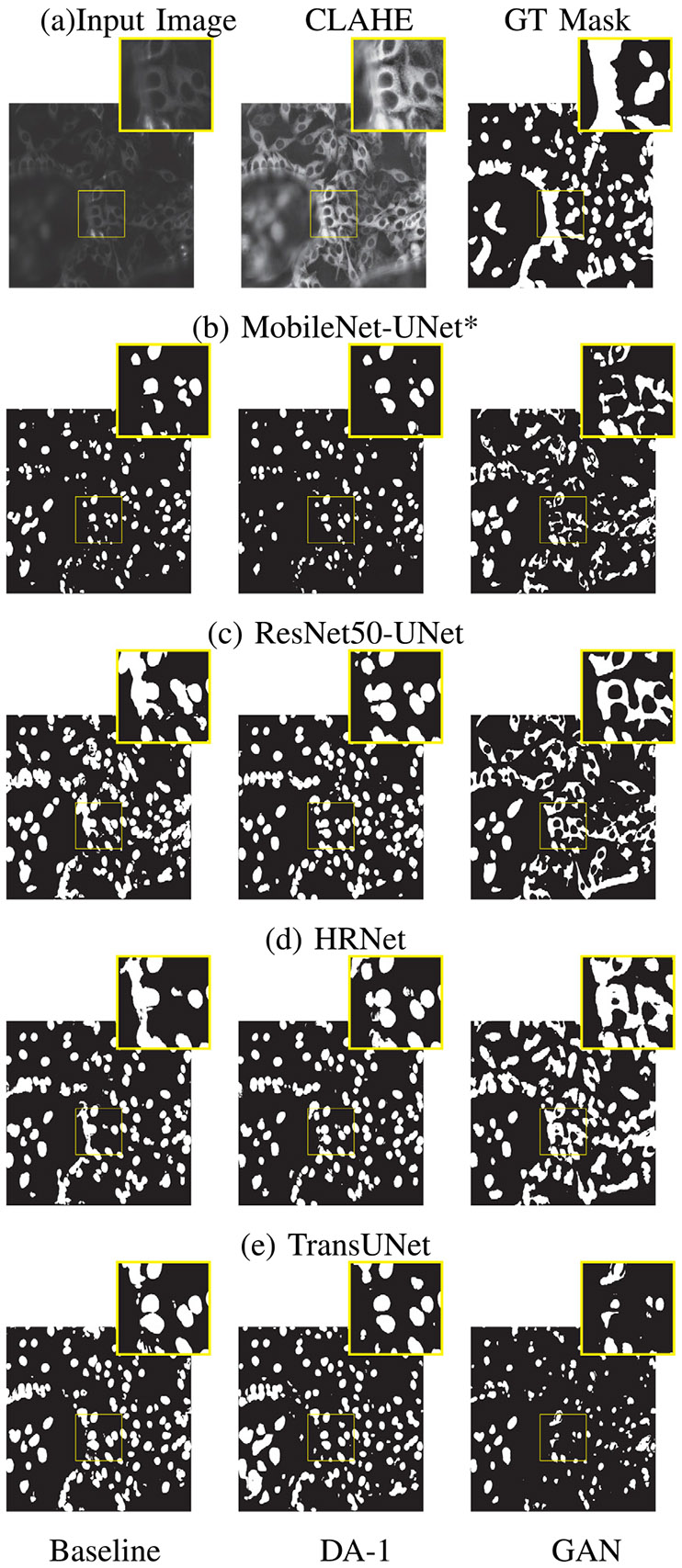
Visulalization of select segmentation maps for Mitotic Spindle class obtained from the 4 models under different conditions.

**Fig. 16.**
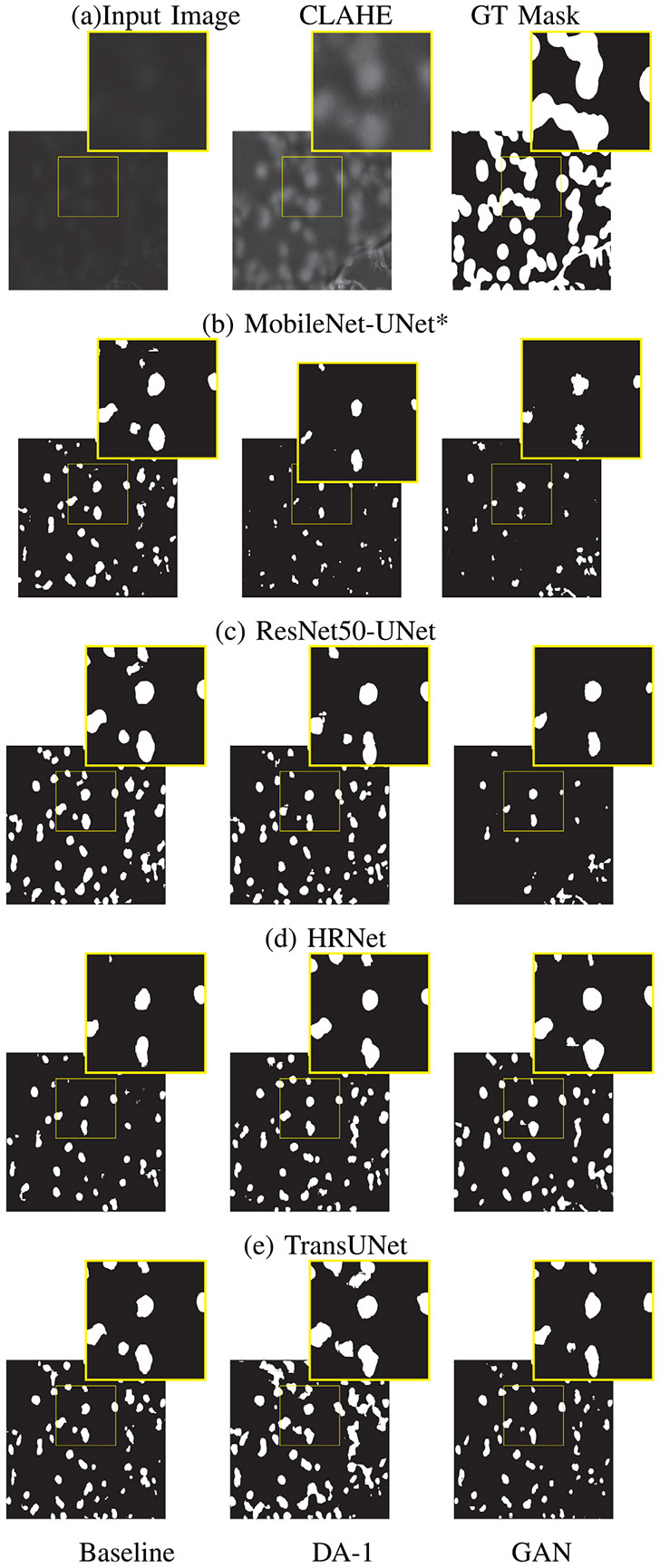
Visulalization of select segmentation maps for Golgi class obtained from the 4 models under different conditions.

We visualized the segmentation maps produced by the top models under various conditions. However, here we have restricted our visualization to only 1 sample image from each class. For the Homogeneous class we can observe from Fig. 10 that the ground truth is quite reliable and the model outputs in most cases is faithful to the ground truth mask, in particular the DA-1 augmentation scheme has enabled the models to produce maps with discernible cell like structures. A similar observation can be made for the Centromere class as well. The example we have chosen for visualization is a particularly challenging image in which a part of the image is blurry and the corresponding area in the GT mask is noisy as well, refer Fig. 11. The GAN models in this case tend to produce either blob like segmentations or misshapen cell structures. HRNet and TransUNet produced better maps in this case compared to other models. For the Speckled case, it is clear that data augmentation enabled the models to produce better segmentation maps, refer Fig. 12. We do note that the TransUNet GAN model has produced the best segmentation map among all models for this particular case. For the Nuclear Membrane and Nucleolar cases, we observe cells that are tightly grouped in the input image. As a consequence, the DL models tend to produce blob like segmentation structures in these areas as shown in Figs. 13 and 14. In both these cases, HRNet with Data Augmentation produces visually appealing segmentations compared to other models. Finally in the case of Mitotic Spindle and Golgi, the input image as well as ground truth are extremely noisy, making them the most challenging classes for segmentation. Once again we observe that HRNet and TransUNet with augmentation produces the best maps compared to other models, refer Figs. 15 and 16.

### 4.4. Perspectives and future directions

#### 4.4.1. Model advantages and disadvantages

To provide a comprehensive understanding of the strengths and limitations of different segmentation architectures, we have summarized the key advantages and disadvantages of each model type in Table 13. This comparison highlights the trade-offs in computational efficiency, accuracy, and suitability for specific segmentation tasks across various CNN and GAN architectures. We have also included the pros and cons of Domain-Specific Pretraining and the two different data augmentation strategies. Analyzing these trade-offs can guiding the selection of appropriate architectures and training paradigms depending on the data characteristics being dealt with.

#### 4.4.2. Discussion

HEp-2 cells exhibit different morphological characteristics across staining patterns and compared to the traditional image processing methods we have shown that DL-based models obtain robust segmentation results. Based on our experimental results on various CNN and GAN models we observed the following:

- We successfully trained multiple CNN and GAN models with a relatively small dataset of just 3885 images and found that the models were able to successfully segment different cell staining patterns.
- Patch Extraction Strategy and Insights: Our experiments included an analysis of the impact of varying the number of random patches extracted from each training image. We observed a diminishing return in performance when increasing the number of patches per image beyond 7, indicating that the uniform staining patterns across HEp-2 cell images limit the additional benefit of extracting more patches for model training. Future work could explore more sophisticated patch extraction strategies, such as dynamic or content-aware patch sampling, to enhance training data utility.
- It is evident that DL models learn to segment some staining patterns better than others. For example, for the Homogeneous, Centromere and Speckled classes we not only observe high mean score ($\mu_{dice}\geq 80\%$) but also low variance for all models ($\sigma_{dice}\leq 0.140\%$). This can be attributed to the fact that there are an adequate number of training images present in the I3A dataset for learning representative patterns for these classes.
- For the Nuclear Membrane and Nucleolar classes the mean Dice score across models drops (average $\mu_{dice}=72.6\%$) while the variance across models increases (average $\sigma_{dice}=0.19\%$). While the number of training images for Nuclear Membrane and Nucleolar are sufficient (84 and 200 of 1008 respectively), the images have poor contrast and the associated ground truth masks are of low quality.
- For the Mitotic Spindle and Golgi classes, the mean Dice score drops significantly ($\mu_{dice}$ 58% and 49% respectively) and the variance among models is also very high. This can be attributed to the lack of training samples (60 and 40 respectively), poor quality of the images and poor ground truth masks.
- To ensure the robustness and reliability of our results, we performed a 5-fold cross-validation across all models and evaluated their performance on the I3A Task-2 dataset. This approach reduced the impact of data split variability and provided a more comprehensive estimation of model generalization. Furthermore, we conducted paired t-tests across all pairwise model comparisons and applied the Benjamini–Hochberg (BH) correction to control the False Discovery Rate (FDR). This statistical validation revealed that 81 model pairs exhibited statistically significant differences (*p* < 0.005), confirming that the observed performance variations were not due to random chance.
- For the Mitotic Spindle and Golgi classes, the HRNet model performed significantly better (Dice score of 87% and 83% respectively) compared to all other models. However, upon deeper investigation we found that the model tends to memorize ground truth masks. This indicates the plasticity/learnability of the model.
- On a related note, although the performance of pretrained MobileNet-UNet is somewhat low for the Mitotic Spindle and Glogi classes (81% and 68% respectively), when we performed visual inspection we found the artifacts in the generated masks to be aligned better with cell like patterns even for images with poor ground truth masks. This can be attributed to the depthwise separable convolutions employed in MobileNet architecture which constrains the features/patterns learnable by the kernels and hence acts a regularization to prevent overfitting. Hence, we hypothesize that incorporating Depthwise Separable Convolutions into the HRNet lattice structure could give us the best of both worlds i.e. the plasticity of HRNet with the constrained feature learning of separable convolutions could lead to even better results for cell segmentation.
- In Section 4.3, we have showed the DL models obtained lower Dice scores using the silver-standard ground truth (GT). This is because ground truth labeling (obtained automatically using DAPI channel) is not accurate in some cases, and adversely affected the pixel-level segmentation mask scores, even though visually the DL models’ segmentation results are good. This can be mitigated by having a better ground truth segmentation masks and this will increase the accuracy of our DL-based segmentation results.
- We analyzed the performance of GAN models quantitatively and qualitatively across the 7 classes of the I3A dataset. Compared to the CNNs, the performance of GANs for the segmentation task suffers due to the lack of data. In contrast to CNNs which are trained to convergence, GANs are trained until an equilibrium point is reached between the generator and the discriminator. This severely limits the training for GANs for segmentation tasks. We believe these issues can be mitigated by the adding more HEp-2 images to the dataset.
- Domain-Specific Pretraining (DSPT) had a positive impact on all 3 bottom ranking models. In the case of Mid-3 and Top-4 models, DSPT produced mixed results. Specifically in the case of MobileNet-UNet* there was a huge decline in dice score of about 30% compared to the baseline. This suggests that the effectiveness of DSPT depends not only on the dataset characteristics but also on the architectural design of the model.
- Data augmentation strategies exhibited distinct impacts across the model groups. For the Bottom-3 and Mid-3 models, DA-2 consistently delivered better performance across most staining patterns, suggesting that global augmentation strategies with balanced class representation provide more benefit for lower-performing architectures. Conversely, for the Top-4 models, DA-1 emerged as slightly more effective, particularly for minority classes like Golgi and Mitotic Spindle, where the targeted augmentation strategy enhanced representation without overfitting. These findings highlight the nuanced interaction between model architecture and augmentation strategies, underscoring the importance of selecting augmentation schemes tailored to the specific strengths and weaknesses of each model group.
- The performance of HRNet for the minority classes was boosted significantly using DA-1 without loss of performance of other classes. Furthermore, when we visualized the segmentation maps on the test set we found that the augmented model produces well formed cell-like patterns even if the ground truth mask has blob like structures in it. The performance of the pretrained MobileNet-UNet suffered for non-minority classes due to lower number of trainable parameters. Whereas the performance of ResNet50-UNet did not show any noticeable difference.

We note that across the top performing models, namely, MobileNet-UNet, ResNet50-UNet, HRNet and TransUNet* do well on different images within each class. While a model ensemble could be created by just taking the average scores predicted by each model for an image. However, in our experiments we found that different models perform better on different image classes (cell patterns), hence a better ensemble can be obtained by implementing the AdaBoost technique [88]. The first step towards this approach would be to train HRNet model on all images, followed by checking the performance of images where the model performs poorly (some threshold Dice score), and then taking the failed images and training a new model, ResNet50-UNet or MobileNet-UNet, on the subset, and this process is repeated. Finally, each of the three models is used to predict the outcome for each pixel by checking the maximum probability across all models. We have shown that transfer learning can help mitigate some of the poor performance in Golgi and Mitotic Spindle classes. Other recent approaches such as few-shot learning, used traditionally in classification tasks, wherein the drawback of having only a few training samples in particular classes, or diffusion models [27,28] which can generate synthetic images require further consideration.

## 5. Conclusions

Human epithelial type-2 (HEp-2) cell segmentation is a critical task in indirect immunofluorescence (IIF) imaging and plays a key role in diagnosing autoimmune diseases. In this study, we systematically reviewed traditional image processing techniques, machine learning (ML) approaches, and modern deep learning (DL) methodologies for HEp-2 cell segmentation. Our PRISMA-based systematic review revealed that earlier approaches often suffered from lower segmentation accuracy across all cell staining patterns, while state-of-the-art deep convolutional neural network (CNN) and generative adversarial network (GAN) models demonstrated substantial improvements in segmentation outcomes.

We benchmarked 17 different CNN models, including four prominent CNN families—Fully Convolutional Networks (FCN), PSPNet, SegNet, and U-Net—as well as three standalone models—DeepLabV3+, HRNet, and Cellpose. These architectures were combined with three widely used encoders—VGG16, ResNet50, and MobileNet—to evaluate their performance under two training paradigms: no pretraining and ImageNet pretraining in frozen encoder and tunable encoder modes. Our results highlighted that HRNet consistently achieved the highest performance in the no-pretraining group, while MobileNet-UNet emerged as the best-performing model among pretrained architectures, underscoring the importance of efficient model design and transfer learning strategies.

To ensure the statistical validity of our findings, we conducted 5-fold cross-validation and employed the Benjamini–Hochberg (BH) correction for pairwise statistical significance testing. This rigorous analysis identified 81 statistically significant model pairs (*P* < 0.005), providing robust insights into the relative performance differences among the benchmarked architectures. These results emphasize the importance of statistically validated comparisons in segmentation studies.

To further refine model performance, we implemented two data augmentation strategies (DA-1 and DA-2). DA-1, a minority class boosting approach, specifically targeted the underrepresented Mitotic Spindle and Golgi classes and showed better results for Top-4 models, whereas DA-2, a global augmentation strategy, was more effective for the Bottom-3 and Mid-3 models. These findings emphasize the interplay between augmentation strategy and model architecture, highlighting the need for tailored data preprocessing workflows for different model families.

Incorporating Domain-Specific Pretraining (DSPT) using the I3A Task-1 dataset demonstrated varied results. While HRNet and ResNet50-UNet benefited significantly from DSPT, MobileNet-UNet and TransUNet exhibited performance degradation, likely due to overfitting. Interestingly, Bottom-3 models showed consistent improvements with DSPT, while Mid-3 models displayed mixed outcomes. These observations suggest that DSPT can be highly effective but must be carefully adapted to individual model architectures to prevent overfitting.

In addition to CNN experiments, we evaluated the Top-4 performing CNN models within a Pix2pix GAN framework to explore the potential of adversarial training for HEp-2 cell segmentation. Our findings revealed that GAN-based approaches, while promising, suffered from mode collapse and limited generalizability due to data scarcity and training instability. Nevertheless, MobileNet-UNet GAN showed interesting qualitative results, particularly in preserving structural cell patterns in challenging staining classes.

The findings from the patch analysis experiment suggests that methods such as dynamic or content-aware patch sampling strategies could be promising directions for future research. Additionally, ensemble approaches such as AdaBoost offer a promising pathway to leverage the strengths of multiple top-performing models and further enhance segmentation outcomes. Emerging techniques like few-shot learning and diffusion models also present exciting opportunities to overcome data scarcity challenges and improve segmentation accuracy, particularly for underrepresented staining patterns.

In conclusion, this study presents an extensive benchmarking of CNN and GAN models, incorporating a wide range of experimental strategies, including pretraining, augmentation, cross-validation, and statistical significance testing. Our insights not only highlight the current state of HEp-2 cell segmentation but also provide a roadmap for future research directions. We anticipate that these findings will serve as a strong foundation for developing more sophisticated and robust DL models capable of addressing the unique challenges posed by HEp-2 cell segmentation.

## Supplementary Material

1

## Figures and Tables

**Table 1:** HEp-2 segmentation literature for 28 studies. (Abbreviations used for metric evaluation: PVO - Percent Volume Overlap, PVD - Percent Volume Difference, ME - Misclassification Error, RAE - Relative Area Error, MHD - Modified Hausdorff Distance, RDE - Relative Distance Error, SEG - Segmentation accuracy, SE - Sensitivity, JA - Jaccard index. Abbreviations used for Methods: SNFM - Stomped Normal Distribution and Finite Mixture based segmentation, RPrCM - Rough-probabilistic clustering and hidden Markov random field based segmentation method, FCRN-88 - Fully Convolutional Residual Network 88 layers, RouFS - Rough-fuzzy segmentation, cC-GAN - continuous conditional generative adversarial network, CSC - Cell Segmentation and Counting, DSFCN - Deeply Supervised Full Convolutional Network, sRFCM - spatially constrained rough-fuzzy c-means, SBF - Sliding Band Filter.)

Ref.	Method	Results	Dataset
	Image processing		
[42]	Otsu thresholding	NA	In-house: 2573 images 519 diffuse, 482 peripheral, 788 coarse speckled 634 fine speckled, 64 discrete speckled, 86 nucleolar
[43]	Watershed transformation	Avg Sensitivity 94.7%	In-house: 2305 images 456 diffuse, 471 peripheral, 719 coarse speckled 55 fine speckled, 517 discrete speckled, 141 nucleolar
[44]	Adaptive Otsu thresholding	Avg Performance 85.4% (ROIs with 80% + GT overlap)	In-house: 982 images centromere, homogenous, speckled, PmScl, Scl70
[45]	Otsu thresholding	Average correct percentage 89.57%	In-house: 823 images 119 peripheral, 130 centromere, 125 homogenous 113 nucleolar, 119 PmScl, 107 Scl70
[46]	Thresholding	NA	NA
[47]	Auto-learning method	Pixel level Global Avg F-score 59.8%	MIVIA
[48]	Watershed transformation	PVO 89.16%, PVD 21.69%	In-house: 196 images 37 diffused, 29 peripheral, 5 nucleolar 94 coarse speckled, 1 fine speckled, 30 discrete speckled
[49]	Verification based multithresholding	MIVIA: Avg F-score 72.5% ICPR2014: Avg F-score 83.8%	MIVIA and ICPR2014 contest
[50]	Adaptive marker-controlled watershed technique	Avg F-score 71.04%	MIVIA
[51]	Adaptive local thresholding	Accuracy 66.95%	ICPR2014 contest
[52]	Otsu thresholding and Active countor	Avg F-score 84.32	MIVIA: 18/28 images and In-house: 24 images
[53]	Geometric Active Contour	Avg F-score 80.18%	I3A
	Machine learning		
[54]	AIICS system	ME 0.05, RAE 0.11 MHD 89.00, RDE 241.23	NA
[55]	SNFM	Avg F-score 85.93%	MIVIA
[56]	RPrCM	Avg F-score 85.47%	MIVIA
[31]	Random Forest	F-score 84.26%	ICPR2016 contest
[57]	Modified Rough Fuzzy Clustering Otsu thresholding	Avg F-score 86.8%	MIVIA
	Deep learning		
[58]	FCRN-88	Accuracy 89.03%	I3A
[59]	RouFS	Avg F-score 83.42%	MIVIA
[26]	cC-GAN	I3A: Accuracy 86.15% MIVIA: Accuracy 75.27%	I3A and MIVIA
[60]	CSC	CSC-3 model: F-score 85.1% CSC-7 model: F-score 85.2%	I3A
[61]	U-Net (for segmentation)	F-score 96%	I3A
[62]	GAN	F-score 96.40% JA 93.07%	ICPR2016 contest
[32]	DSFCN	SEG 90.10%, SE 89.96% JA 82.68%, Accuracy 96.56%	I3A
[33]	Spatially constrained rough-fuzzy c-means (sRFCM)	Avg F-score 86.1%	MIVIA
[34]	modified SBF	Avg F-score 74.62%	MIVIA
[35]	R-CNN	Accuracy 89%	In-house:4626 images interphase, metaphase, undetermined
[36]	ResNet-34, MobileNetv3 ACM-Net	F-score 97.04%	ICPR2016 contest

**Table 2:** Dice and IoU scores of UNet models trained with varying patch counts per image, evaluated on the I3A test set.

Patches	Dice	IOU
4	0.742	0.590
7	0.773	0.630
12	0.781	0.641
20	0.769	0.625
30	0.779	0.638

**Table 3:** Performance of deep learning models on the I3A test set. All other cases represent random weight initialization.

Model	Encoder	AU-PR	AU-ROC	Acc.	Dice	IOU	Prec.	Sens.	Spec.
MobileNet-SegNet	MobileNet	0.579	0.833	0.842	0.495	0.329	0.575	0.435	0.930
ResNet50-Unet^b^	ResNet50	0.721	0.911	0.861	0.561	0.390	0.740	0.491	0.962
VGG-PSPNet^a^	VGG16	0.734	0.932	0.879	0.564	0.393	0.786	0.440	0.974
VGG-PSPNet^b^	VGG16	0.746	0.942	0.880	0.578	0.406	0.801	0.452	0.953
ResNet50-Unet^a^	ResNet50	0.718	0.901	0.878	0.600	0.428	0.723	0.512	0.957
VGG-SegNet^b^	VGG16	0.721	0.898	0.876	0.611	0.440	0.735	0.541	0.943
SegNet	vanilla	0.839	0.943	0.900	0.630	0.460	0.923	0.478	0.991
VGG-SegNet	VGG16	0.762	0.920	0.900	0.681	0.516	0.784	0.602	0.964
MobileNet-SegNet^b^	MobileNet	0.828	0.932	0.899	0.688	0.524	0.779	0.678	0.959
SegFormer	custom	0.825	0.946	0.913	0.690	0.528	0.781	0.683	0.967
Cellpose	custom	0.886	0.968	0.850	0.693	0.531	0.545	0.953	0.827
VGG-UNet^a^	VGG16	0.828	0.921	0.916	0.710	0.550	0.920	0.578	0.989
FCN8-VGG^b^	VGG16	0.858	0.931	0.912	0.741	0.589	0.861	0.788	0.969
VGG-UNet^b^	VGG16	0.828	0.921	0.916	0.753	0.604	0.877	0.621	0.972
FCN8-VGG^a^	VGG16	0.882	0.970	0.932	0.797	0.663	0.856	0.746	0.973
TransUnet	ResNet50	0.872	0.967	0.933	0.799	0.666	0.819	0.821	0.961
FCN32	vanilla	0.921	0.980	0.937	0.803	0.671	0.909	0.720	0.984
PSPNet	vanilla	0.915	0.979	0.939	0.816	0.690	0.882	0.760	0.978
VGG-PSPNet	VGG16	0.899	0.976	0.936	0.816	0.690	0.838	0.795	0.967
FCN8-MobileNet^a^	MobileNet	0.905	0.974	0.939	0.816	0.690	0.875	0.764	0.976
VGG-SegNet^a^	VGG16	0.906	0.974	0.940	0.826	0.703	0.852	0.801	0.970
DeepLabV3+	ResNet50	0.907	0.980	0.943	0.843	0.734	0.831	0.880	0.959
ResNet50-PSPNet	ResNet50	0.926	0.982	0.947	0.844	0.731	0.881	0.811	0.976
FCN8-MobileNet^b^	MobileNet	0.933	0.989	0.954	0.846	0.733	0.857	0.904	0.968
FCN8-MobileNet	MobileNet	0.916	0.977	0.943	0.848	0.737	0.812	0.888	0.955
ResNet50-SegNet	ResNet50	0.960	0.989	0.955	0.862	0.758	0.959	0.783	0.993
MobileNet-SegNet^a^	MobileNet	0.948	0.985	0.956	0.872	0.772	0.907	0.839	0.981
FCN8	vanilla	0.948	0.987	0.957	0.877	0.780	0.887	0.867	0.976
VGG-UNet	VGG16	0.963	0.990	0.958	0.889	0.800	0.840	0.944	0.961
FCN8-ResNet50	ResNet50	0.961	0.989	0.962	0.892	0.805	0.916	0.869	0.983
MobileNet-UNet	MobileNet	0.964	0.991	0.960	0.892	0.805	0.851	0.937	0.965
FCN8-VGG	VGG16	0.960	0.986	0.963	0.894	0.809	0.922	0.868	0.984
TransUnet^b^	ResNet50	0.965	0.993	0.964	0.894	0.808	0.862	0.931	0.977
MobileNet-UNet^b^	MobileNet	0.912	0.983	0.951	0.895	0.810	0.841	0.899	0.979
ResNet50-UNet	ResNet50	0.963	0.990	0.963	0.898	0.814	0.890	0.905	0.976
MobileNet-UNet^a^	MobileNet	0.968	0.992	0.966	0.901	0.820	0.937	0.867	0.987
HRNet	custom	0.967	0.989	0.966	0.905	0.826	0.894	0.916	0.976
TransUnet^a^	ResNet50	0.978	0.995	0.972	0.921	0.857	0.930	0.916	0.985

a Model indicate initialization with ImageNet weights and frozen encoders.

b Model indicate initialization with ImageNet weights and tunable encoders.

**Table 4:** Dice scores from 5-fold cross-validation for pretrained models on the I3A test set. Mean and standard deviation represent the average model performance across the five folds.

Model	Encoder	Fold-1	Fold-2	Fold-3	Fold-4	Fold-5	Mean	Std. Dev.
MobileNet-SegNet	MobileNet	0.462	0.484	0.440	0.534	0.489	0.482	0.035
ResNet50-Unet**	ResNet50	0.534	0.515	0.504	0.567	0.538	0.532	0.024
VGG-PSPNet*	VGG16	0.543	0.566	0.555	0.567	0.547	0.556	0.011
VGG-PSPNet**	VGG16	0.585	0.595	0.604	0.585	0.586	0.591	0.008
SegNet	vanilla	0.638	0.678	0.612	0.585	0.539	0.610	0.053
ResNet50-Unet*	ResNet50	0.629	0.614	0.590	0.630	0.615	0.616	0.016
VGG-SegNet**	VGG16	0.643	0.607	0.607	0.674	0.660	0.638	0.030
MobileNet-SegNet**	MobileNet	0.671	0.677	0.652	0.670	0.721	0.678	0.026
VGG-SegNet	VGG16	0.705	0.692	0.696	0.687	0.705	0.697	0.008
VGG-UNet*	VGG16	0.696	0.699	0.691	0.711	0.706	0.701	0.008
SegFormer	custom	0.702	0.756	0.707	0.692	0.722	0.716	0.025
Cellpose	custom	0.732	0.735	0.697	0.683	0.743	0.718	0.026
FCN8-VGG**	VGG16	0.754	0.754	0.709	0.745	0.642	0.721	0.048
VGG-UNet**	VGG16	0.769	0.783	0.744	0.761	0.775	0.766	0.015
FCN8-VGG*	VGG16	0.785	0.814	0.777	0.768	0.772	0.783	0.018
TransUnet	ResNet50	0.788	0.793	0.801	0.818	0.745	0.789	0.027
VGG-PSPNet	VGG16	0.799	0.784	0.801	0.785	0.800	0.794	0.008
FCN32	vanilla	0.796	0.798	0.810	0.785	0.793	0.796	0.009
PSPNet	vanilla	0.792	0.830	0.835	0.824	0.803	0.817	0.018
FCN8-MobileNet*	MobileNet	0.827	0.826	0.817	0.817	0.820	0.821	0.005
VGG-SegNet*	VGG16	0.835	0.828	0.819	0.823	0.848	0.830	0.012
TransUnet**	ResNet50	0.849	0.838	0.842	0.831	0.846	0.841	0.007
DeepLabV3+	ResNet50	0.860	0.842	0.825	0.828	0.853	0.842	0.015
FCN8-MobileNet	MobileNet	0.856	0.842	0.831	0.829	0.853	0.842	0.012
FCN8-MobileNet**	MobileNet	0.835	0.832	0.848	0.853	0.863	0.846	0.013
ResNet50-PSPNet	ResNet50	0.852	0.845	0.853	0.855	0.848	0.851	0.004
MobileNet-SegNet*	MobileNet	0.861	0.870	0.883	0.877	0.866	0.871	0.009
ResNet50-SegNet	ResNet50	0.885	0.879	0.861	0.873	0.873	0.874	0.009
FCN8	vanilla	0.868	0.884	0.903	0.867	0.885	0.882	0.015
VGG-UNet	VGG16	0.880	0.899	0.882	0.865	0.901	0.885	0.015
MobileNet-UNet **	MobileNet	0.904	0.889	0.889	0.882	0.885	0.890	0.009
FCN8-ResNet50	ResNet50	0.904	0.896	0.890	0.873	0.887	0.890	0.011
MobileNet-UNet	MobileNet	0.893	0.894	0.892	0.890	0.895	0.893	0.002
FCN8-VGG	VGG16	0.892	0.896	0.888	0.890	0.898	0.893	0.004
ResNet50-UNet	ResNet50	0.897	0.900	0.894	0.889	0.901	0.896	0.005
MobileNet-UNet *	MobileNet	0.898	0.893	0.911	0.891	0.897	0.898	0.008
HRNet	custom	0.911	0.909	0.901	0.894	0.930	0.909	0.013
TransUnet*	ResNet50	0.948	0.902	0.916	0.895	0.899	0.912	0.022

**Table 5:** BH-corrected p-values for pairwise statistical comparisons between pretrained models on the I3A test set. Comparisons with p-values below 0.005 are considered statistically significant.

	ResNet50-Unet**	VGG-PSPNet*	VGG-PSPNet**	ResNet50-Unet*	MobileNet-SegNet**	VGG-SegNet	VGG-UNet*	VGG-PSPNet	FCN32	FCN8-MobileNet*	VGG-SegNet*
FCN8-MobileNet**	4.27e–03	2.37e–03	3.03e–03	–	–	–	–	–	–	–	–
ResNet50-PSPNet	4.34e–03	3.89e–04	3.27e–04	4.10e–03	–	2.94e–03	1.87e–03	–	–	–	–
ResNet50-SegNet	3.56e–03	8.67e–04	1.70e–03	5.02e–04	–	1.82e–03	2.42e–03	–	–	–	–
MobileNet-UNet	3.87e–03	2.40e–04	1.18e–04	2.18e–03	–	2.54e–04	5.88e–04	–	–	2.22e–03	–
FCN8-VGG	3.62e–03	2.96e–04	2.81e–04	1.81e–03	–	3.29e–04	4.21e–04	–	–	3.74e–03	–
FCN32	–	2.89e–03	2.31e–05	–	–	–	–	–	–	–	–
PSPNet	–	7.70e–04	2.09e–03	–	–	–	–	–	–	–	–
FCN8-MobileNet*	–	9.69e–04	8.63e–04	4.70e–03	–	2.10e–03	–	–	–	–	–
VGG-SegNet*	–	4.70e–03	–	–	–	2.05e–03	–	–	–	–	–
TransUnet**	–	2.56e–03	7.78e–04	–	–	1.61e–06	–	–	–	–	–
MobileNet-SegNet*	–	1.22e–04	6.46e–05	–	–	–	4.75e–03	–	–	–	–
FCN8	–	1.65e–03	9.38e–05	–	–	–	–	–	–	–	–
VGG-UNet	–	2.47e–03	1.39e–03	–	–	4.03e–03	–	–	–	–	–
MobileNet-UNet **	–	1.27e–03	5.71e–04	2.42e–03	–	3.30e–04	–	–	–	–	–
FCN8-ResNet50	–	1.89e–03	6.11e–04	–	–	6.01e–04	–	–	–	–	–
ResNet50-UNet	–	4.00e–04	1.58e–04	2.74e–03	–	1.54e–04	1.19e–03	–	–	2.57e–03	–
MobileNet-UNet *	–	7.04e–04	2.17e–05	–	–	7.74e–04	–	1.51e–03	4.96e–04	–	–
HRNet	–	2.34e–03	1.56e–03	4.45e–03	3.37e–03	4.85e–04	4.46e–03	–	–	–	2.13e–03
VGG-PSPNet	–	–	1.29e–03	–	–	1.27e–03	–	–	–	–	–
FCN8-MobileNet	–	–	4.33e–03	–	–	6.76e–04	–	–	–	–	–
DeepLabV3+	–	–	–	4.21e–03	–	3.89e–03	–	–	–	–	–

**Table 6:** Classwise Dice scores of all the benchmarked DL models on the test set I3A dataset.

Model	Homogeneous	Centromere	Speckled	Nuclear Membrane	Nucleolar	Mitotic Spindle	Golgi
MobileNet-SegNet	0.370	0.637	0.350	0.160	0.324	0.207	0.193
ResNet50-Unet^b^	0.548	0.616	0.419	0.379	0.355	0.197	0.211
VGG-PSPNet^a^	0.626	0.627	0.613	0.469	0.421	0.383	0.247
VGG-PSPNet^b^	0.724	0.646	0.655	0.488	0.399	0.288	0.183
SegNet	0.772	0.778	0.685	0.577	0.452	0.121	0.100
ResNet50-UNet^a^	0.708	0.664	0.609	0.358	0.389	0.349	0.178
VGG-SegNet^b^	0.782	0.723	0.669	0.499	0.448	0.238	0.147
MobileNet-SegNet^b^	0.801	0.787	0.674	0.544	0.489	0.272	0.172
VGG-SegNet	0.796	0.848	0.799	0.528	0.616	0.246	0.139
VGG-UNet^a^	0.869	0.858	0.845	0.585	0.588	0.194	0.183
SegFormer	0.810	0.724	0.745	0.574	0.593	0.435	0.298
Cellpose	0.789	0.855	0.778	0.616	0.683	0.545	0.513
FCN8-VGG^b^	0.841	0.824	0.790	0.681	0.677	0.517	0.489
VGG-UNet^b^	0.889	0.860	0.812	0.779	0.693	0.583	0.296
FCN8-VGG^a^	0.923	0.888	0.907	0.721	0.739	0.324	0.354
TransUnet	0.900	0.834	0.881	0.704	0.756	0.532	0.319
VGG-PSPNet	0.860	0.851	0.848	0.819	0.830	0.683	0.544
FCN32	0.833	0.832	0.822	0.815	0.810	0.757	0.677
PSPNet	0.859	0.841	0.840	0.828	0.822	0.749	0.704
FCN8-MobileNet^a^	0.918	0.893	0.891	0.789	0.772	0.497	0.336
VGG-SegNet^a^	0.905	0.891	0.895	0.820	0.825	0.540	0.494
TransUnet^b^	0.897	0.881	0.884	0.846	0.837	0.668	0.512
DeepLabV3+	0.902	0.882	0.868	0.813	0.826	0.674	0.564
FCN8-MobileNet	0.898	0.883	0.889	0.810	0.840	0.726	0.507
FCN8-MobileNet^b^	0.919	0.903	0.877	0.892	0.853	0.719	0.642
ResNet50-PSPNet	0.873	0.858	0.857	0.857	0.835	0.820	0.771
MobileNet-SegNet^a^	0.923	0.906	0.910	0.893	0.854	0.786	0.581
ResNet50-SegNet	0.908	0.869	0.890	0.898	0.835	0.816	0.784
FCN8	0.938	0.915	0.918	0.884	0.881	0.739	0.597
VGG-UNet	0.928	0.900	0.916	0.879	0.885	0.833	0.788
MobileNet-UNet^b^	0.940	0.909	0.889	0.900	0.886	0.838	0.778
FCN8-ResNet50	0.932	0.903	0.919	0.908	0.881	0.842	0.786
MobileNet-UNet	0.944	0.901	0.928	0.893	0.881	0.841	0.746
FCN8-VGG	0.941	0.915	0.924	0.913	0.888	0.813	0.761
ResNet50-UNet	0.940	0.916	0.916	0.905	0.893	0.838	0.791
MobileNet-UNet^a^	0.950	0.922	0.928	0.921	0.888	0.817	0.686
HRNet	0.945	0.921	0.930	0.928	0.898	0.871	0.830
TransUnet^a^	0.951	0.930	0.935	0.923	0.901	0.860	0.794
Mean ± Std. Dev.	0.849 ± 0.122	0.837 ± 0.093	0.813 ± 0.140	0.731 ± 0.194	0.722 ± 0.185	0.583 ± 0.242	0.492 ± 0.243

a Pretrained with frozen encoder.

b Pretrained with tunable encoder and rest are without pretraining.

**Table 7:** Dice and IoU scores of Top-4, Mid-3, and Bottom-3 models on the domain-specific pretraining task using the I3A Task-1 dataset.

Model	Encoder	Dice	IOU
MobileNet-SegNet	MobileNet	0.944	0.894
ResNet50-Unet**	ResNet50	0.925	0.860
VGG-PSPNet*	VGG16	0.909	0.833
VGG-UNet*	VGG16	0.942	0.890
FCN8-MobileNet*	MobileNet	0.928	0.866
FCN8	vanilla	0.953	0.910
ResNet50-UNet	ResNet50	0.962	0.927
MobileNet-UNet*	MobileNet	0.935	0.878
HRNet	custom	0.961	0.925
TransUNet*	ResNet50	0.954	0.913

**Table 8:** Augmentation techniques used in RandAugment.

Augmentation	Min.	Max.
Translation along X-axis	−100	+100
Translation along Y-axis	−100	+100
Rotation	−30	+30
Histogram Equalization	–	–
Identity	–	–
Brightness	0.3	2.5
Sharpness	0.3	2.5
Contrast	0.3	2.5

**Table 9:** Dice score comparison of the Bottom-3, Mid-3 and Top-4 models under different conditions on the Test Set. DSPT: Domain Specific Pretraining. DA-1: Augmentation Strategy - 1, DA-2 Augmentation Strategy-2, GAN: model deployed as Generator in a GAN segmentation framework.

Model	Baseline		DSPT		DA-1		DA-2		GAN	
	Dice	IOU	Dice	IOU	Dice	IOU	Dice	IOU	Dice	IOU
MobileNet-SegNet	0.495	0.329	0.588	0.416	0.542	0.372	0.571	0.400	–	–
ResNet50-Unet**	0.561	0.390	0.636	0.466	0.572	0.401	0.624	0.453	–	–
VGG-PSPNet*	0.564	0.393	0.583	0.411	0.524	0.355	0.615	0.444	–	–
VGG-UNet*	0.710	0.550	0.633	0.463	0.724	0.567	0.776	0.634	–	–
FCN8-MobileNet*	0.816	0.690	0.840	0.724	0.714	0.555	0.877	0.781	–	–
FCN8	0.877	0.780	0.869	0.768	0.858	0.751	0.825	0.702	–	–
ResNet50-UNet	0.898	0.814	0.916	0.845	0.888	0.798	0.862	0.766	0.836	0.718
MobileNet-UNet*	0.901	0.820	0.603	0.432	0.735	0.581	0.885	0.800	0.867	0.766
HRNet	0.905	0.826	0.922	0.855	0.909	0.834	0.902	0.823	0.843	0.729
TransUNet*	0.921	0.857	0.810	0.710	0.881	0.791	0.825	0.717	0.879	0.783

**Table 10:** Classwise Dice scores of the Bottom-3 models under the DSPT, DA-1 and DA-2 paradigms.

Model	Homogeneous	Centromere	Speckled	Nuclear Membrane	Nucleolar	Mitotic Spindle	Golgi
MobileNet-SegNet	0.370	0.637	0.350	0.160	0.324	0.207	0.193
MobileNet-SegNet DSPT	0.568	0.664	0.339	0.428	0.444	0.347	0.245
MobileNet-SegNet DA-1	0.410	0.656	0.377	0.193	0.279	0.278	0.298
MobileNet-SegNet DA-2	0.683	0.641	0.665	0.389	0.471	0.345	0.215
ResNet50-Unet**	0.548	0.616	0.419	0.379	0.355	0.197	0.211
ResNet50-Unet** DSPT	0.798	0.775	0.685	0.426	0.379	0.233	0.198
ResNet50-Unet** DA-1	0.562	0.598	0.447	0.375	0.414	0.354	0.298
ResNet50-Unet** DA-2	0.711	0.688	0.494	0.415	0.472	0.262	0.185
VGG-PSPNet*	0.626	0.627	0.613	0.469	0.421	0.383	0.247
VGG-PSPNet* DSPT	0.687	0.619	0.642	0.495	0.398	0.354	0.299
VGG-PSPNet* DA-1	0.646	0.363	0.542	0.473	0.463	0.431	0.302
VGG-PSPNet* DA-2	0.820	0.733	0.685	0.515	0.498	0.212	0.139

**Table 11:** Classwise Dice scores of the Mid-3 models under the DSPT, DA-1 and DA-2 paradigms.

Model	Homogeneous	Centromere	Speckled	Nuclear Membrane	Nucleolar	Mitotic Spindle	Golgi
VGG-UNet*	0.869	0.858	0.845	0.585	0.588	0.194	0.183
VGG-UNet* DSPT	0.767	0.766	0.750	0.593	0.497	0.144	0.233
VGG-UNet* DA-1	0.848	0.862	0.741	0.593	0.585	0.267	0.256
VGG-UNet* DA-2	0.892	0.877	0.851	0.626	0.601	0.285	0.303
FCN8-MobileNet*	0.918	0.893	0.891	0.789	0.772	0.497	0.336
FCN8-MobileNet* DSPT	0.887	0.868	0.852	0.857	0.760	0.576	0.633
FCN8-MobileNet* DA-1	0.848	0.622	0.887	0.543	0.741	0.568	0.429
FCN8-MobileNet* DA-2	0.934	0.901	0.886	0.892	0.767	0.541	0.543
FCN8	0.938	0.915	0.918	0.884	0.881	0.739	0.597
FCN8 DSPT	0.924	0.889	0.919	0.905	0.837	0.755	0.621
FCN8 DA-1	0.841	0.872	0.906	0.863	0.848	0.757	0.641
FCN8 DA-2	0.910	0.842	0.849	0.795	0.834	0.677	0.566

**Table 12:** Classwise Dice scores of the Top-4 models under the DSPT, DA-1, DA-2 and GAN paradigms.

Model	Homogeneous	Centromere	Speckled	Nuclear Membrane	Nucleolar	Mitotic Spindle	Golgi
MobileNet-UNet*	0.950	0.922	0.928	0.921	0.888	0.817	0.686
MobileNet-UNet* DSPT	0.722	0.671	0.616	0.351	0.374	0.331	0.173
MobileNet-UNet* DA-1	0.892	0.564	0.818	0.910	0.568	0.729	0.667
MobileNet-UNet* DA-2	0.925	0.898	0.910	0.891	0.855	0.829	0.714
MobileNet-UNet* GAN	0.939	0.908	0.914	0.857	0.871	0.654	0.558
ResNet50-UNet	0.940	0.916	0.916	0.905	0.893	0.838	0.791
ResNet50-UNet DSPT	0.949	0.923	0.934	0.927	0.899	0.872	0.768
ResNet50-UNet DA-1	0.927	0.886	0.916	0.900	0.880	0.856	0.807
ResNet50-UNet DA-2	0.911	0.887	0.895	0.845	0.832	0.764	0.623
ResNet50-UNet GAN	0.933	0.900	0.906	0.808	0.843	0.607	0.465
HRNet	0.945	0.921	0.930	0.928	0.898	0.871	0.830
HRNet DSPT	0.951	0.926	0.939	0.938	0.907	0.889	0.793
HRNet DA-1	0.946	0.911	0.935	0.928	0.897	0.889	0.866
HRNet DA-2	0.933	0.905	0.919	0.909	0.883	0.870	0.772
HRNet GAN	0.912	0.892	0.883	0.806	0.857	0.771	0.639
TransUNet*	0.954	0.931	0.935	0.934	0.900	0.879	0.757
TransUNet* DSPT	0.900	0.859	0.887	0.734	0.770	0.619	0.340
TransUNet* DA-1	0.923	0.890	0.894	0.897	0.846	0.849	0.716
TransUNet* DA-2	0.882	0.878	0.870	0.787	0.783	0.674	0.510
TransUNet* GAN	0.935	0.905	0.912	0.887	0.847	0.794	0.539

**Table 13:** Comparison of model advantages and disadvantages.

Model/Method	Advantages	Disadvantages
Domain-Specific Pretraining (DSPT)	Improved segmentation performance on Bottom-3 models.	Benefits are inconsistent across Mid-3 and Top-4 model architectures.
Augmentation Strategy - 1 (DA-1)	Focused improvement on minority classes (Mitotic Spindle, Golgi).	Limited benefits for non-minority classes; risk of overfitting.
Augmentation Strategy - 2 (DA-2)	Balanced augmentation across all classes; better generalization. Benefits lower baseline models	Inconsistent benefits on Top-4 models; .
GAN Framework (Pix2Pix)	Potential to refine segmentation boundaries in adversarial setups. Enables models to learn true shape and texture of cells	Lower performance compared to CNN models; instability during training.
HRNet	Consistent high performance across most classes; strong generalization. Low sensitivity to augmentation and pretraining strategies	Susceptible to memorization for underrepresented classes.
MobileNet-UNet	Lightweight architecture; performs well on pretrained setups.	Struggles with highly complex staining patterns in minority classes.
ResNet50-UNet	Balanced performance; robust to architectural changes.	Moderate adaptability in low-data regimes.
TransUNet	Leverages transformers for global context understanding.	High computational requirements; sensitive to augmentation strategies.

## Footnotes

2 https://hep2.unisa.it.

3 https://github.com/prasathlab/Hep2-Segmentation.

4 https://www.prasathlab.com/research/microscopy/hep-2segzoo.

5 https://mivia.unisa.it/datasets/biomedical-image-datasets/hep2-image-dataset.

## Appendix A. Supplementary data

Supplementary material related to this article can be found online at https://doi.org/10.1016/j.compbiomed.2025.110150.
