# Supplementary material for: Benchmarking HEp-2 cell segmentation methods in indirect immunofluorescence images - standard models to deep learning
Source: Comput Biol Med. Author manuscript; Available in PMC 2026 Jun 14. (PMC13264518; doi:10.1016/j.compbiomed.2025.110150)
Supplement: 1 [file NIHMS2174808-supplement-1.pdf]

Supplementary

Balaji S. Iyer[1,4], Smruti Deoghare[1,3], Krish Ranjan[‡,5], Bruce J. Aronow[1,2,3], V. B. Surya Prasath[1,2,3,4,*]

I. Additional Literature Review

Swaroopa et al. [1] proposed a novel segmentation method for HEp-2 cell images using a Threshold-Based Fusion Technique. Their approach combines multiple thresholding techniques, Gaussian filtering, and fuzzy c-means (FCM) clustering to achieve enhanced segmentation performance. Initially, raw HEp-2 cell images are preprocessed using a Gaussian filter to remove noise, followed by adaptive thresholding for region-specific segmentation. The segmented images are then refined using binary smoothing and adaptive histogram equalization to improve local contrast and edge definition. The segmentation output from adaptive thresholding and FCM clustering is fused to create the final segmentation masks, enhancing both accuracy and robustness. Their experiments were performed on the SNPHep-2 dataset, and results were evaluated using Accuracy, Sensitivity, Dice Coefficient, and Jaccard Coefficient. The proposed method achieved superior performance compared to standard techniques such as global thresholding, Otsu's thresholding, and multilevel thresholding, with an Accuracy of 97.66%, Sensitivity of 95.68%, Dice Coefficient of 94.44%, and Jaccard Coefficient of 89.47%. The authors highlighted that the fusion of threshold-based techniques with FCM clustering resulted in segmentation masks that preserved essential biological features, offering significant improvements over traditional methods.

Dulaimi et al. [2] proposed a method for classifying HEp-2 staining pattern images using an adapted Multilayer Perceptron (MLP) neural network. Their approach focuses on addressing key challenges such as intra-class variation, overfitting, and large-scale data volume. The method incorporates two hidden layers in the MLP: the first hidden layer computes Higher-Order Spectra (HOS) features, including mean, scale, skewness, and kurtosis, while the second hidden layer employs a Softmax activation function to classify images into six staining pattern classes (Homogeneous, Speckled, Nucleolar, Centromere, Nuclear Membrane, and Golgi).

The model was evaluated using datasets from ICPR2014 and ICPR2016 competitions and achieved a classification accuracy of 87.5% without data augmentation and 90.3% with data augmentation. Compared to baseline and previously published methods, their adapted MLP model demonstrated improved performance in terms of mean classification accuracy (MCA). This study highlights the importance of leveraging statistical features from cell shape information to improve HEp-2 cell classification performance.

Anaam et al. [3] proposed a deep active learning (DAL) framework for automatic mitotic cell detection in HEp-2 specimen images. Their approach bypasses the need for manual segmentation by directly detecting mitotic cells using entire specimen images. The framework incorporates a DAL strategy to iteratively refine annotations for bounding boxes around mitotic cells, reducing the dependency on extensive manual labeling. Pre-processing with Contrast-Limited Adaptive Histogram Equalization (CLAHE) was employed to enhance image quality. Two state-of-the-art object detection models, YOLO and Faster R-CNN, were benchmarked on the I3A Task-2 dataset using a 5-fold cross-validation strategy. The YOLO detector achieved superior performance with an average recall of 90.01%, precision of 88.30%, and mean Average Precision (mAP) of 81.53%, outperforming Faster R-CNN, which achieved 86.98% recall, 85.28% precision, and 78.50% mAP. Their results demonstrate the effectiveness of DAL in improving annotation accuracy and suggest YOLO as a preferable model for mitotic cell detection in real-time clinical applications.

II. Visual Analysis of Segmentation Results

A visual analysis of the input images, ground truth and the predicted masks of different models is particularly helpful for semantic segmentation tasks. A visual exploration highlights the generalizability of deep learning models and sheds light on the challenges in the data. Through our experiments we found that HRNet not only achieved the best overall Dice score but also outperformed all other models across all cell patterns. In order to perform a systematic analysis, we chose HRNet as our reference model. From the held out test set we selected the best and worst performing images for HRNet for each of the 7 staining patterns to obtain 14 images. We then contrast the performance of our top three models i.e HRNet, pretrained MobileNet-UNet and ResNet50-UNet on these 14 images. The top 3 rows of Figure 1 visualize the input image, the contrast enhanced (using CLAHE) version of the image and the associated ground truth mask for the best performing images for each class. The lack of contrast in the images is immediately apparent. For example, even in the Homogeneous class which has better contrast compared to other images, the texture of the cell and the surrounding structure is imperceptible in the raw image but is discernible in the contrast enhanced image. The bottom three rows in the figure show the predicted mask of HRNet, pretrained MobileNet-UNet and ResNet50-UNet respectively. Visually we notice that all three models have high concurrence with the ground truth mask. Table I presents a quantitative assessment of model performance for these images.

The top 3 rows of Figure 2 shows the input grayscale image, the contrast enhanced (using CLAHE) version of the image and the assosciated ground truth mask for the worst performing images for each class. The problem of poor

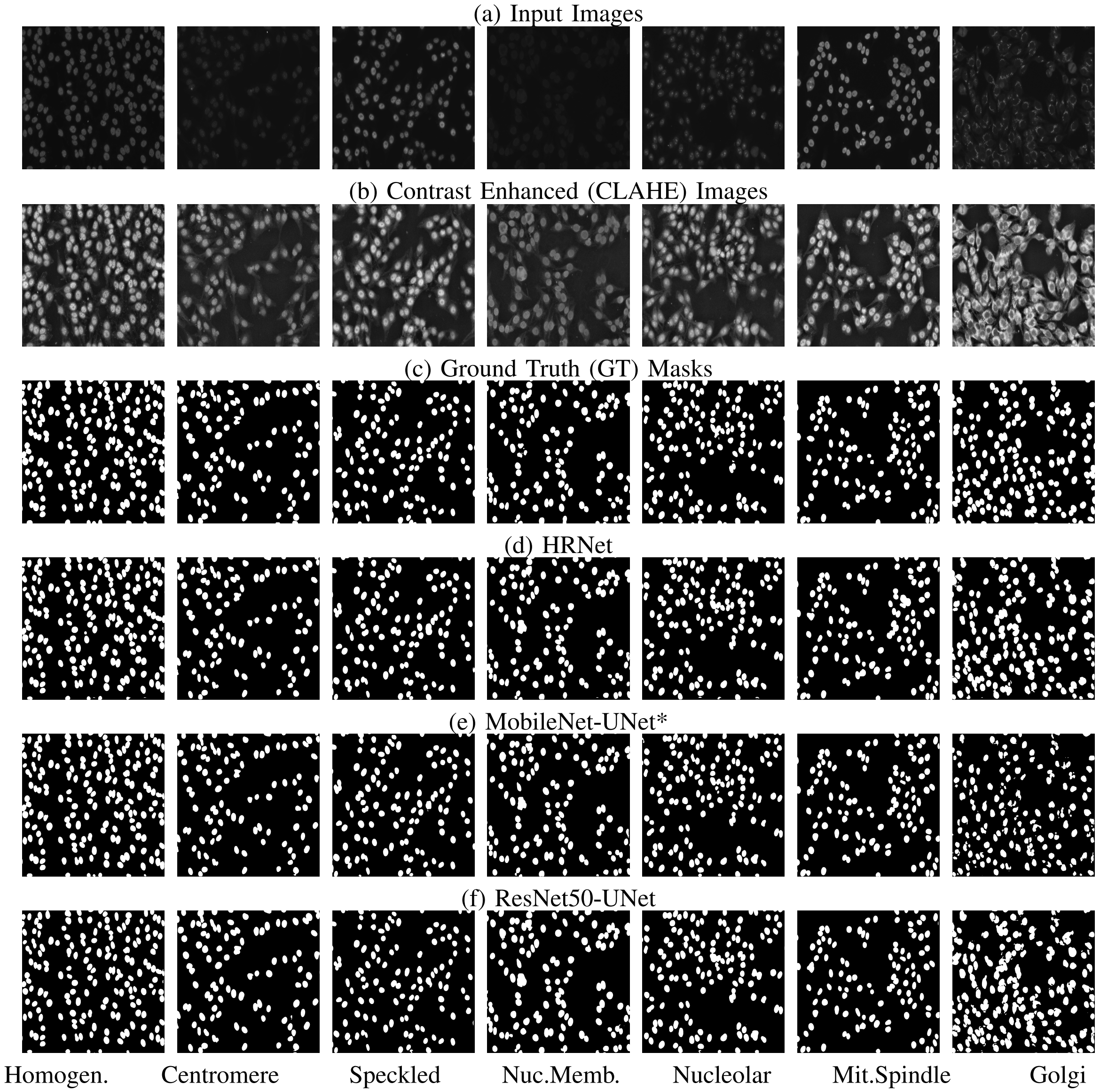

Fig. 1. Classwise segmentation results on selected good GT HEp-2 images. (a) Input grayscale, (b) contrast enhanced (CLAHE), and corresponding (c) ground truth (GT) masks. Segmentation results of (d) HRNet, (e) MobileNet-UNet*, and (f) ResNet50-UNet models. (*Pretrained).

TABLE I
CLASSWISE PERFORMANCE OF THE SELECTED TOP THREE CNN MODELS ON IMAGES PRESENTED IN FIGURE 1. (*PRETRAINED MODEL)

Model	Homogeneous	Centromere	Speckled	Nuclear Membrane	Nucleolar	Mitotic Spindle	Golgi
HRNet	0.971	0.954	0.965	0.951	0.952	0.963	0.880
MobileNet-UNet*	0.970	0.940	0.962	0.948	0.946	0.966	0.792
ResNet50-UNet	0.952	0.954	0.932	0.932	0.945	0.958	0.830

contrast is more acute for these images. For example for the nucleolar and golgi class even contrast enhanced images are visually unsatisfying. A closer scrutiny of the images and masks (top 3 rows) reveals not just poor contrast in the images but also the unfaithful nature of the ground truth masks. For the images in the I3A dataset the ground truth was obtained from the DAPI channel rather than manual annotation. This means that the ground truth mask are silver-standard rather than gold standard. During training the models are therefore exposed to cases with correct ground truth (Figure 1) and incorrect (or partially correct) ground truth. Model evaluation is particularly confounded by the presence of noise in labels due to the fact that performance metrics in such a case can be misleading. Table I shows the performance of the models for these images. For the homogeneous case, MobileNet-UNet Pre. and ResNet50-UNet achieved very high Dice scores while HRNet detects blob like structures indicative of false positive predictions. For the centromere class the higher Dice score of HRNet and ResNet50-UNet is driven primarily by the poor ground truth and although MobileNet-UNet has the lowest Dice score it is visually far more appealing. For the speckled, numem and nucleolar images the ground truth is reliable and

quantitatively and visually MobileNet-UNet Pre. outperforms the other two models. For the mitotic spindle and golgi class, the ground truth is rather poor and a vision test is more definitive than a quantitative assessment. In both classes we can visually observe the better generalizability of MobileNet-UNet Pre. compared to other models.

We can evaluate the effect of fine tuning the model with limited image augmentation on these images by visualizing the predicted masks before and after augmentation. The bottom two rows of Figure 3 shows the performance of HRNet before and after augmentation on the good images. Similarly, we can visualize for the bad images the predicted mask of HRNet before and after augmentation Figure 4. Comparing the two figures it is clear that for the good images the performance of HRNet remains more or less the same but for the bad images HRNet predictions have improved significantly. This is particularly evident in the Mitotic Spindle, Nucleolar and Centromere classes for which the pre-augmentation network predicted blob like structures, whereas the post-augmentation network predicts less blob-like structures and more cell structures.

As noted earlier, the performance of pretrained MobileNet-UNet saw a drastic drop due to the lack of trainable parameters. Nevertheless, we visualize the good and bad images for this network as well. In the good case we do not observe any discernible difference between the pre- and post-augmentation predictions. However, in the bad case we perceive a pronounced difference for the Centromere, Nucleolar and Golgi classes. For these classes, the number of undetected cells increased noticeably and a number of cells were only partially segmented out.

Figure 7 and 8 show the good and bad image cases for ReseNet50-UNet respectively. Despite the noisy ground truth labels, the introduction of augmentation acts as a regularizer and prevents the model from overfitting and guides the model towards discovering more regular cell structures rather than blob-like structures.

In Figure 9 we show bad images cases visualizing the segmentation masks of HRNet, MobileNet-UNet, and ResNet50-UNet with augmentation strategy - 1. As can be seen, the augmented models obtain better segmentation results by capturing cell-specific masks instead of the blobs which are wrongly labelled in the GT masks. The blue colored regions are present in the GT masks and our augmented models avoid these spurious artifacts. In a similar spirit we can visually assess the efficacy of GANs in producing segmentation maps of images. Figure 10 and Figure 11 show the visualizations for the good and bad image cases respectively. For the good case, GAN models preformed well for all classes except the Golgi class. But in the bad case model, performance is unsatisfactory, especially for Mitotic Spindle and Golgi patterns.

References

[1] H. Swaroopa, B. N. Jagadale, O. A. M. Farhan, V. H. Alnaggar, and T. Abhisheka, "Human epithelial cell image analysis and segmentation using threshold based fusion technique," *Biomedical & Pharmacology Journal*, vol. 17, no. 1, pp. 443–452, 2024.

[2] K. Al-Dulaimi, J. Banks, A. Al-Sabaawi, K. Nguyen, V. Chandran, and I. Tomeo-Reyes, "Classification of hep-2 staining pattern images using adapted multilayer perceptron neural network-based intra-class variation of cell shape," *Sensors*, vol. 23, no. 4, p. 2195, 2023.

[3] A. Anaam, M. A. Al-Antari, J. Hussain, N. Abdel Samee, M. Alabdulhafith, and A. Gofuku, "Deep active learning for automatic mitotic cell detection on hep-2 specimen medical images," *Diagnostics*, vol. 13, no. 8, p. 1416, 2023.

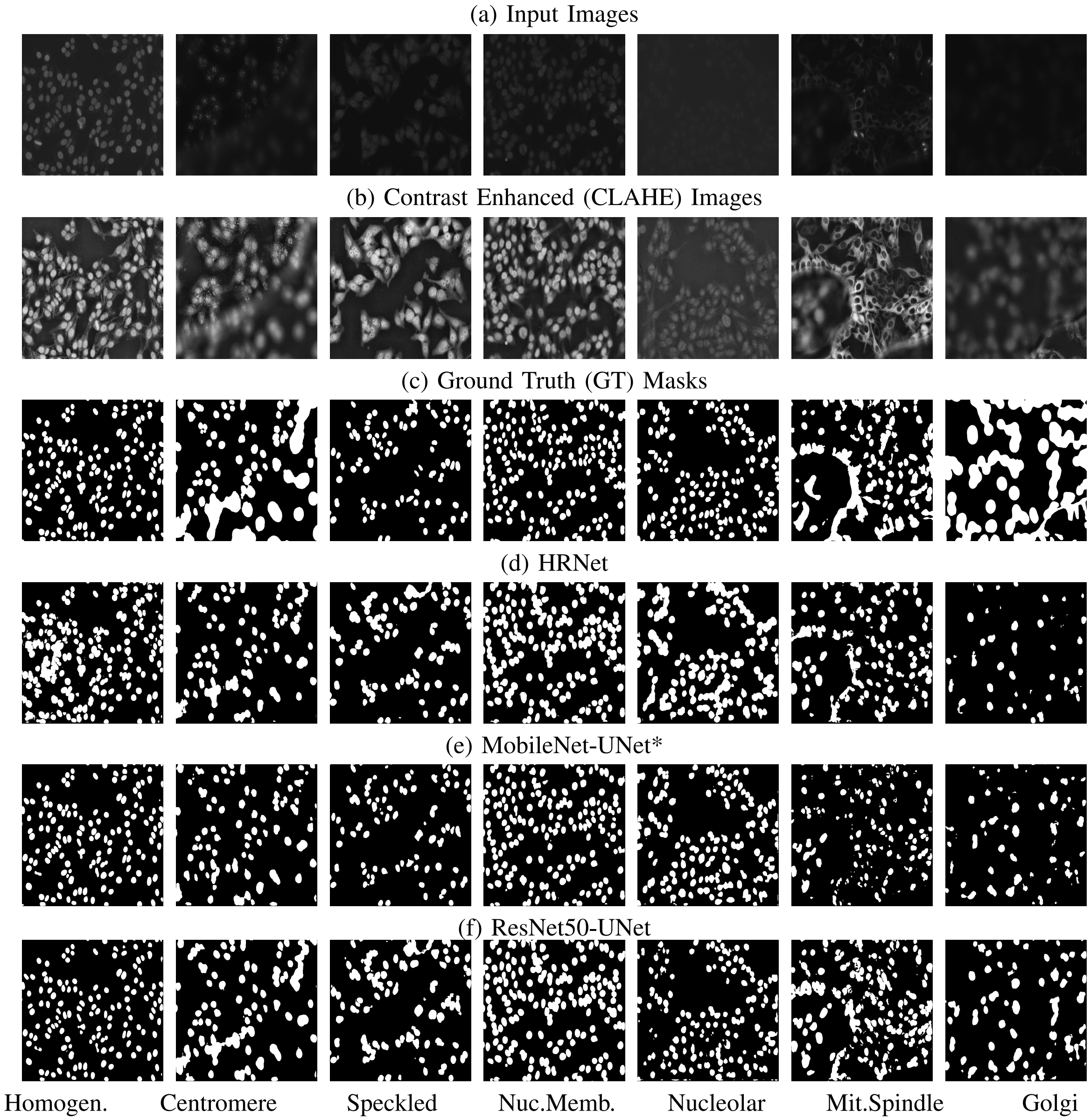

Fig. 2. Classwise segmentation results on selected bad GT HEp-2 images. (a) Input grayscale, (b) contrast enhanced (CLAHE), and corresponding (c) ground truth (GT) masks. Segmentation results of (d) HRNet, (e) MobileNet-UNet*, (f) ResNet50-UNet models. (*Pretrained).

TABLE II
CLASSWISE PERFORMANCE OF THE SELECTED TOP THREE CNN MODELS ON IMAGES PRESENTED IN FIGURE 2. (*PRETRAINED MODEL)

Model	Homogeneous	Centromere	Speckled	Nuclear Membrane	Nucleolar	Mitotic Spindle	Golgi
HRNet	0.812	0.722	0.832	0.849	0.750	0.659	0.274
MobileNet-UNet*	0.959	0.684	0.869	0.904	0.831	0.469	0.346
ResNet50-UNet	0.928	0.788	0.804	0.860	0.742	0.621	0.504

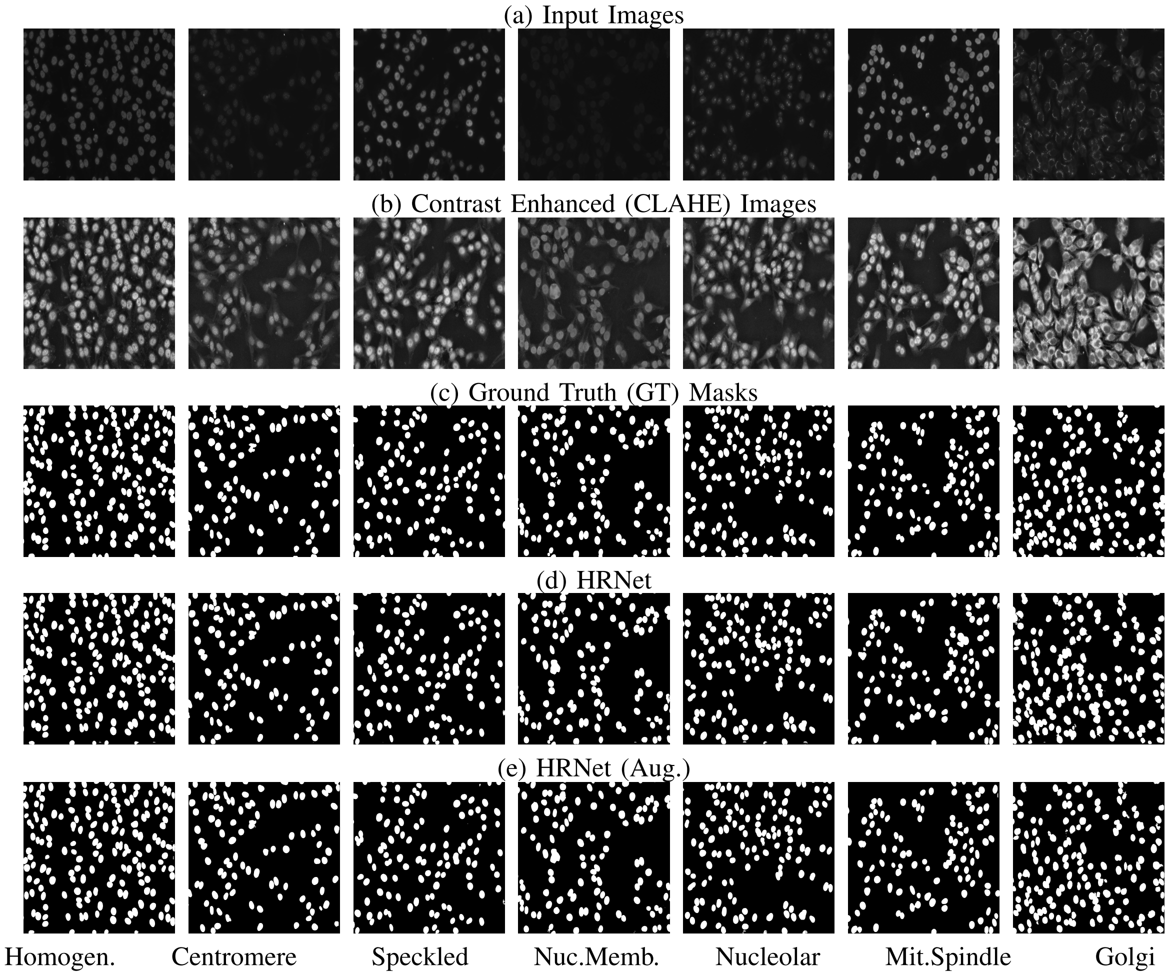

Fig. 3. Classwise representation of selected good GT HEp-2 images: (a) Input grayscale, (b) contrast enhanced (CLAHE), (c) ground truth (GT) masks, (d) HRNet, (e) HRNet with augmentation.

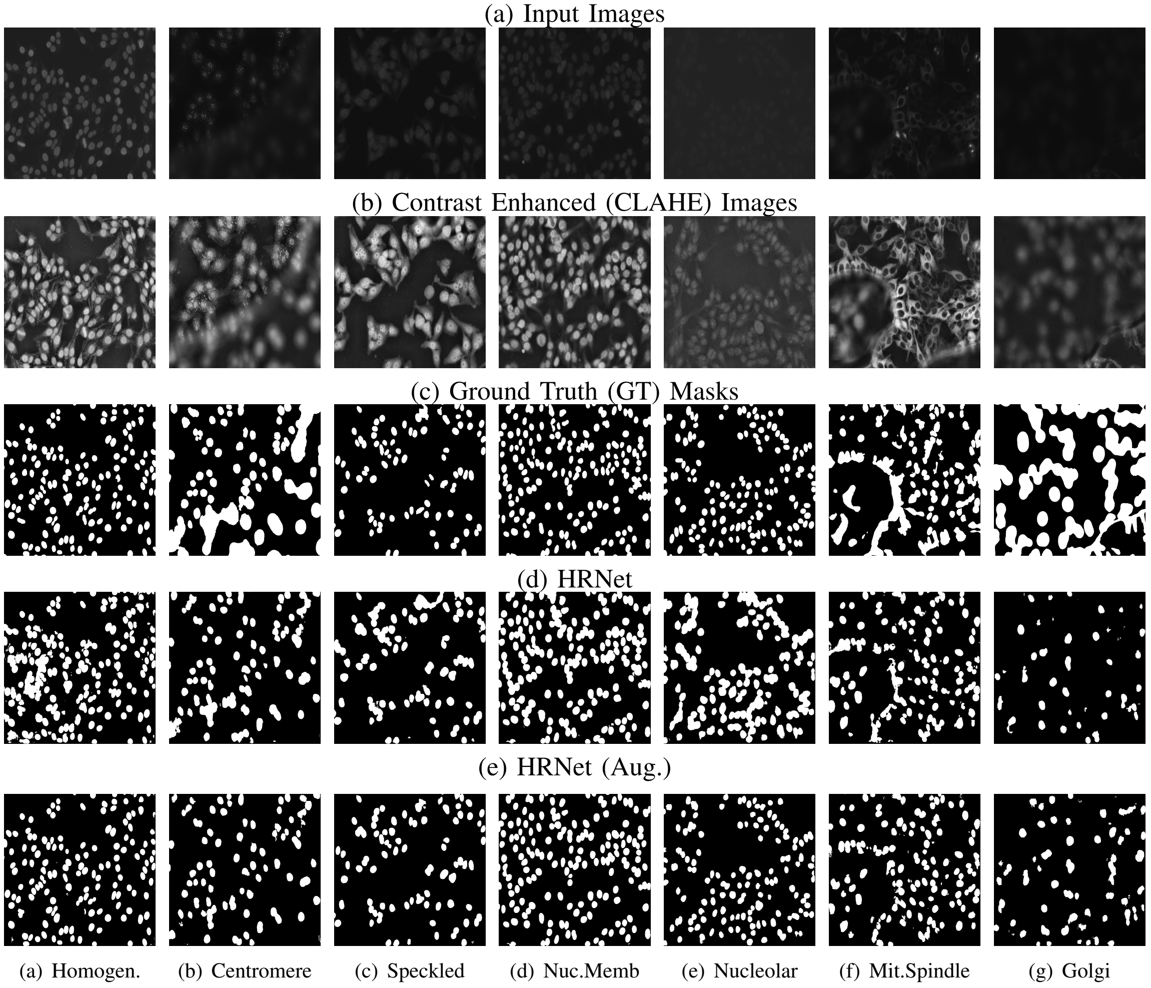

Fig. 4. Classwise segmentation results of selected bad GT HEp-2 images. (a) Input grayscale, (b) contrast enhanced (CLAHE), (c) ground truth (GT) masks, (d) HRNet, (e) HRNet with augmentation.

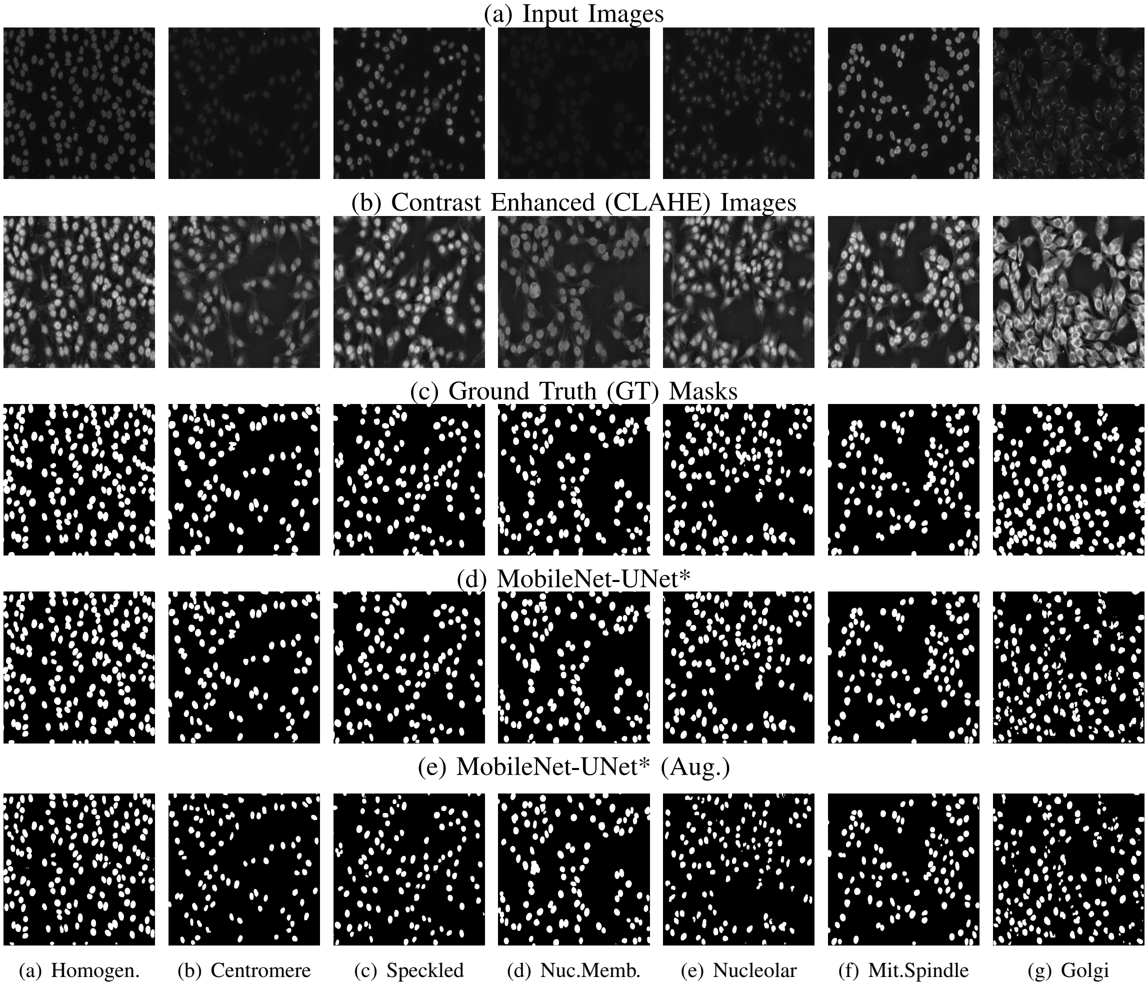

Fig. 5. Classwise representation of select good GT HEp-2 images: (a) Input grayscale, (b) contrast enhanced (CLAHE), (c) ground truth (GT) masks, (d) pretrained MobileNet-UNet, (e) pretrained MobileNet-UNet with augmentation. (*Pretrained).

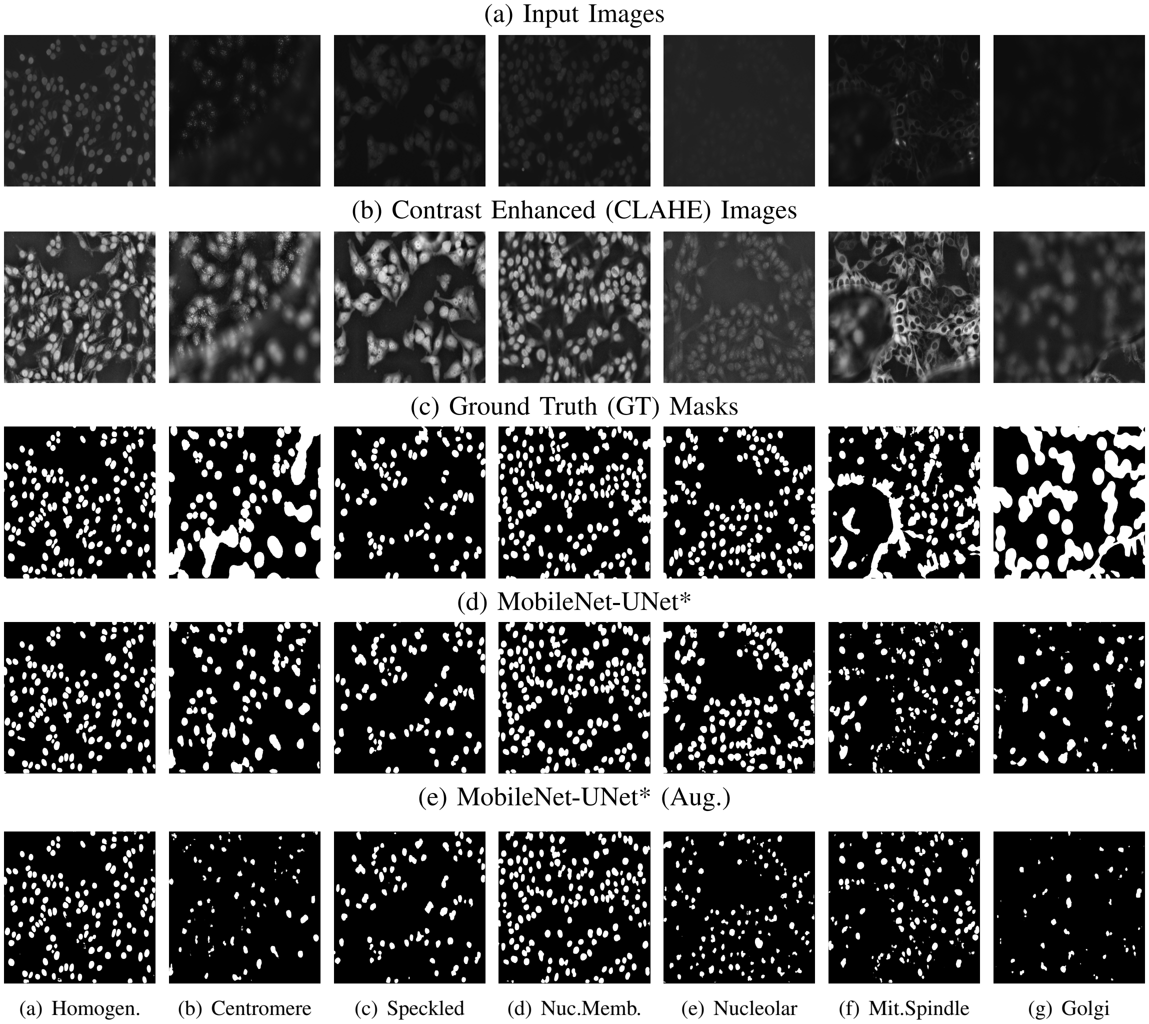

Fig. 6. Classwise representation of select bad HEp-2 images: (a) Input grayscale, (b) contrast enhanced (CLAHE), (c) ground truth (GT) masks, (d) pretrained MobileNet-UNet, (e) pretrained MobileNet-UNet with augmentation. (*Pretrained).

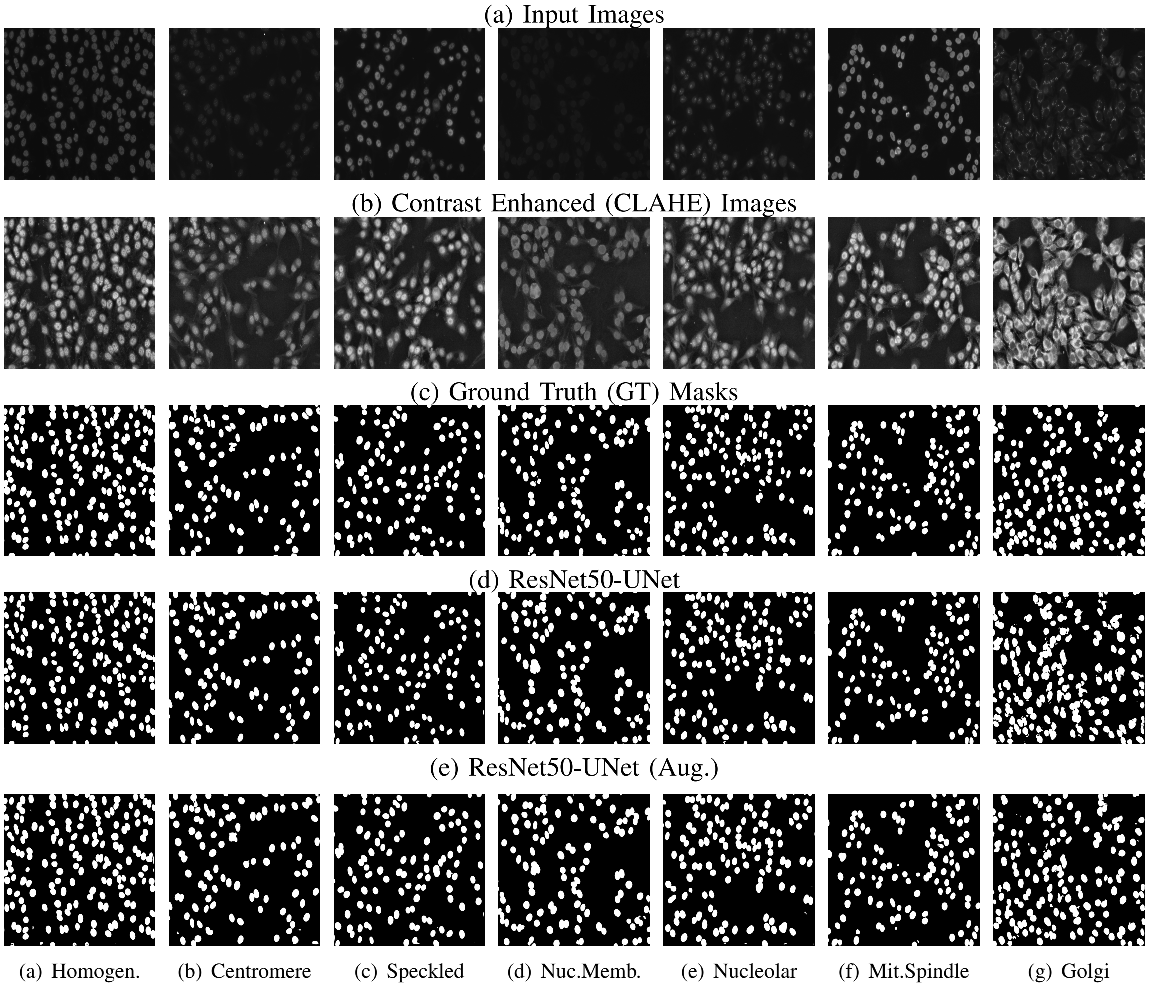

Fig. 7. Classwise representation of select good HEp-2 images: (a) Input grayscale, (b) contrast enhanced (CLAHE), (c) ground truth (GT) masks, (d) ResNet50-UNet, (e) ResNet50-UNet with augmentation. (*Pretrained).

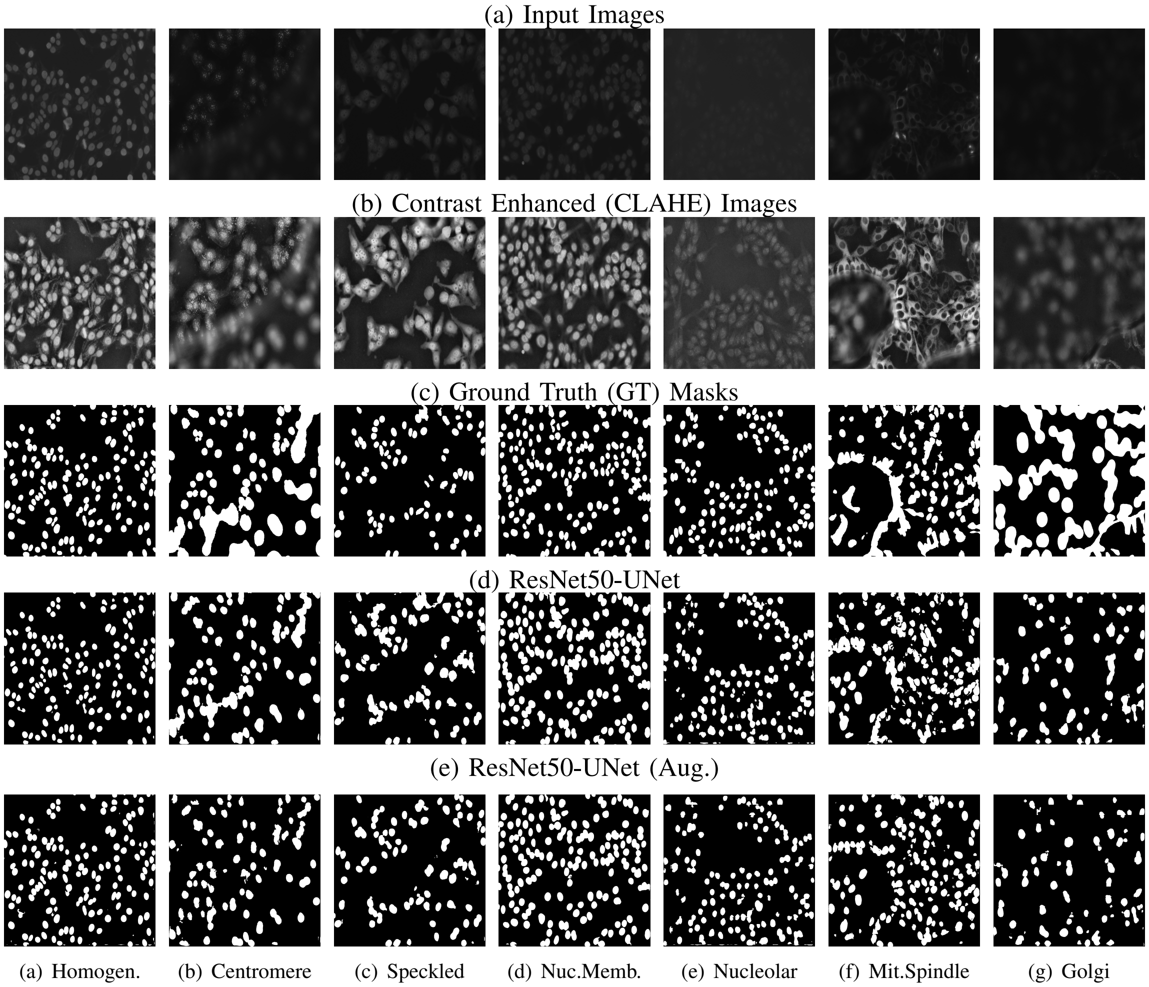

Fig. 8. Classwise representation of select bad HEp-2 images: (a) Input grayscale, (b) contrast enhanced (CLAHE), (c) ground truth (GT) masks, (d) ResNet50-UNet, (e) ResNet50-UNet with augmentation. (*Pretrained)

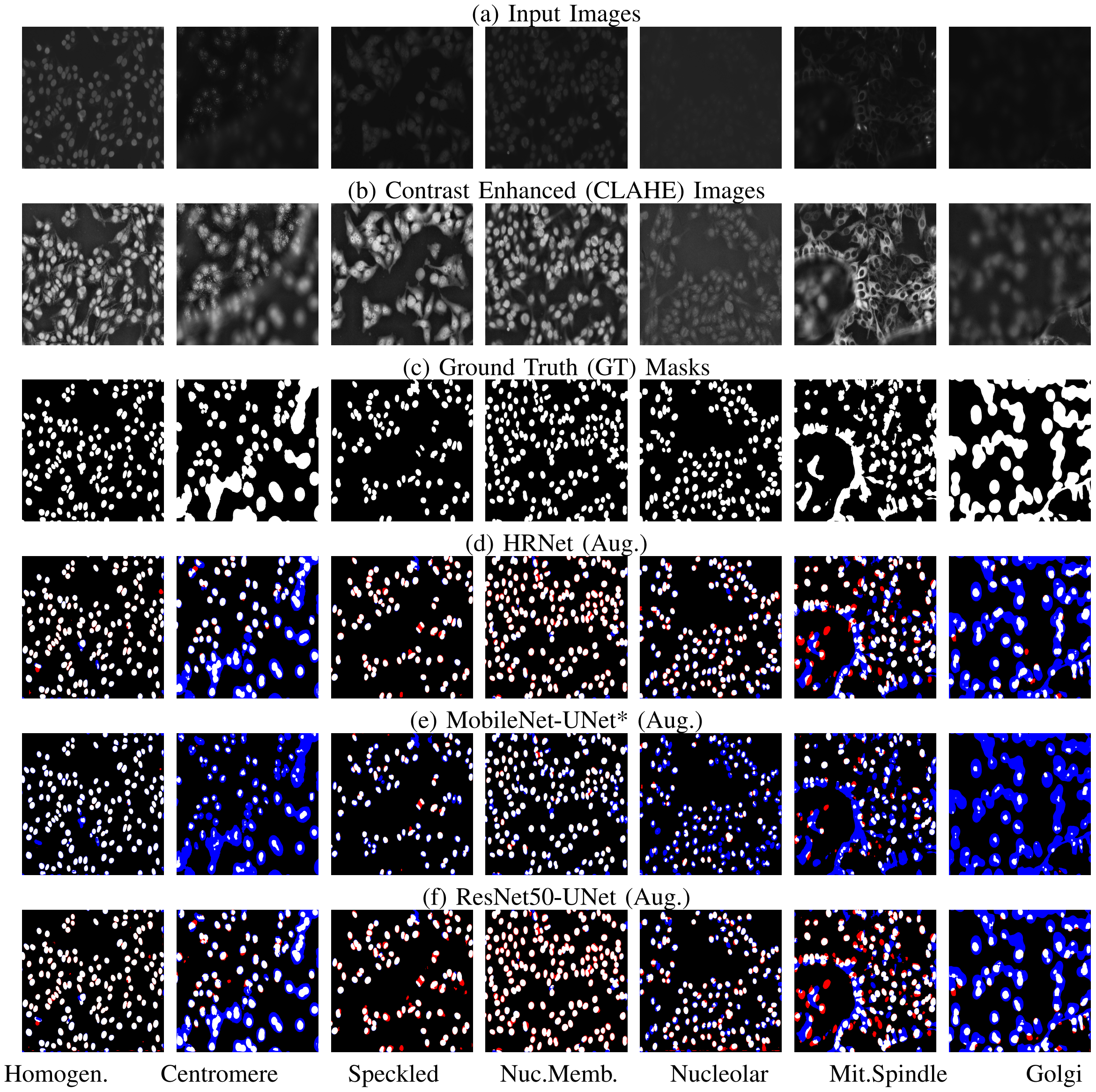

Fig. 9. Classwise segmentation results of augmented models on selected bad GT HEp-2 images. White: True Positives, Red: False Negatives, Blue: False Positives. (a) Input grayscale, (b) contrast enhanced (CLAHE), and corresponding (c) ground truth (GT) masks. Segmentation results of (d) HRNet, (e) MobileNet-UNet*, (f) ResNet50-UNet models. (*Pretrained).

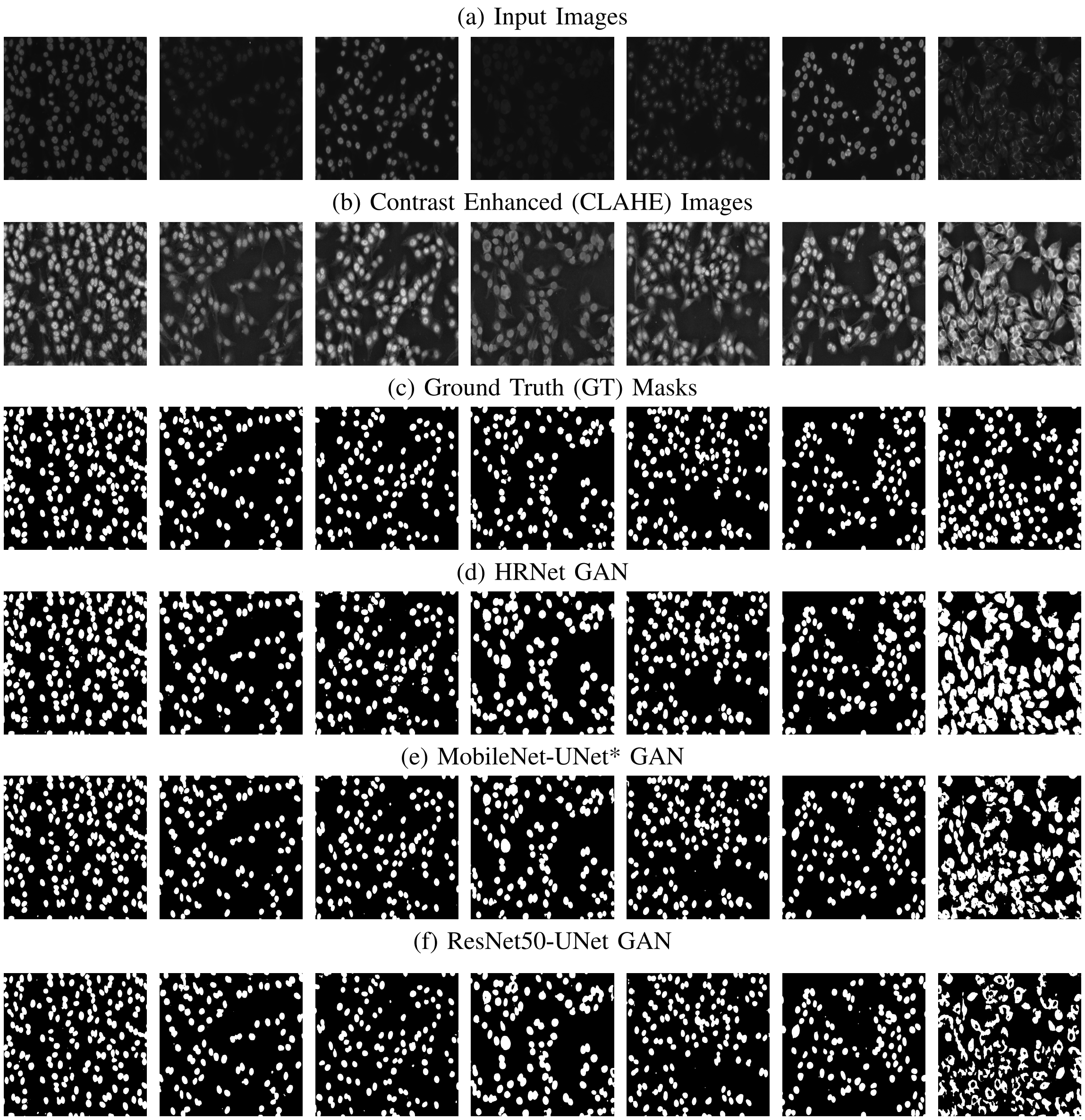

(a) Homogen. (b) Centromere (c) Speckled (d) Nuc.Memb (e) Nucleolar (f) Mit.Spindle (g) Golgi

Fig. 10. Classwise representation of select good HEp-2 images: (a) Input grayscale, (b) contrast enhanced (CLAHE), (c) ground truth (GT) masks, (d) HRNet GAN, (e) pretrained MobileNet-UNet GAN, (f) ResNet50-UNet GAN. (*Pretrained)

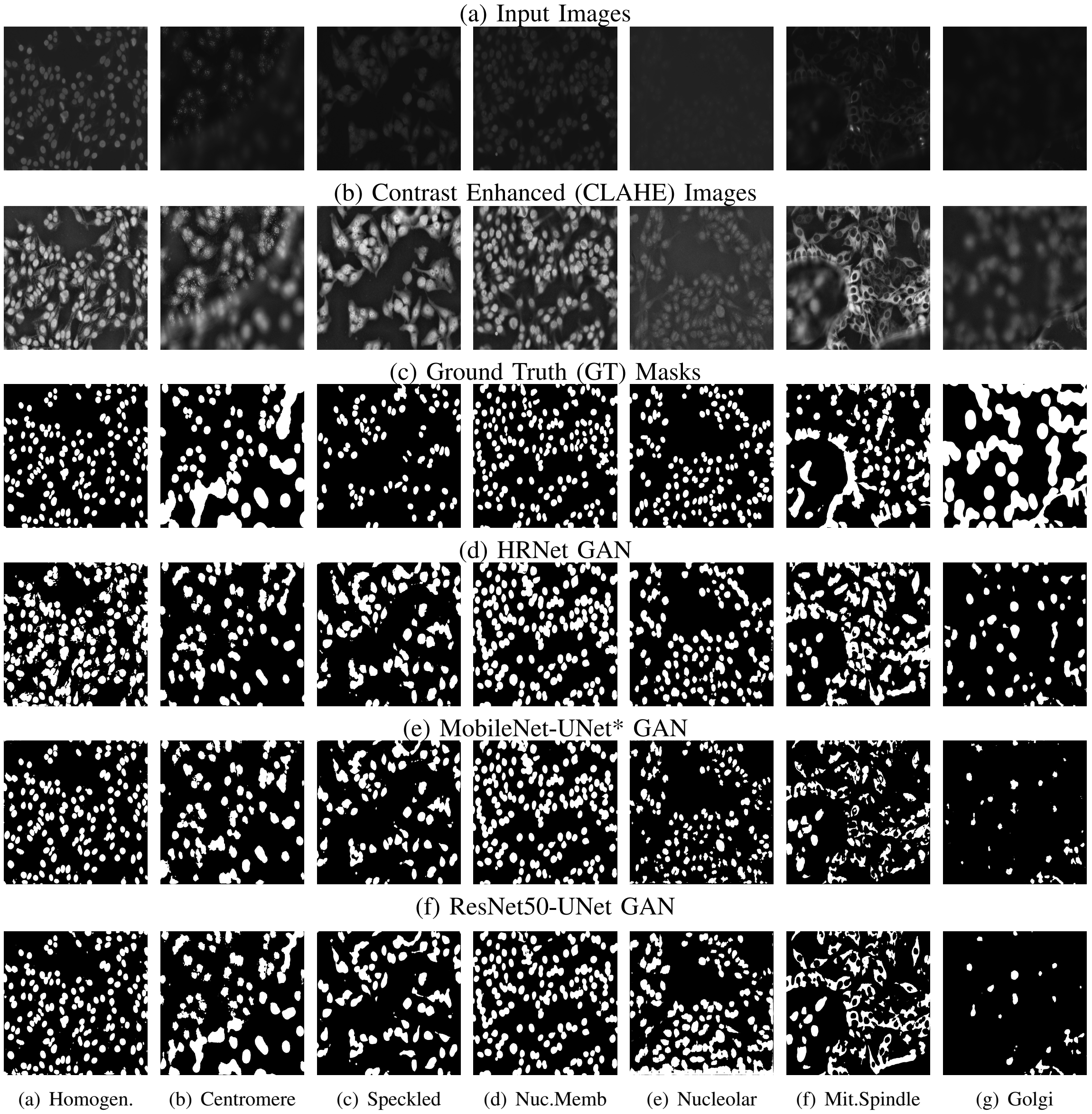

Fig. 11. Classwise representation of select bad HEp-2 images: (a) Input grayscale, (b) contrast enhanced (CLAHE), (c) ground truth (GT) masks, (d) HRNet GAN, (e) pretrained MobileNet-UNet GAN, (f) ResNet50-UNet GAN. (*Pretrained)
